# Synthesis and biological evaluation of *ortho*-phenyl phenylhydroxamic acids containing phenothiazine with improved selectivity for class IIa histone deacetylases

**DOI:** 10.1080/14756366.2024.2406025

**Published:** 2024-09-24

**Authors:** Kai-Cheng Hsu, Yun-Yi Huang, Jung-Chun Chu, Yu-Wen Huang, Jing-Lan Hu, Tony Eight Lin, Shih-Chung Yen, Jing-Ru Weng, Wei-Jan Huang

**Affiliations:** a Ph.D. Program in Drug Discovery and Development Industry, College of Pharmacy, Taipei Medical University, Taipei, Taiwan; b Graduate Institute of Cancer Biology and Drug Discovery, College of Medical Science and Technology, Taipei Medical University, Taipei, Taiwan; c Ph.D. Program for Cancer Molecular Biology and Drug Discovery, College of Medical Science and Technology, Taipei Medical University, Taipei, Taiwan; d Cancer Center, Wan Fang Hospital, Taipei Medical University, Taipei, Taiwan; e Graduate Institute of Pharmacognosy, College of Pharmacy, Taipei Medical University, Taipei, Taiwan; f Department of Marine Biotechnology and Resources, National Sun Yat-sen University, Kaohsiung, Taiwan; g Warshel Institute for Computational Biology, The Chinese University of Hong Kong (Shenzhen), Shenzhen, Guangdong, China; h School of Pharmacy, Taipei Medical University, Taipei, Taiwan

**Keywords:** Class IIa histone deacetylases (HDACs), structure-activity relationship (SAR), molecular modelling, cancer cells

## Abstract

Class IIa histone deacetylases (HDACs) have been linked to tumorigenesis in various cancers. Previously, we designed phenylhydroxamic acid **LH4f** as a potent class IIa HDAC inhibitor. However, it also unselectively inhibited class I and class IIb HDACs. To enhance the compound’s selectivity towards class IIa HDACs, the *ortho*-phenyl group from the selective HDAC7 inhibitor **1** is incorporated into *ortho* position of the phenylhydroxamic acid in **LH4f**. Compared to **LH4f**, most resulting compounds displayed substantially improved selectivity towards the class IIa HDACs. Notably, compound **7 g** exhibited the strongest HDAC9 inhibition with an IC_50_ value of 40 nM. Molecular modelling further identified the key interactions of compound **7 g** bound to HDAC9. Compound **7 g** significantly inhibited several human cancer cells, induced apoptosis, modulated caspase-related proteins as well as p38, and caused DNA damage. These findings suggest the potential of class IIa HDAC inhibitors as lead compounds for the development of cancer therapeutics.

## Introduction

Histone acetylation plays an important role in the epigenetic process by remodelling the chromatin structure, which results in altering gene expression^1^^,^^2^. This process is regulated by histone deacetylases (HDAC) and histone acetyltransferases (HAT)^3^. Dysregulation of epigenetic modifications can cause abnormal gene expression, leading to the pathogenesis of tumors^4^^,^^5^. The inhibition of HDAC activity with the use of small-molecule inhibitors has been demonstrated to be cytotoxic to many cancer cells and can produce antitumor activity *in vivo*^6–8^. Hence, the role of HDACs makes them an important target of investigation in cancer research.

The mammalian HDAC enzymes exist as 18 isoforms, making selectivity difficult to achieve. The HDAC isozymes can be divided into four distinct groups depending on their sequence homology, sub-cellular distribution, and catalytic activity^9^. Class II enzymes are further subdivided into class IIa (HDAC4, −5, −7, −9) and class IIb (HDAC6, −10). Class IIa HDACs are unique due to their weak deacetylase activity. Despite extensive research, natural substrates for class IIa HDACs remain unidentified^10^^,^^11^. Class IIa HDACs are known to play crucial biological roles in cancer cells, and their high expression is associated with poor prognosis or cancer progression^10^. For instance, HDAC7 induces deacetylation of STAT3 to inhibit STAT3 activation, thus stimulating tumorigenesis^12^. High HDAC9 expression inhibits apoptosis in breast cancer cells and can also enhance proliferation^13^. These findings highlight class IIa HDAC enzymes as potential targets in cancer therapy.

**Figure 1.**
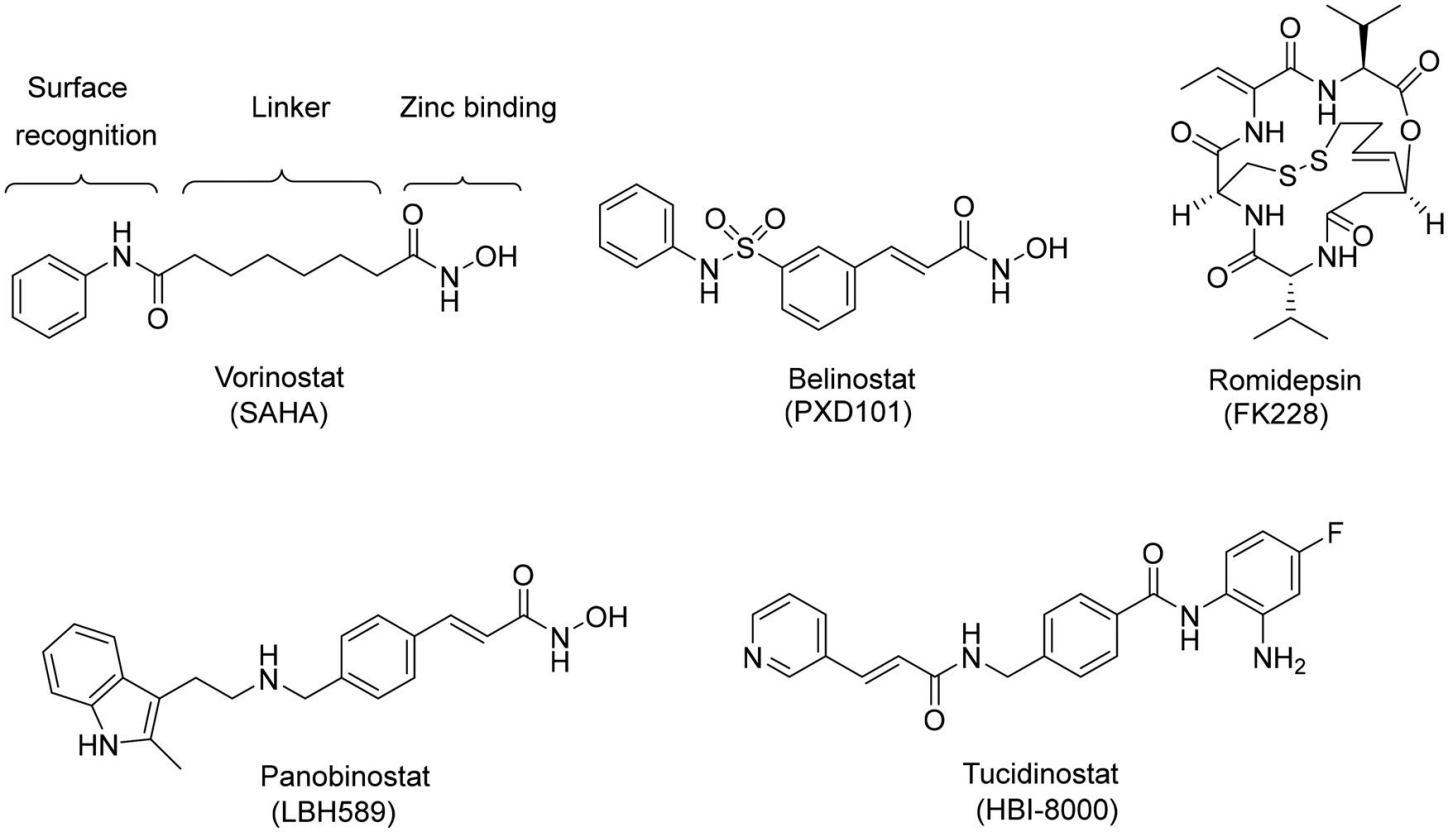
Chemical structure of clinically used HDAC inhibitors.

To date, five HDAC inhibitors have been approved for the treatment of haematologic malignancies (Figure 1). These include vorinostat, belinostat, panobinostat, tucidinostat, and romidepsin^14^. In general, HDAC inhibitors contain three distinct structural features: a zinc-binding group (ZBG), such as a hydroxamic acid, a bulky cap group to occupy the surface opening of the binding site, and a carbon linker moiety, such as aryl and aliphatic groups, that connect the two groups^15^ (Figure 1). Each group binds to a distinct domain of the HDAC active site and their structural variations can influence selectivity or potency. For example, modifications of the linker moiety can lead to the inhibition of a specific spectrum of HDACs^16^^,^^17^. The currently approved drugs are pan-inhibitory, which can lead to unfavourable side effects^18^.

**Figure 2.**
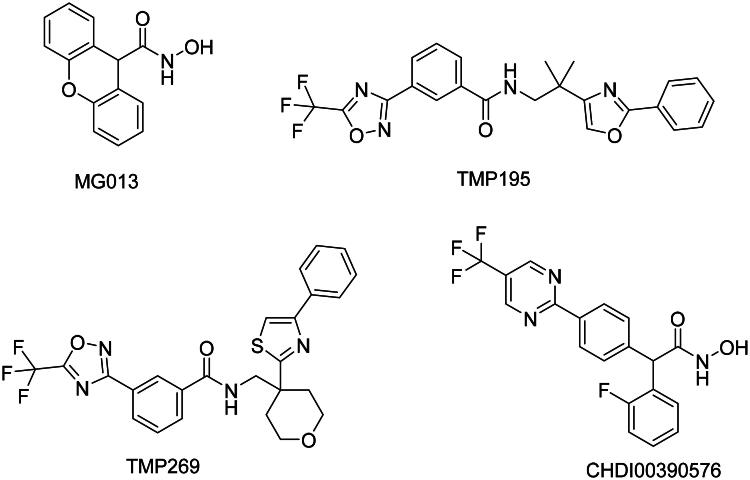
Examples of class IIa HDAC inhibitors.

**Figure 3.**
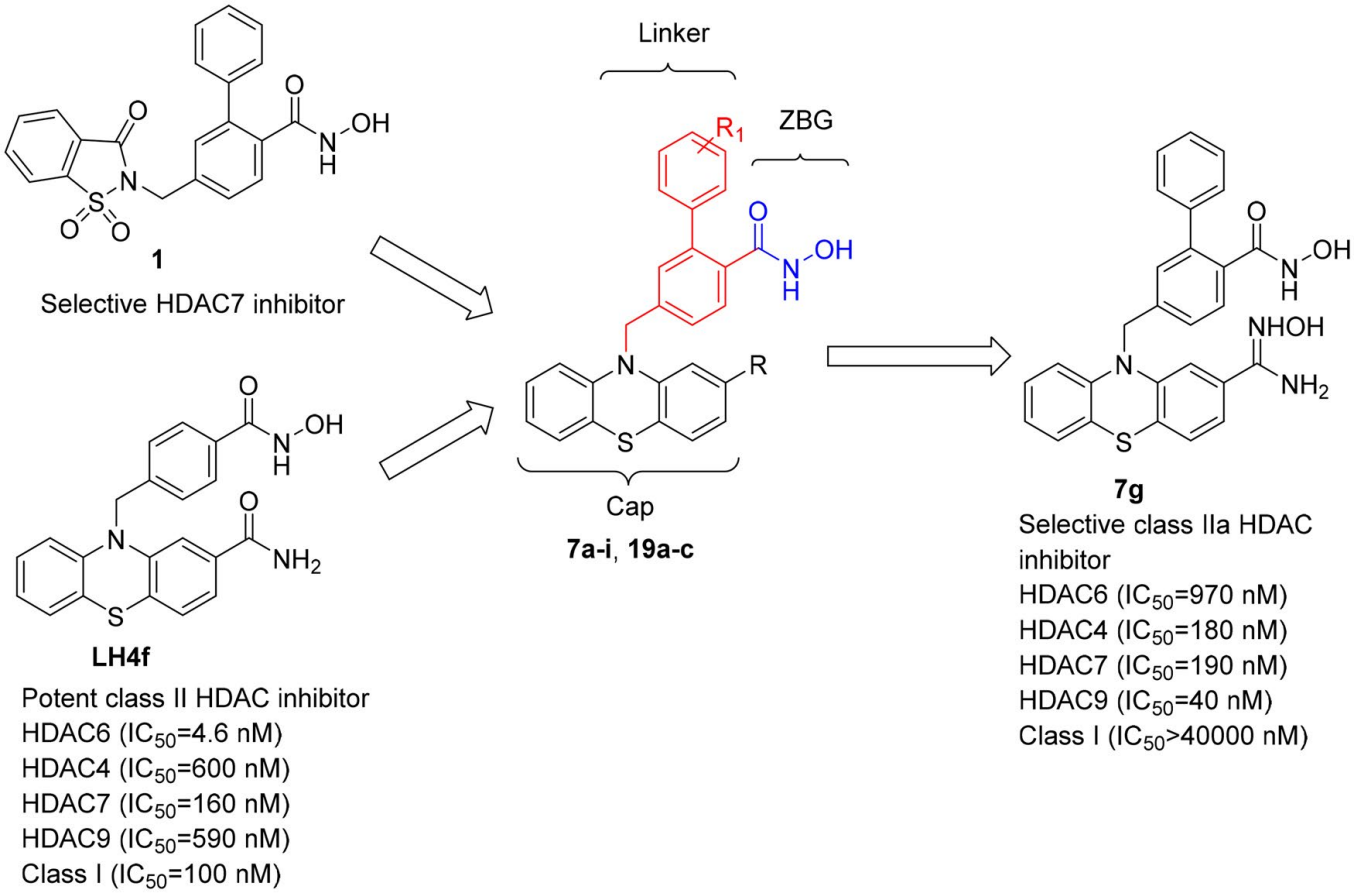
Design of compound **7 g** with improved selectivity and potency for class IIa HDACs.

Several small-molecule inhibitors targeting class IIa HDACs have been reported to display favourable cytotoxicity against different cancer cells. These structurally diverse compounds include MG013^19^, TMP195^20^, CHDI00390576^21^^,^^22^, and TMP269^23^ (Figure 2). Furthermore, the *ortho*-phenyl phenylhydroxamic acid **1**^24^ is demonstrated to selectively inhibit class IIa HDAC7 over the class I HDAC1 (Figure 3). This selectivity arises from the produced interaction between the o*rtho*-phenyl moiety and residues Phe679 and Phe738. This is a hydrophobic pocket adjacent to the Zn^2+^ active site. Previously, Vögerl and co-workers exploited phenothiazine as a cap to produce a series of benzylhydroxamic acids with potent and selective HDAC6 inhibitory activities. Some compounds have been found to exhibit strong antiproliferative activities in certain cancer cell types^25^. Additionally, we incorporated the phenothiazine ring into phenylhydroxamic acid to develop compound **LH4f**^26^ (Figure 3). Compound **LH4f** displayed much higher class IIa HDAC inhibitory activities compared to vorinostat. Despite its strong anti-class IIa HDAC activity, compound **LH4f** still retained potent activities against class I HDACs (IC_50_= 100 nM) and Class IIb HDAC (IC_50_= 5 nM). Herein, we sought to improve the class IIa selectivity of **LH4f** without compromising its inhibition of class IIa enzymes.

In this study, we introduced an *ortho*-phenyl moiety from compound **1**^24^ onto the phenylhydroxamic acid moiety of compound **LH4f** to generate compounds for the exploration of class IIa HDAC selectivity (Figure 3). This approach led to the synthesis of compounds **7a-i** and **19a-c**. Assays confirmed increased selectivity towards class IIa HDACs, with one compound exhibiting potent HDAC9 inhibition. Further analysis was conducted to confirm the cytotoxicity of the inhibitor in human oral cancer (SCC2095), breast cancer (MDA-MB-231), and gastric cancer (SC-M1) cells. These results show structural modifications that enhance HDAC selectivity and present additional options when optimising novel class IIa HDAC inhibitors.

## Materials and methods

### General procedure

NMR spectra (^1^H- and ^13^C- NMR) were obtained using the Bruker Fourier 300 and AVIII 500 spectrometer with standard plus programs. The chemical shifts were presented in parts per million (ppm, δ) with TMS as an internal standard. The MS data were measured on a Finnigan Mat TSQ-7000 mass spectrometer (ESIMS and HRESIMS). HPLC was performed using a C18 column (250 mm x 4.6 mm, Waters) and an L-2130 pump (Hitachi, Ibaraki, Japan). Column chromatography was performed on silica gel (70–230 mesh, Merck, Darmstadt, Germany). The TLC analysis was performed on silica gel plates (KG60-F254, Merck). A microplate spectrophotometer Victor 2X (Perkin Elmer Fremont, CA, United States) was used for fluorometric analysis; an Envision 2104 Multilabel Reader (Perkin Elmer, CA, United States) was used for luminescence analysis, and a Sunrise microplate reader (TECAN, Männedorf, Switzerland) was used for absorbance analysis. The HeLa nuclear extract (BML-KI140-0100) used in the enzyme inhibitory assay was purchased from Enzo Life Sciences. Unless otherwise mentioned, all chemicals and materials were used as received from commercial suppliers, including ACROS, AK Scientific, Sigma-Aldrich, Alfa Aesar, Combi-Blocks, MedChemExpress, and TCI, without any purification. The solvents were purchased from Duksan Pure Chemicals and ECHO Chemical. Anhydrous dichloromethane was distilled from calcium hydride under N_2_.

### N-Hydroxy-4-[(10H-phenothiazin-10-yl)methyl]-2-phenylbenzamide (7a)

To a solution of compound **6a** (200 mg, 0.47 mmol) in MeOH-THF (5 ml: 5 ml) was added a mixture of 50% NH_2_OH_(aq)_ (2 ml) and NaOH (94 mg, 2.36 mmol) at 0 °C. The resulting solution was warmed to RT under N_2_ for 11h. The reaction mixture was diluted with water, neutralised to pH 7–8, and extracted with EtOAC (3 × 50 ml). The combined organic layer was dried over Na_2_SO_4_, filtered and the solvent was removed *in vacuo*. The residue was purified by silica gel chromatography (Toluene: THF: AcOH= 6: 1: 0.2) to give compound **7a** (96 mg, 48%).^1^H NMR (DMSO-*d*_6_, 500 MHz) δ 10.80 (s, 1H), 8.96 (s, 1H), 7.37 (m, 3H), 7.30 (m, 5H), 7.14 (dd, *J* = 1.4, 7.5 Hz, 2H), 7.09 (m, 2H), 6.92 (m, 2H), 6.84 (d, *J* = 8.1 Hz, 1H), 5.21 (s, 2H); ^13^C NMR (125 MHz, DMSO-*d_6_*) δ 166.0, 144.2,140.0, 139.9, 138.9, 133.1, 128.8, 128.7, 128.4, 128.3, 127.7, 127.4, 127.0, 125.4, 122.9, 122.7, 115.9, 50.7, 31.0, 22.0, 14.1; HR-ESI-MS m/z: [M + H] ^+^ calcd’ for C_26_H_21_N_2_O_2_S 425.1318, found 425.1313.

### N-Hydroxy-4-[(2-chloro-10H-phenothiazin-10-yl)methyl]-2-phenylbenzamide (7b)

According to the procedure described for **7a**, reaction of compound **6b** (700 mg, 1.53 mmol) in MeOH-THF (15 ml-15 ml) added with a mixture of 50% NH_2_OH_(aq)_ (8 ml) and NaOH (306 mg, 7.65 mmol) gave compound **7b** (320 mg, 46%).^1^H NMR (DMSO-*d*_6_, 500 MHz) δ 10.82 (s, 1H), 8.97 (s, 1H), 7.40 (s, 1H), 7.38 (m, 1H), 7.37 (m, 1H), 7.33 (m, 5H), 7.16 (d, J = 8.2 Hz, 2H), 7.11 (td, J = 1.5, 7.5 Hz, 1H), 6.97 (dd, J = 2.0, 8.2 Hz, 1H), 6.94 (td, J = 1.0, 7.4 Hz, 1H), 6.90 (d, J = 2.0 Hz, 1H), 6.87 (d, J = 8.2 Hz, 1H), 5.22 (s, 2H); ^13^C NMR (DMSO-*d*_6_, 125 MHz) δ 166.0, 145.8, 143.6, 140.0, 139.9, 138.5, 133.2, 132.4, 128.8, 128.7, 128.4, 128.3, 128.1, 127.9, 127.4, 127.1, 125.4, 123.4, 122.6, 122.5, 121.9, 116.4, 115.9, 50.6, 31.0, 22.1, 14.1; HR-ESI-MS m/z: [M + H] ^+^ calcd for C_26_H_20_ClN_2_O_2_S 459.0929, found 459.0927.

### N-Hydroxy-4-{[2-(trifluoromethyl)-10H-phenothiazin-10-yl]methyl}-2-phenylbenzamide (7c)

According to the procedure described for **7a**, reaction of compound **6c** (500 mg, 1.02 mmol) in MeOH-THF (15 ml-15 ml) added with a mixture of 50% NH_2_OH_(aq)_ (8 ml) and NaOH (204 mg, 5.10 mmol) gave compound **7c** (133 mg, 27%). ^1^H NMR (DMSO-*d*_6_, 500 MHz) δ 10.80 (s, 1H), δ 8.96 (s, 1H), 7.42 (s, 1H), 7.34 (m, 8H), 6.97 (d, *J* = 7.9 Hz, 1H), 7.18 (dd, *J* = 1.3, 7.6 Hz, 1H), 7.13 (m, 2H), 6.96 (t, *J* = 7.4 Hz, 1H), 6.91 (d, *J* = 8.05 Hz, 1H), 5.28 (s, 1H); ^13^C NMR (DMSO-*d_6_*_,_ 125 MHz) δ 165.9, 145.1, 143.6, 140.0, 139.9, 138.5, 133.2, 128.9, 128.8, 128.4, 128.3, 128.1, 127.7, 127.4, 127.3, 125.5, 123.6, 122.1, 119.4, 119.4, 116.5, 112.2, 50.6, 31.0, 22.1, 14.1; HR-ESI-MS m/z: [M + H] ^+^ calcd for C_27_H_20_F_3_N_2_O_2_S 493.1192, found 493.1191.

### N-Hydroxy-4-[(2-methoxy-10H-phenothiazin-10-yl)methyl]-2-phenylbenzamide (7d)

According to the procedure described for **7a**, reaction of compound **6d** (550 mg, 1.22 mmol) in MeOH-THF (15 ml-15 ml) added with a mixture of 50% NH_2_OH_(aq)_ (8 ml) and NaOH (244 mg, 6.10 mmol) gave compound **7d** (383 mg, 70%). ^1^H NMR (DMSO-*d*_6_, 500 MHz) δ 10.80 (s, 1H), 8.95 (s, 1H), 7.41 (s, 1H), 7.39 (m, 2H), 7.32 (m, 5H), 6.91 (dd, *J* = 1.5, 7.6 Hz, 1H), 7.09 (td, *J* = 1.5, 7.5 Hz, 1H), 7.04 (d, *J* = 8.4 Hz, 1H), 6.91 (td, *J* = 1.1, 7.5 Hz, 1H), 6.86 (d, *J* = 8.2 Hz, 1H), 6.53 (dd, *J* = 2.5, 8.5 Hz, 1H), 6.42 (d, *J* = 2.5 Hz, 1H), 5.21 (s, 2H), 3.62 (s, 3H); ^13^C NMR (DMSO-*d_6_*, 125 MHz) δ 166.0, 159.4, 145.6, 144.1, 139.9, 139.9, 139.0, 133.1, 128.7, 128.4, 128.3, 127.5, 127.4, 126.9, 125.4, 123.4, 122.8, 116.0, 113.5, 107.4, 103.7, 55.2, 50.7, 31.0, 22.1, 14.0; HR-ESI-MS m/z: [M + H] ^+^ calcd for C_27_H_23_N_2_O_3_S 455.1424, found 455.1421.

### N-Hydroxy-5-{[2-(methylthio)-10H-phenothiazin-10-yl]methyl}-2-phenylbenzamide (7e)

According to the procedure described for **7a**, reaction of compound **6e** (470 mg, 1.00 mmol) in MeOH-THF (15 ml-15 ml) added with a mixture of 50% NH_2_OH_(aq)_ (8 ml) and NaOH (200 mg, 5.00 mmol) gave compound **7e** (277 mg, 59%). ^1^H NMR (DMSO-*d*_6_, 500 MHz) δ 10.80 (s, 1H), 8.95 (s, 1H), 7.42 (s, 1H), 7.38 (m, 2H), 7.33 (m, 5H), 7.16 (dd, *J* = 1.5, 7.6 Hz, 1H), 7.11 (td, *J* = 1.5, 6.6 Hz, 1H), 7.08 (d, *J* = 8.1 Hz, 1H), 6.93 (td, *J* = 1, 7.5 Hz, 1H), 6.90 (d, *J* = 8.2 Hz, 1H), 6.81 (dd, *J* = 1.8, 8.1 Hz, 1H), 6.72 (d, *J* = 1.8 Hz, 1H), 5.23 (s, 2H), 2.31 (s, 3H); ^13^C NMR (DMSO-*d_6_*_,_ 125 MHz) δ 165.9, 144.7, 144.2, 139.9, 139.8, 138.9, 137.6, 133.0, 128.8, 128.7, 128.3, 128.2, 127.6, 127.3, 127.1, 127.0, 125.4, 123.1, 122.9, 120.3, 119.3, 116.1, 113.8, 50.5, 14.8; HR-ESI-MS m/z: [M + H] ^+^ calcd for C_27_H_23_N_2_O_2_S_2_ 471.1195, found 471.1197.

### N-Hydroxy-4-{2-[1-(hydroxyimino)ethyl]-10H-phenothiazin-10-yl}methyl-2-phenylbenzamide (7f)

According to the procedure described for **7a**, reaction of compound **6f** (350 mg, 0.76 mmol) in MeOH-THF (10 ml-10 ml) added with a mixture of 50% NH_2_OH_(aq)_ (8 ml) and NaOH (152 mg, 3.80 mmol) gave compound **7f** (185 mg, 52%). ^1^H NMR (DMSO-*d*_6_, 500 MHz) δ 11.18 (s, 1H), 10.81 (s, 1H), 8.96 (s, 1H), 7.41 (s, 1H), 7.37 (m, 2H), 7.31 (m, 5H), 7.19 (dd, *J* = 1.5, 8.0 Hz, 1H), 7.15 (m, 3H), 7.08 (td, *J* = 1.5, 8.1 Hz, 1H), 6.92 (td, *J* = 0.9, 7.5 Hz, 1H), 6.82 (d, *J* = 8.1 Hz, 1H), 5.23 (s, 2H), 2.01 (s, 3H); ^13^C NMR (DMSO-*d_6_*_,_ 125 MHz) δ 165.9, 152.5, 144.2, 143.9, 139.9, 139.9, 138.8, 136.4, 133., 128.7, 128.6, 128.3, 128.2, 127.6, 127.3, 126.9, 126.7, 125.3, 123.6, 122.9, 122.5, 120.3, 116.1, 112.6, 50.8, 11.5; HR-ESI-MS m/z: [M + H] ^+^ calcd for C_28_H_24_N_3_O_3_S 482.1533, found 482.1534.

### Synthesis of compounds 7 g and 7h

According to the procedure described for **7a**, reaction of compound **6 g** (500 mg, 1.12 mmol) in MeOH-THF (10 ml-10 ml) added with a mixture of 50% NH_2_OH_(aq)_ (6 ml) and NaOH (223 mg, 5.58 mmol) gave compounds **7 g** (196 mg, 36%) and **7h** (51 mg, 9%).

### N-Hydroxy-4-[2-(N’-hydroxycarbamimidoyl)-10H-phenothiazin-10-yl]methyl-2-phenylbenzamide (7 g)

^1^H NMR (DMSO-*d*_6_, 500 MHz) δ 10.85 (s, 1H), 9.61 (s, 1H), 8.97 (s, 1H), 7.41 (s, 1H), 7.38 (dt, *J* = 1.2, 7.2 Hz, 1H), 7.36 (s, 1H), 7.34 (s, 1H), 7.33 (m, 1H), 7.32 (s, 1H), 7.31 (d, *J* = 0.8 Hz, 1H), 7.22 (dd, *J* = 1.6, 8 Hz, 1H), 7.16 (d, *J* = 1.4 Hz, 1H), 7.14 (s, 1H), 7.13 (s, 1H), 7.07 (td, *J* = 1.5, 7.5 Hz, 1H), 6.90 (td, *J* = 1, 7.5 Hz, 1H), 6.78 (dd, *J* = 0.7, 8.2 Hz, 1H), 5.77 (s, 2H), 5.25 (s, 2H); ^13^C NMR (DMSO-*d_6_*, 125 MHz) δ 166.0, 150.6, 144.1, 143.8, 139.9, 139.9, 138.8, 133.0, 132.9, 128.8, 128.6, 128.4, 128.3, 127.7, 127.4, 126.9, 126.5, 125.2, 123.5, 122.9, 122.3, 120.0, 116.1, 112.6, 50.7, 31.0, 22.1, 14.0. HR-ESI-MS m/z: [M + H] ^+^ calcd for C_27_H_23_N_4_O_3_S 483.1485, found 483.1489.

### N-Hydroxy-4-[(2-carbamoyl-10H-phenoxazin-10-yl)methyl]-2-phenylbenzamide (7h)

^1^H NMR (DMSO-*d*_6_, 500 MHz) δ 10.83 (s, 1H), 8.96 (s, 1H), 7.93 (s, 1H), 7.43 (d, *J* = 9.6 Hz, 2H), 7.37 (s, 1H), 7.36 (m, 4H), 7.34 (s, 1H), 7.32 (s, 4H), 7.21 (d, *J* = 7.9 Hz, 1H), 7.15 (dd, *J* = 1.2, 7.5 Hz, 1H), 7.09 (td, *J* = 1.2, 8.1 Hz, 1H), 6.92 (t, *J* = 7.4 Hz, 1H), 6.84 (d, *J* = 8.1 Hz, 1H), 5.26 (s, 2H); ^13^C NMR (DMSO-*d_6_*_,_ 125 MHz) δ 167.4, 165.9, 144.2, 143.7, 139.9, 139.9, 138.7, 133.7, 133.1, 128.7, 128.7, 128.3, 128.2, 127.8, 128.3, 127.0, 126.7, 126.5, 125.2, 123.0, 122.0, 121.9, 116.1, 114.6, 50.6, 30.7; HR-ESI-MS m/z: [M + H] ^+^ calcd for C_27_H_22_N_3_O_3_S 468.1376, found 468.1378.

### N-Hydroxy-4-[(adamantan-1-ylamino)methyl]-2-phenylbenzamide (7i)

According to the procedure described for **7a**, reaction of compound **9** (200 mg, 0.53 mmol) in MeOH-THF (10 ml-10 ml) added with the mixture of 50% NH_2_OH_(aq)_ (6 ml) and NaOH (107 mg, 2.66 mmol) gave **7i** (40 mg, 20%). ^1^H NMR (DMSO-*d*_6_, 500 MHz) δ 10.71 (s, 1H), 7.34 (m, 8H), 3.77(s, 2H), 1.63 (d, *J* = 12.3 Hz, 8H), 1.57 (d, *J* = 11.6 Hz, 3H); ^13^C NMR (DMSO-*d_6_*, 125 MHz) δ 172.5, 166.6, 140.8, 139.8, 132.9, 130.0, 128.8, 128.6, 128.5, 127.5, 126.9, 51.0, 44.2, 42.5, 36.7, 29.4, 21.6; HR-ESI-MS m/z: [M + H] ^+^ calcd for C_24_H_29_N_2_O_2_ 377.2224, found 377.2217.

### N-Hydroxy-4-[(1,3,4,5-tetrahydro-2H-benzo[c]azepin-2-yl)methyl]-2-phenylbenzamide (7j)

According to the procedure described for **7a**, reaction of compound **11** (400 mg, 1.076 mmol) in MeOH-THF (10 ml-10 ml) added with a mixture of 50% NH_2_OH_(aq)_ (6 ml) and NaOH (215 mg, 5.38 mmol) at 0 °C gave **7j** (40 mg, 20%). ^1^H NMR (DMSO-*d*_6_, 500 MHz) δ 10.74 (s, 1H), 8.94 (s, 1H), 7.39 (s, 2H), 7.38 (s, 2H), δ 7.33 (m, 2H), δ 7.26 (m, 2H), δ 7.14 (m, 2H), δ 7.06 (td, *J* = 1.7, 6.7 Hz, 1H), δ 6.95 (d, *J* = 7.2 Hz, 1H), δ 3.83 (s, 2H), δ 3.54 (s, 2H), δ 3.01 (t, *J* = 4.8 Hz, 2H), δ 2.88 (t, *J* = 5 Hz, 2H), δ 1.65 (m, 2H). ^13^C NMR (DMSO-*d_6_*_,_ 125 MHz) δ 166.5, 143.2, 143.5, 140.6, 140.2, 139.7, 133.3, 130.5, 129.9, 128.1, 128.7, 128.7, 128.6, 127.8, 127.5, 127.4, 126.1, 59.5, 58.4, 57.3, 35.6, 25.5. HR-ESI-MS m/z: [M + H] ^+^ calcd for C_24_H_25_N_2_O_2_ 373.1911, found 373.1903.

### N-Hydroxy-4-[(2-cyano-10H-phenothiazin-10-yl)methyl]-2–(3,4,5-trimethoxyphenyl)benzamide (19a)

To a solution of compound **18a** (120 mg, 0.23 mmol), and BOP (102 mg, 0.23 mmol) in dry DMF (10 ml) was added DIPEA (80 µL, 0.46 mmol) at 0 °C. The resulting solution was stirred for 15 min and then was added with NH_2_OH (17 mg, 0.25 mmol). The reaction mixture was stirred for additional 1 h. The reaction mixture was diluted with distd H_2_O_2_ (50 ml), acidified with 1 N HCl_(aq)_ to pH = 3 and extracted with EtOAc (3 × 50 ml). The combined organic layer was dried over Na_2_SO_4,_ filtered and the solvent was concentrated *in vacuo*. The residue was purified by silica gel chromatography (MeOH: CH_2_Cl_2_ = 1: 99) to give compound **19a** (70 mg, 56%). ^1^H NMR (DMSO-*d*_6_, 300 MHz) δ 10.84 (d, *J* = 1.4 Hz, 1H), 9.01 (d, *J* = 1.6 Hz, 1H), 7.45 (s, 1H), 7.33 (m, 4H), 7.23 (s, 1H), 7.18 (m, 2H), 6.97 (m, 1H), 6.89 (d, *J* = 8.0 Hz, 1H), 6.63 (s, 2H), 5.26 (s, 2H), 3.76 (s, 6H), 3.68 (s, 3H). ^13^C NMR (DMSO-*d_6_*_,_ 125 MHz) δ 166.6, 153.0, 145.2, 143.6, 140.2, 138.3, 137.3, 135.7, 133.8, 130.6, 129.1, 128.8, 128.6, 128.2, 127.6, 126.9, 125.7, 124.0, 121.9, 119.3, 118.7, 117.0, 110.4, 106.1, 60.4, 56.2, 50.9. HR-ESI-MS m/z: [M + H] ^+^ calcd for C_30_H_26_N_3_O_5_S 540.1588, found 540.1586.

### N-Hydroxy-4-[(2-cyano-10H-phenothiazin-10-yl)methyl]-2–(4-methoxyphenyl)benzamide (19b)

Following the procedure as described for **19a**, reaction of compound **18b** (270 mg, 0.58 mmol), BOP (257 mg, 0.58 mmol), NH_2_OH (44.4 mg, 0.64 mmol), and DIPEA (202 µL, 1.16 mmol) in DMF (10 ml) gave compound **19b** (133 mg, 48%). ^1^H NMR (DMSO-*d*_6_, 300 MHz) δ 10.76 (s, 1H), 8.93 (s, 1H), 7.37 (m, 3H), 7.27(m, 5H), 7.15 (m, 2H), 6.96 (m, 3H), 6.88 (d, *J* = 8.2 Hz, 1H), 5.25 (s, 2H), 3.78 (s, 3H). ^13^C NMR (DMSO-*d_6_*, 125 MHz) δ 166.5, 159.2, 145.2, 143.6, 140.0, 138.4, 133.5, 132.6, 130.6, 129.9, 129.2, 128.9, 128.6, 128.2, 127.6, 126.9, 125.2, 124.0, 121.9, 119.3, 118.6, 116.9, 114.3, 110.4, 55.6, 51.0. HR-ESI-MS m/z: [M + H] ^+^ calcd for C_28_H_22_N_3_O_3_S 480.1376, found 480.1374.

### N-Hydroxy-4-[(2-cyano-10H-phenothiazin-10-yl)methyl]-2–(4-fluorophenyl)benzamide (19c)

Following the procedure as described for **19a**, reaction of compound **18c** (100 mg, 0.22 mmol), BOP (97 mg, 0.22 mmol), NH_2_OH (17 mg, 0.24 mmol), and DIPEA (77 µL, 0.44 mmol) in DMF (10 ml) gave compound **19c** (75 mg, 73%). ^1^H NMR (DMSO-*d*_6_, 300 MHz) δ 10.81 (d, *J* = 1.4 Hz, 1H), 8.96 (d, *J* = 1.5 Hz, 1H), 7.43 (s, 1H), 7.37 (m, 4H), 7.33 (m, 2H), 7.23 (m, 3H), 7.18 (dd, *J* = 1.5, 7.6 Hz, 1H), 7.14 (m, 1H), 7.96 (td, *J* = 1.0, 7.5 Hz, 1H), 6.89 (d, *J* = 8.2 Hz, 1H), 5.26 (s, 2H). ^13^C NMR (DMSO-*d_6_*, 125 MHz) δ 166.1, 161.2, 145.2, 143.6, 139.3, 138.7, 136.7, 133.7, 130.7, 130.7, 129.2, 129.2, 128.6, 128.2, 127.6, 126.9, 125.8, 124.0, 121.9, 119.2, 118.7, 116.9, 115.7, 115.5, 110.4, 50.9. HR-ESI-MS m/z: [M + H] ^+^ calcd for C_27_H_19_N_3_O_2_FS 468.1177, found 468.1176.

### HDAC inhibition assay

The HDAC activity assay was performed as according to previously described method^27–30^. Enzymes, inhibitors, and substrates were diluted with HDAC buffer (15 mM Trise HCl pH 8.1, 0.25 mM EDTA, 250 mM NaCl, 10% v/v glycerol). Briefly, 10 μL diluted HDAC such as HeLa nuclear extract (Enzo), HDAC4, HDAC6 (BPS), HDAC7, HDAC9, HDAC8, and 50 μL test compound solution at different concentrations were added to each well of a 96 well microtiter plate and pre-incubated at 37 °C for 5 min. The enzymatic reaction was started by the addition of 40 ml substrate such as Boc-Lys-(AMC) (BPS) for HeLa nuclear extract and HDAC6, Boc-Lys(TFA)-AMC (Bachem) for HDACs 4, 8, and 50040 (BPS) for HDACs 7, 9 in HDAC buffer. After incubation at 37 °C for 30 min, the reaction was terminated by the addition of 100 μL trypsin solution (10 mg/mL trypsin in 50 mM Tris HCl pH 8, 100 mM NaCl, 2 mM vorinostat). After another incubation at 37 °C for 20 min, fluorescence was measured (excitation λ = 355 nm, emission λ = 460 nm). For the calculation of IC_50_ values, the fluorescence in wells without test compound (0.1% DMSO, negative control) was set at 100% enzymatic activity and the fluorescence in wells with 2 mM vorinostat (positive control) was set at 0% enzymatic activity. All experiments were carried out in replicates.

### Cell culture

Human breast cancer MDA-MB-231 cells and gastric cancer SCM-1 cells were purchased from the American Type Culture Collection (Manasas, VA, USA). SCC2095 human oral cancer cells were kindly provided by Professor Susan R. Mallery (The Ohio State University). Normal human lung fibroblasts (NHLFs; CC-2512) were purchased from the Lonza (Walkersville, MD, USA). SCM-1 cells were maintained in RPMI-1640 medium (Invitrogen, Carlsbad, CA, USA); MDA-MB-231 and SCC2095 cells were maintained in DMEM/F12 (Invitrogen); and NHLFs were maintained in FGM^™^-2 medium (Lonza**)** with 2% serum, supplemented with 10% heat-inactivated foetal bovine serum (FBS; Gibco, Grand Island, NY, USA). All cells were cultured at 37 °C in a humidified incubator containing 5% CO_2_ and 95% relative humidity.

### Cell viability assay

To assess cell viability, cells were seeded in three replicates into 96-well plates at a density of 5×10^3^/well for 24 h following incubation with compounds at the indicated concentrations. Then, cell viability was assessed with 3–(4,5-dimethylthiazol-2-yl)-2,5-diphenyltetrazolium bromide (MTT) solution (0.5 mg/mL) for 4h as described previously^31^. After removing the medium, 200 µL of DMSO was added, and then the absorbance was measured at 570 nm using a plate reader (BMG LABTECH, Germany).

### Western blotting

The protein expression was performed as previously described^31^. Proteins were resolved on 10% SDS-PAGE, then transferred to PVDF (Bio-Rad) membranes. After blocking with 5% skimmed milk in PBS containing 0.1% Tween 20, the membranes were probed with primary antibodies against interest target proteins at 4 °C overnight. The following primary antibodies were used: pro-caspase 8, cleaved caspase 9, acetyl α-tubulin, Hsp90, Histone H2A.X, p38, p-^180^Thr/^182^Tyr p38, PPARγ, and p21 were purchased from Cell Signalling Technologies (Beverly, MA, USA); pro-caspase 8, Millipore; β-actin, Sigma-Aldrich (St. Louis, MO, USA). Then, the immunoblots were washed and exposed to HRP-conjugated secondary antibodies at room temperature for 1 h. The immunoblots were examined by enhanced chemiluminescence.

### Flow cytometric analysis

Cells (2 × 10^5^) were treated with tested compounds in 6-well plates for 24 h or 48 h, then washed with ice-cold PBS twice and harvested with trypsin. Cells were incubated with 5 µL Annexin V- FITC/PI (BD Pharmingen, San Diego) for 15 min in the dark condition^31^. Then, the cells were measured by BD FACSCanto flow cytometer (BD, Franklin Lakes, NJ). For ROS determination, DMSO or H_2_O_2_ or compound **7 g**-treated cells were stained with the ROS probe (DCFH-DA; 5 µM) and detected the DCFH-DA fluorescence.

### Statistically analysis

All statistical analyses were conducted in triplicate and expressed as the mean ± SD. Student’s *t* test was performed to analyse the differences between control and treated groups. *P*-values <0.05 was considered significant.

## Molecular docking analysis

The HDAC structures for this study were obtained from the Protein Data Bank (PDB)^32^. The structures include HDAC1 (PDB ID: 5ICN), HDAC2 (PDB ID: 4lXZ), and HDAC3 (PDB ID: 4A69). At the time of the study, an HDAC9 crystal structure was unavailable. As such, a homology model was generated^33^. The amino acid sequence for HDAC9 was obtained from the GeneBank public repository^34^. The sequence was then uploaded onto the SWISS-MODEL server (https://swissmodel.expasy.org) to model the HDAC9 structure. The server contains a template library from public sources, such as the Protein Data Bank^32^, that is mined to identify suitable alignments between the target sequence and the template structure. The structure of HDAC4 (PDB ID: 2VQM) was utilised as the template to model the structure of HDAC9 for further analysis. Molecular docking was performed using the LeadIT software^35^. The software was used to prepare both the protein and compound **7 g** for docking. Hydrogens and relevant charges were added to the HDAC structure using default methods. The structure of compound **7 g** was prepared by generating 3D conformations and protonated in an aqueous solution. The docking experiment was performed using a hybrid enthalpy/entropy docking strategy at default parameters. The protein-ligand interactions were generated in Pipeline Pilot using the “analyse non-bond interactions” component^36^.

## Chemistry

**Scheme 1.**
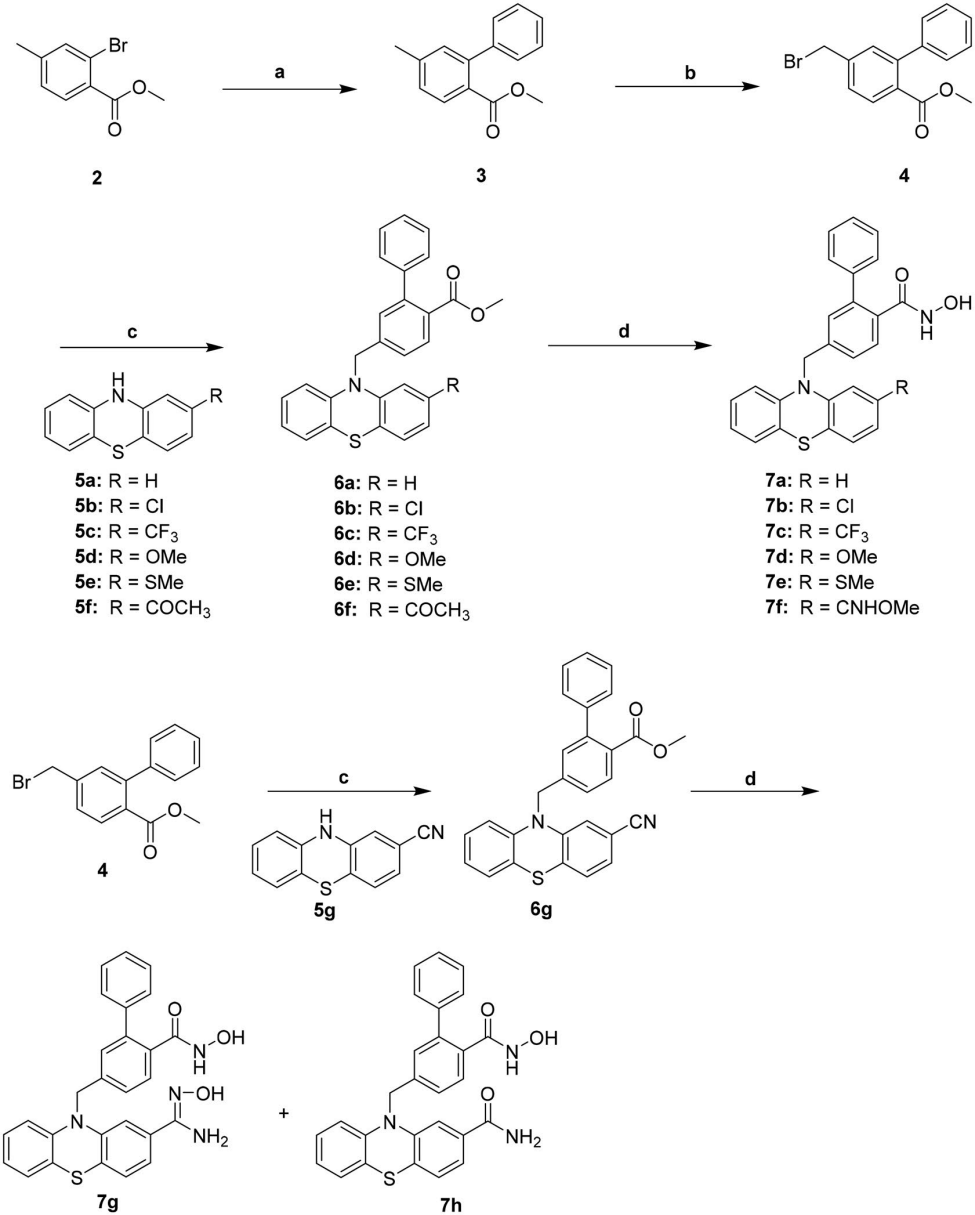
Reagents and conditions: (a) PhB(OH)_2_, Pd(OAc)_2_, Ph_3_P, K_2_CO_3_, Dioxane, H_2_O, Δ; (b) NBS, AIBN, MeCN, Δ; (c) NaH, DMF, RT; (d) 50%NH_2_OH_(aq)_, NaOH, MeOH, THF, RT.

Compounds **7a-h** were synthesised as described in Scheme 1. Suzuki coupling of compound **2** in the presence of Pd(OAc)_2_ and Ph_3_Ph gave **3**. Wohl-Ziegler bromination of compound **3** using NBS yielded **4**. Compound **4** reacted with substituted phenothiazines **5a-f** provided **6a-f**, respectively. Compounds **6a-f** was reacted with NH_2_OH to produce compounds **7a-f**, respectively. Reaction of compound **4** with cyano compound **5 g** generated **6 g**. Compound **6 g** was converted to **7 g** and **7h** using NH_2_OH.

**Scheme 2.**
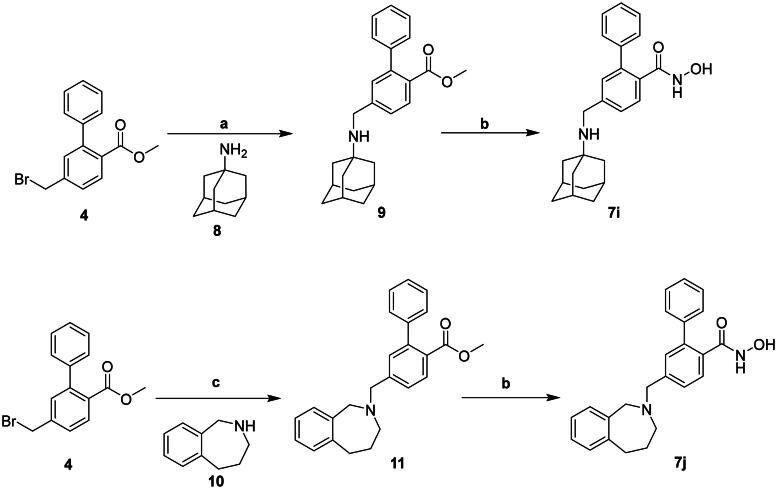
Reagents and conditions: (a) K_2_CO_3_, DMF, RT; (b) 50%NH_2_OH_(aq)_, NaOH, MeOH, THF, RT; (c) NaH, DMF, RT.

Compounds **7i-j** were synthesised as illustrated in Scheme 2. Compound **4** was reacted with amantadine in the presence of K_2_CO_3_ to give **9**. Reaction of compound **9** with NH_2_OH yielded **7i**. Compound **4** was reacted with benzoazepine **10** using NaH to produce **11**.

**Scheme 3.**
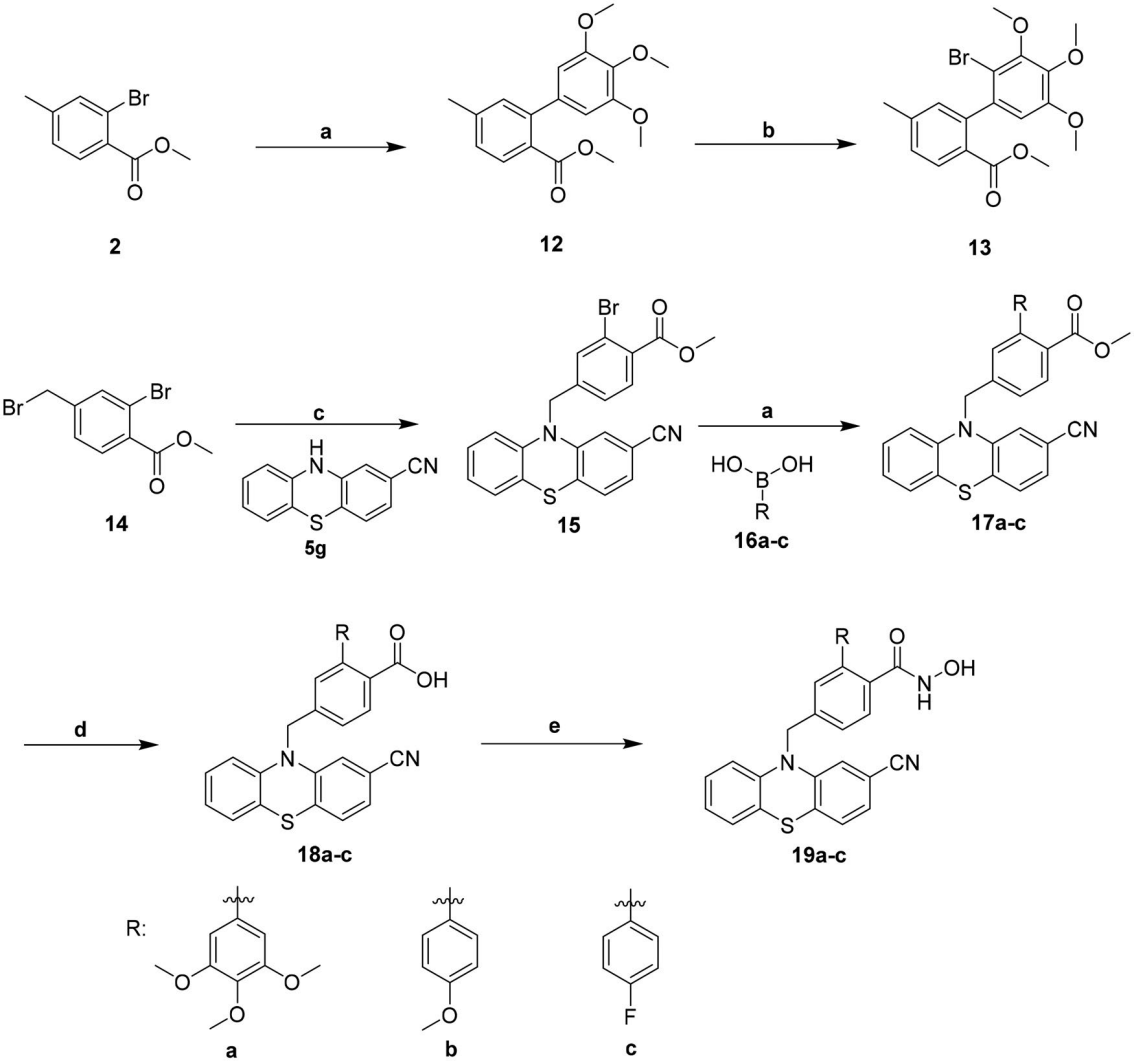
Reagents and conditions: (a) Pd(OAc)_2_, Ph_3_P, K_2_CO_3_, Dioxane, H_2_O, Δ; (b) NBS, AIBN, MeCN, Δ; (c) NaH, DMF, RT; (d) LiOH, THF, MeOH, H_2_O, 45 °C; (e) BOP, NH_2_OH, DIPEA, DMF, RT.

Compounds **19a-c** were synthesised as described in Scheme 3. We previously attempted to synthesise benzylbromides containing a substituted phenyl group at the *ortho* position, following the method used for the synthesis of *ortho*-phenylbenzyl bromide **4** (Scheme 1). Suzuki coupling of compound **2** with trimethoxyphenyl boronic acid gave compound **12**. However, Wohl-Ziegler bromination of compound **12** unexpectedly produced the phenyl brominated product **13** instead of the desired benzylic brominated product, resulting in compound **13** as an unintended outcome of this reaction. Alternatively, compounds **19a-c** were synthesised using compound **14** as the starting material (Scheme 3). Compound **14** was reacted with compound **5 g** to generate **15**. Suzuki coupling of compound **15** with phenylboronic acid with various substituents such as 3,4,5-trimethoxy, 4-methoxy, and 4-fluoro produced **16a-c**. Saponification of compounds **17a-c** using LiOH generated corresponding acids **18a-c**. It is noted that cyano group is invulnerable to nucleophilic attack by hydroxide. The reaction temperature that was kept under 45 °C can prevent the cyano group on thiazine from further reacting with LiOH. Compounds **18a-c** underwent amide coupling with NH_2_OH in the presence of BOP to yield **19a-c**, respectively. The Supplementary Material provides details of the synthesis, isolation, and characterisation of intermediates **3**, **4**, **6a-g**, **8a-b**, **9**, **11–13**, **15**, **17a-c**, and **18a-c** as well as the ^1^H- and ^13^C-NMR, and HRMS spectra of compounds **7a**-**j**, and **19a**-**c** (Figures S3–S41). These compounds were determined to be at least 95% pure using HPLC (Figures S42–S54) in the Supplementary Material.

## Results and discussion

### HDAC enzyme inhibitory activity

The enzyme-inhibiting activities of the synthesised compounds **7a-j** and **19a-c** were evaluated against HeLa nuclear HDACs, class IIa, and class IIb HDACs (Table 1). Vorinostat and the reported selective class IIa HDAC inhibitor MG013 were used as reference compounds here. In this study, the HeLa nuclear HDACs represent a mixture of class I HDACs 1, 2, and 3. The *ortho*-phenyl substituted phenylhydroxamic acids exhibited greater selectivity for class IIa HDAC compared to unsubstituted phenylhydroxamic acid **LH4f** (Table 1). The phenyl linker-containing compound **LH4f** exhibited strong class IIb HDAC6 inhibition, with an IC_50_ value as low as 5 nM. In contrast, the biphenyl linker-containing compounds **7a-j** and **19a-c** displayed only weak HDAC6-inhibiting activities, with IC_50_ values ranging from 0.75 to 18 µM. Notably, the HDAC6 inhibitory activity of compound **7h** was 150-fold less potent than that of its counterpart, compound **LH4f**. These results suggest that the introduction of the *ortho*-phenyl group into these phenothiazine-bearing phenylhydroxamic acids greatly decreases their binding affinities to HDAC6. The chloro-containing compound **7b** and trifluoromethyl-containing compound **7c** exhibited higher class IIa HDAC inhibition than the unsubstituted compound **7a**, which suggests halogen function groups favourably contribute to class IIa enzyme-inhibiting activity. Compound **7d,** which contains a methoxy group, displayed greater activity than compound **7a**. In comparison, compound **7e**, which contains the thiomethyl group, exhibited weaker activity for class IIa and class IIb HDACs. The acetoxime moiety-bearing compound **7f** displayed class IIa HDAC inhibition comparable to compound **7a**. The most potent class IIa HDAC was exhibited with compound **7 g**, which contains a hydroxyimidamide. Compared to MG013, this compound displayed higher inhibitory activities against HDACs 4 and 9, whereas it exhibited weaker HDAC7-inhibiting activity. In particular, its IC_50_ value against HDAC9 was 40 nM. Compound **7h**, which contains an amide group, displayed HDAC4- and HDAC7-inhibitory activity similar to compound **7 g**. Next, we synthesised amantadine-capped compound **7i** and benzoazepine-capped compound **7j** to explore whether structural modifications in the cap group can improve the selective inhibition of class IIa HDACs and enhance the drug-like properties of the compounds. The amantadine cap in compound **7i** was chosen based on its rigid, and cage-like structure^37^, which we hypothesised may enhance binding affinity and selectivity by interacting with the specific regions of HDAC surface, and also improve membrane permeability and metabolic stability. Additionally, the benzoazepine cap in compound **7j**, with greater conformational flexiblility compared to the phenothiazine ring^38^, was explored to see it can allow the compound to adapt to the dynamic nature of the HDAC binding site and effectively interact with specific aromatic and hydrophobic residues. However, compound **7i** demonstrated much lower inhibitory activity against class IIa and class IIb HDACs compared to compound **7a**, which suggests that the amantadine group negatively contributes to activity. Similarly, compound **7j** exhibited weaker class IIa HDAC inhibitory activity than compound **7a**. Finally, we synthesised phenylhydroxamic acids, compounds **19a-c**, which contain different substituted phenyl groups on the ortho position. These three compounds exhibited weak activity for class IIa HDAC enzymes compared to the *ortho-*unsubstituted phenyl compounds, which indicates a negative contribution by substituents on the ortho-phenyl ring. The original *ortho*-phenyl phenylhydroxamic acid **1** (Figure 3) was found to exhibit not only potent HDAC7 inhibition but also strong HDAC8-inhibiting activity (IC_50_ = 70 nM)^24^. In contrast, compound **7 g** displayed only moderate activity against HDAC8 with an IC_50_ value of 1.95 µM (Table S2), which suggests that the introduction of the phenothiazine ring on *ortho*-phenyl phenylhydroxamic acid possibly decreases the binding affinity to HDAC8. We further used surface plasmon resonance (SPR) technique to confirm the binding of compound **7 g** to HDAC4 protein. HDAC4 serves as a representative of class IIa HDACs. The SPR data suggests that compound **7 g** can bind to HDAC4 protein, as shown in Figure S1.

**Table 1.** The IC_50_ values^a^ of inhibitory activities of compounds **7a-j**, and **19a-c** against HDAC isoform enzymes.

Compound	HeLa nuclear HDACs (μM)	Class IIa (nM)			Class IIb (nM)
		HDAC4	HDAC7	HDAC9	HDAC6
**7a**	>40	480 ± 40	710 ± 10	420 ± 60	3200 ± 770
**7b**	>40	280 ± 30	230 ± 20	200 ± 10	2120 ± 370
**7c**	>40	280 ± 40	230 ± 50	270 ± 20	3190 ± 110
**7d**	>40	800 ± 10	710 ± 120	640 ± 80	3680 ± 410
**7e**	>40	480 ± 70	330 ± 10	270 ± 40	5910 ± 150
**7f**	>40	580 ± 70	410 ± 20	320 ± 70	1610 ± 90
**7g**	>40	180 ± 60	190 ± 40	40 ± 1	970 ± 50
**7h**	>40	200 ± 50	160 ± 10	110 ± 10	750 ± 60
**7i**	>40	18740 ± 2160	12890 ± 2140	23730 ± 4140	>40000
**7j**	>40	910 ± 70	840 ± 30	950 ± 50	18220 ± 20
**19a**	>40	>40000	17870 ± 920	18610 ± 960	7600 ± 360
**19b**	>40	6280 ± 130	4030 ± 160	5340 ± 400	4030 ± 250
**19c**	>40	1000 ± 30	540 ± 60	720 ± 30	4220 ± 180
**LH4f**	0.100 ± 0.02	600 ± 140	160 ± 10	590 ± 90	4.6 ± 0.4
**Vorinostat**	0.04 ± 0.002	30280 ± 5810	>40000	>40000	10 ± 1
**MG013**	>40	390 ± 100	110 ± 20	350 ± 19	>40

^a^The values were obtained from at least three independent experiments.

## Interaction analysis of compound 7 g

**Figure 4.**
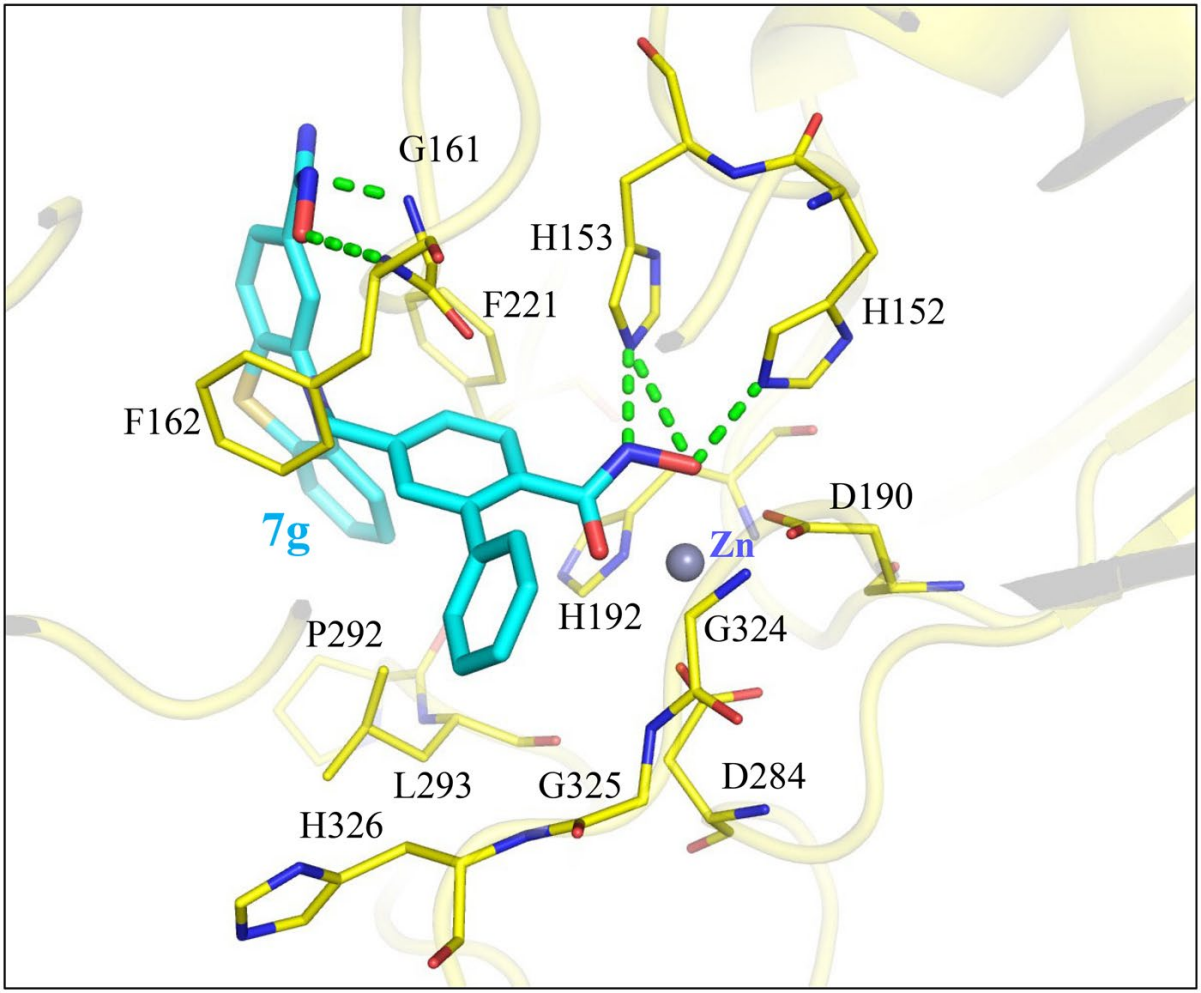
Docking pose of compound **7 g** in HDAC9 binding site. Compound **7 g** (blue) was molecularly docked into the HDAC9 binding site (yellow). Coordination of the zinc ion was observed, and additional interactions with binding site residues suggest favourable occupation of the HDAC9 binding site. Binding site residues are labelled and depicted as lines. Hydrogen bonds are denoted as green dashes.

As the most potent HDAC9 inhibitor, compound **7 g** was molecularly docked into the HDAC9 binding site to investigate its binding conformation. Compound **7 g** produces interaction patterns consistent with known HDAC inhibitors that correspond to the ZBG, linker, and cap groups (Figure 4). The phenylhydroxamic moiety functions as the ZBG. This moiety penetrates deeply into the HDAC9 binding site to occupy and coordinate the embedded zinc ion. Nitrogen and oxygen atoms located on the ZBG facilitate three hydrogen bonds, two with residue H153 and one with H152, respectively (Figure 4). The compound **7 g** linker contains an ortho-phenyl ring. Like many HDAC inhibitors, this linker spans a hydrophobic pocket between the zinc atom and the periphery of the binding site. The cap group occludes the HDAC9 binding site and generates hydrogen bonds to the nitrogen backbones of residues G161 and F162 (Figure 4). The hydrogen bonds may function as an anchor for the cap group, and additional hydrophobic interactions were found with residue F221. For example, compound **7h** exhibited weaker potency against HDAC9 compared to compound **7 g** (Table 1). The docking poses reveal that compound **7h**, unlike the more potent compound **7 g**, lacks a hydrogen bond to residue F162 at the cap group (Figure S2). Together, these interactions show that compound **7 g** can make sufficient interactions with the HDAC9 binding site.

### Structural analysis of HDACs suggests compound 7 g preference to HDAC9

**Figure 5.**
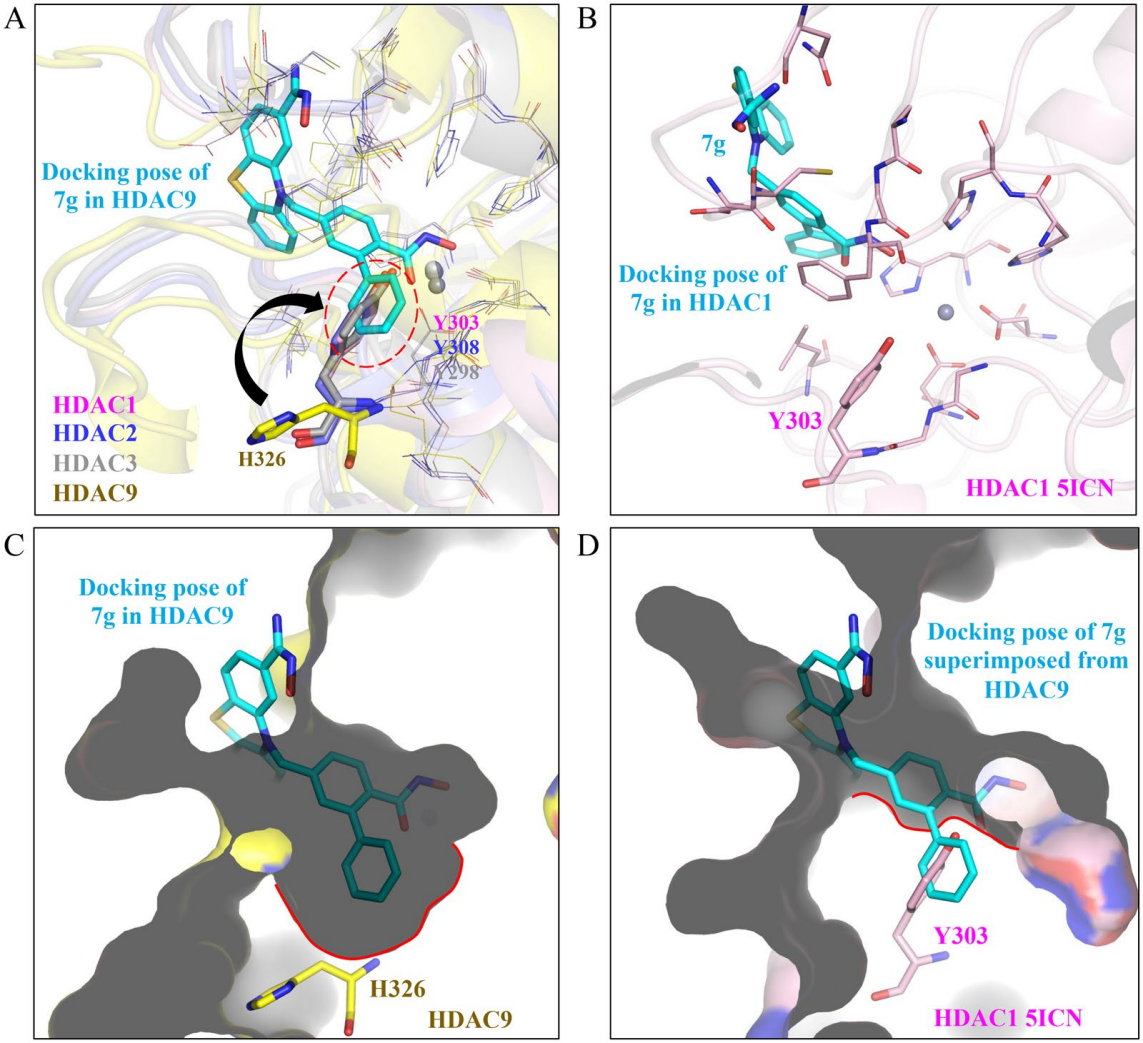
Structural analysis shows compound **7 g** preference for HDAC9. (A) Structures for HDACs 1, 2, 3, and 9 were superimposed. The docking pose of compound **7 g** (blue) in HDAC9 (yellow) was also included. A steric class was observed with the tyrosine residue, which is not present in HDAC9. (B) Docking compound **7 g** in HDAC1 (pink) gave an unconventional pose. Most notably, compound **7 g** did not occupy the HDAC1 binding site. (C) The binding pocket of HDAC9 can accommodate a larger aromatic ring. (D) In contrast, the tyrosine in HDAC1 reduces the binding pocket and sterically clashes with compound **7 g**.

The enzymatic assays revealed that compound **7 g** has a preference for inhibiting not only HDAC9 but also the Class IIa HDAC family (Table 1). HDACs are subdivided based on sequence homology and cellular localisation. The structural differences between the HDACs would better explain the inhibitory preference of compound **7 g**. The structures of HDAC1, HDAC2, HDAC3, and HDAC6 were superimposed with HDAC9 (Figure 5A). A tyrosine residue is present in the binding site of Class I HDACs and Class IIb, whereas HDAC9 contains a histidine (Figure 5A). Compound **7 g** presents a favourable docking pose, with the ZBG penetrating the binding site to occupy a pocket near the zinc ion in HDAC9. The *ortho*-phenyl ring on compound **7 g** is large but is accommodated by the side chain of residue H326 positioned away from the binding site (Figure 5B). In contrast, the tyrosine residue in the isozymes is pointing “inwards”. This could prevent the ZBG and hydrophobic linker of compound **7 g** from entering the binding site. The tyrosine residue of Class I HDACs severely restricts the binding site when compared to HDAC9 (Figure 5C). In particular, the *ortho*-phenyl ring would add steric bulkiness, thus reducing compound **7g**’s ability to penetrate and interact with the zinc ion in Class I HDACs 1, 2, 3 and in Class IIb HDAC6 (Figure 5C,D). Targeting the larger hydrophobic pocket in HDAC9 would be a reasonable target for increasing selectivity towards the HDAC9 isoforms. Together, the structural analysis details potential areas that impact the selectivity of compound **7 g** towards Class IIa HDACs.

### Compound 7 g inhibits growth in cancer cells

All of the synthesised compounds **7a-j** and **19a-c** were incubated with human oral cancer SCC2095 cells for 48 h and evaluated for their cytotoxicities (Table S1). Vorinostat and etoposide were tested as the reference compounds. The experimental results indicate that six of these compounds, **7d**-**e**, **7 g**-**h**, **19b,** and **19c,** exhibited potent cytotoxicities comparable to etoposide and vorinostat. In the series of compounds **7a**-**j**, except for compounds **7b** and **7c**, the cytotoxicities of these compounds were mostly consistent with their HDAC enzyme-inhibiting activities. Although compound **7 g** exhibited higher HDAC9 inhibition than compound **7h**, their cytotoxicities were equally potent. In contrast, compounds **19b** and **19c** exhibited strong cytotoxicities. However, their potency towards HDAC was not as strong as compounds **7 g** and **7h**, suggesting that their cytotoxicity may be due to off-target effects aside from the HDAC isozymes. These findings suggest that, in addition to HDAC inhibition, these two compounds likely affect other cell death-related events in the signalling pathway. To further examine the potential anti-proliferative effects of the most potent class IIa HDAC inhibitor **7 g**, it was also assayed for the cytotoxicities in human breast cancer MDA-MB-231, and gastric cancer SCM-1cells (Table 2). Compound **7 g** displayed favourable IC_50_ values at 3.0 ∼ 8.4 µM. Among these cancer cell lines, SCC2095 cells were more sensitive to compound **7 g** (IC_50_ = 3.0 ± 0.3 µM). The potency of compound **7 g** is on par with etoposide and tamoxifen in the SCC2095 and MDA-MB-231 cell lines, respectively. Furthermore, TMP269, a well-known class HDACIIa inhibitor^23^, also showed the inhibitory activity with an IC_50_ value of 18.1 ± 3.8 µM against SCC2095 cells (Table 2). However, compound **7 g** was not as potent as paclitaxel in the SCM-1 cell lines (Table 2). In order to investigate the toxicity towards normal cells, the cytotoxicity of compound **7 g** was evaluated in normal human lung fibroblast cells. Compound **7 g** demonstrated moderate cytotoxicity against normal cells, with an IC_50_ value of 8.0 µM, compared to its activity in SCC2095 cells. Nevertheless, these values show respectable potency in cell lines and indicate that compound **7 g** could be further studied in aberrant HDAC9 expressed diseases.

**Table 2.** IC_50_ values (μM) of cytotoxicities of compound **7 g** against human oral cancer SCC2095, breast cancer MDA-MB-231, and gastric cancer SCM-1 cells.

Compound	SCC2095	MDA-MB-231	SCM-1
**7g**	3.0 ± 0.3	8.4 ± 2.7	5.8 ± 0.3
**Etoposide**	3.3 ± 0.5	–	–
**Tamoxifen**	–	11.4 ± 0.9	–
**Paclitaxel**	–	–	0.08 ± 0.01
**TMP269**	18.1 ± 3.8	–	–

^a^The values were presented by at least three independent experiments. -: Not determined.

### Compound 7 g induces apoptosis in human oral cancer SCC2095 cells

**Figure 6.**
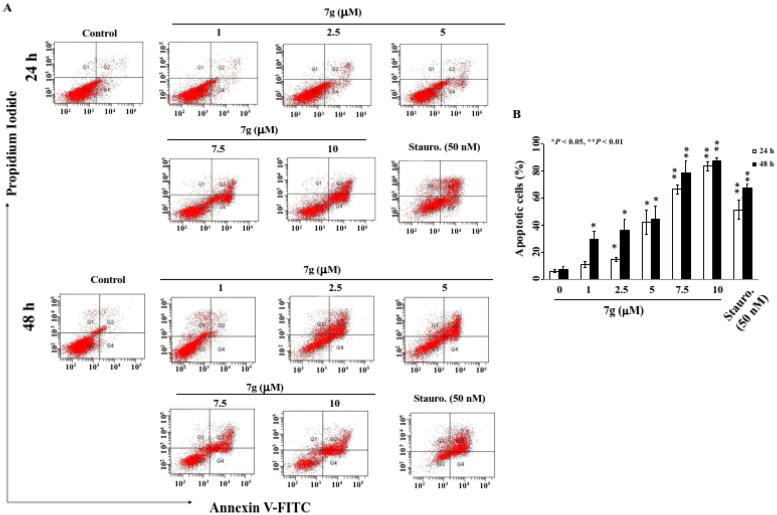
Annexin V-FITC/propidium iodide (PI) staining. (A) SCC2095 cells were treated with compound **7 g** (1 µM, 2.5 µM, 5 µM, 7.5 µM, and 10 µM) for 24 h or 48 h, followed by Annexin V-FITC/PY staining and flow cytometric analysis. 50 nM Staurosporine (Stauro.) as the positive control. (B) Statistically analysis of the percentage of apoptotic cells (Q2 + Q4). Data represent mean ± SD of three independent experiment. *P < 0.05, ** *P* < 0.01.

The potency of compound **7 g** suggests apoptosis was triggered. Indeed, HDAC inhibition is known to trigger the apoptotic signalling pathway^39^. To investigate how compound **7 g** triggers apoptosis, SCC2095 cells were double stained with PI/Annexin and then analysed using flow cytometry. Results showed induction of apoptosis in a dose and time-dependent manner (Figure 6A). Compared with the staurosporine control group, when compound **7 g** was administered at a concentration of 10 µM for 24 h, the percentage of apoptotic cells increased from 6.0% to 83.6% (Figure 6B). This phenomenon was observed in a dose-dependent manner with compound **7 g** tested at 1, 2.5 µM, 5 µM, 7.5 µM, and 10 µM for 48 h resulting in apoptosis rates of 29.2%, 35.9%, 44.4%, 78.2%, and 87.1%, respectively (Figure 6B). Together, not only is compound **7 g** potent in the tested cancer cells, but treatment with compound **7 g** also triggers apoptosis.

### Compound 7 g modulates caspase- and class II HDAC- related proteins in human oral cancer SCC2095 cells

**Figure 7.**
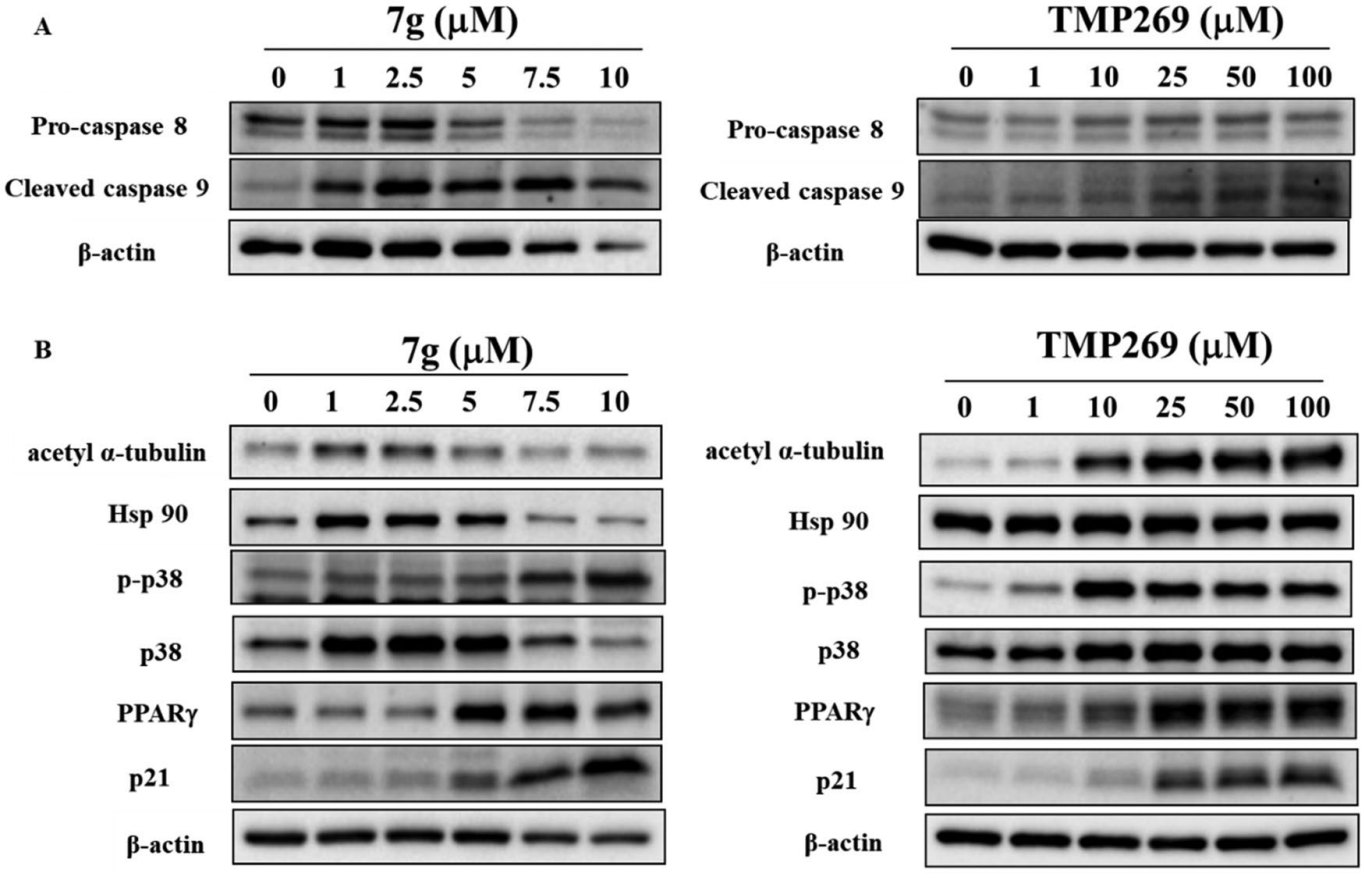
Effects of compound **7 g** and TMP269 on (A) pro-caspase 8, cleaved caspase 9, and (B) acetyl α-tubulin, Hsp90, p-p38, p38, PPARγ, and p21 in SCC2095 cells. Cells were treated with compound **7 g** and TMP269 at the indicated concentrations for 24 h, and cell lysates were immunoblotted. (*n* = 3).

Apoptotic related protein expression in SCC2095 cells was also modulated when treated with compound **7 g**. As shown in Figure 7(A), compound **7 g** down-regulated the levels of pro-caspase 8 and up-regulated the levels of cleaved caspase 9. We also found that TMP269 showed the similar trend on the expression of procaspase 8 and cleaved caspase 9 (Figure 7A). Studies indicate that HDAC4 can promote cell proliferation and invasion, which is associated with the repression of p21, a cyclin-dependent kinase (CDK) inhibitor, in various cancer cells^40–44^. Additionally, HDAC9 is found to suppress the expression of PPARγ, a nuclear transcription factor^45–47^. We observed that the levels of HDAC6 substrates such as acetyl-α-tubulin and Hsp90, and two class IIa HDAC-related proteins including PPARγand p21 were increased in cells treated with compound **7 g** (Figure 7B). The results also revealed that TMP269 ­modulated the levels of the biomarkers of class IIa HDACs (Figure 7B). Compound **7 g** exhibited greater cytotoxicity in comparison to TMP269 (Table 2). The decreased expression of acetyl-α-tubulin may be due to the greater cytotoxicities of compound **7 g**, which could reduce overall protein levels due to cell death. This demonstrated that compound **7 g** had an inhibitory impact on both the cellular and protein levels by targeting IIa and IIb HDAC in SCC2095 cells. Consistent with previous studies^48^^,^^49^, HDAC6 inhibition increases acetylation of α-tubulin. Previous studies reported that chronic inflammation contributes to the carcinogenesis of oral cancer, and the inhibition of HDAC6 led to increased levels of p-p38 in LPS-treated macrophages^50^^,^^51^. Similarly, our results demonstrated that **7 g** up-regulated the phosphorylation of p38 in a dose-dependent manner in SCC2095 cells. Accordingly, these findings confirmed that compound **7 g** exhibited class II HDAC inhibitory activity in cells.

### Compound 7 g increases reactive oxygen species (ROS) production

**Figure 8.**
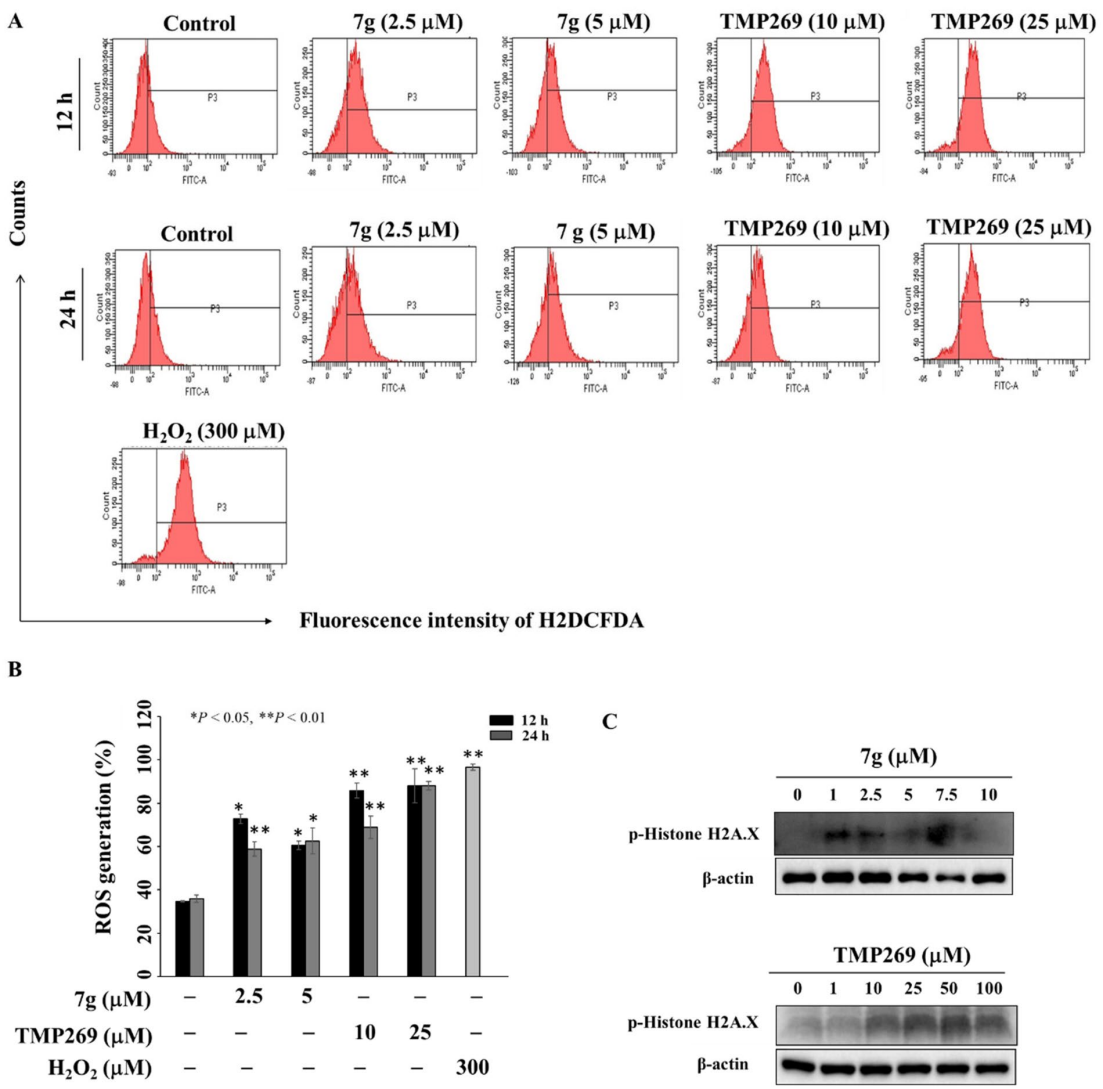
Compound **7 g** and TMP269 increased ROS generation in SCC2095 cells. (A) Cells were treated with DMSO or compound **7 g** or TMP269 for 12 h or 24 h and stained with H_2_DCFDA probe for another 30 min. Then, cells were assessed by flow cytometry. (*n* = 3) (B) Statistically analysis of cells with staining with the ROS probe. Data represent mean ± SD of three independent experiment. *P < 0.05, ** *P* < 0.01. (C). Compound **7 g** and TMP269 increases the phosphorylation expression of Histone H2A.X.

Aberrant ROS generation contributes to oral cancer progression and resistance of chemotherapeutic agents^52^^,^^53^. Tavareas et al. reported that tubastatin A induces apoptosis and ROS accumulation in cisplatin-resistant oral cancer cells^54^. Flow cytometric analysis showed that the ROS generation was increased after the treatment of compound **7 g** or TMP269 for 12 h or 24 h (Figure 8A,B). Western blotting revealed that the treatment of compound **7 g** or TMP269 increased the phosphorylation of Histone H2A.X, a DNA damage biomarker^55^, in SCC2095 cells (Figure 8C). These data suggested that compound **7 g** increased ROS production accompanied by the increased DNA damage.

## Conclusion

In conclusion, this study synthesised a novel series of *ortho*-phenyl phenylhydroxamic acids (**7a-i**, and **19a-c**) using **LH4f** as the lead compound. **LH4f** is a pan-HDAC inhibitor, showing potent inhibitory activity towards class I, IIa, and IIb HDACs. When compared to compound **LH4f**, most of these newly synthesised compounds displayed significantly improved class IIa HDAC selectivity. Notably, compound **7 g** demonstrated the highest inhibiting activity against class IIa HDACs with IC_50_ values of 40–180 nM. Additionally, compound **7 g** proved cytotoxic against several human cancer cell lines. Further analysis showed that compound **7 g** not only induced caspase-dependent apoptosis, but can also modulate the phosphorylation of p38 in the oral cancer cell line SCC2095. Treatment with compound **7 g** shows an increase in ROS production as well as DNA damage, further triggering apoptosis in the cancer cell line. The resulting compounds in this study serve as interesting candidates for developing class IIa HDAC inhibitors for potential therapeutic use.

## Supplementary Material

Original Image for Fig 5A Pro caspase 8 TMP269.tif

Original Image for Fig5A actin TMP269.tif

Original Image for Fig 5B betaactin 7g.tif

Original Image for Fig 5B p38 TMP269.tif

Original Image for Fig 6C beta actin 7g 0626.tiff

Original Image for Fig 6C phosphoHistone H2AX TMP269.tif

Original Image for Fig 5B Hsp90 7g.tif

Original Image for Fig 5B phosphop38 7g.tif

Original Image for Fig 5B PPARr TMP269.tif

Original Image for Fig 5A betaactin 7g.tif

Original Image for Fig 5B Actin TMP269.tif

Original Image for Fig 5A Cleaved caspase 9 7g.tif

Original Image for Fig 6C p Histone H2AX 7g 0626.tiff

Original Image for Fig 5B p21 TMP269.tif

Original Image for Fig 5B acetylalphatubulin 7g.tif

Original Image for Fig5A Cleaved caspase 9 TMP269.tif

Original Image for Fig 5B p21 7g.tif

Original Image for Fig 5B p38 7g.tif

Original Image for Fig 5A Pro caspase 8 7g.tif

Original Image for Fig 5B acetylalphatubulin TMP269.tif

Original Image for Fig 5B PPARr 7g.tif

Original Image for Fig 5B pp38 TMP269.tif

Original Image for Fig 5B Hsp90 TMP269.tif

supporting information 20240819.docx

Original Image for Fig 6C beta actin TMP269.tif

## Data Availability

The data that support the findings of this study are available from the corresponding author, WJH, upon reasonable request.
