# Supplementary figures and images for: Synthesis and biological evaluation of ortho-phenyl phenylhydroxamic acids containing phenothiazine with improved selectivity for class IIa histone deacetylases
Source: J Enzyme Inhib Med Chem. 2024 Sep 24;39(1):2406025. doi: 10.1080/14756366.2024.2406025 (PMC11423540; doi:10.1080/14756366.2024.2406025)

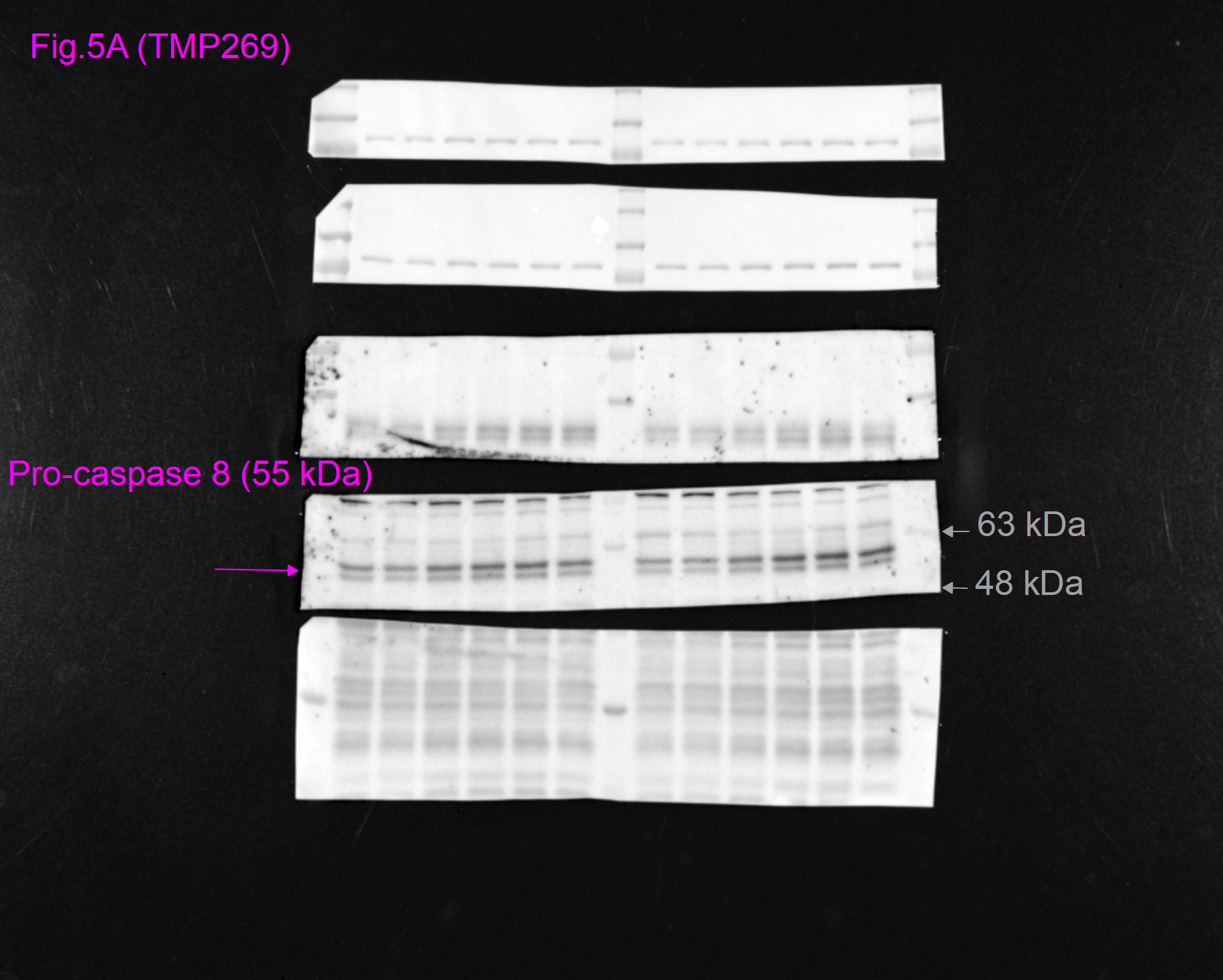

Supplement: Original Image for Fig 5A Pro caspase 8 TMP269.tif [file IENZ_A_2406025_SM9278.tif]

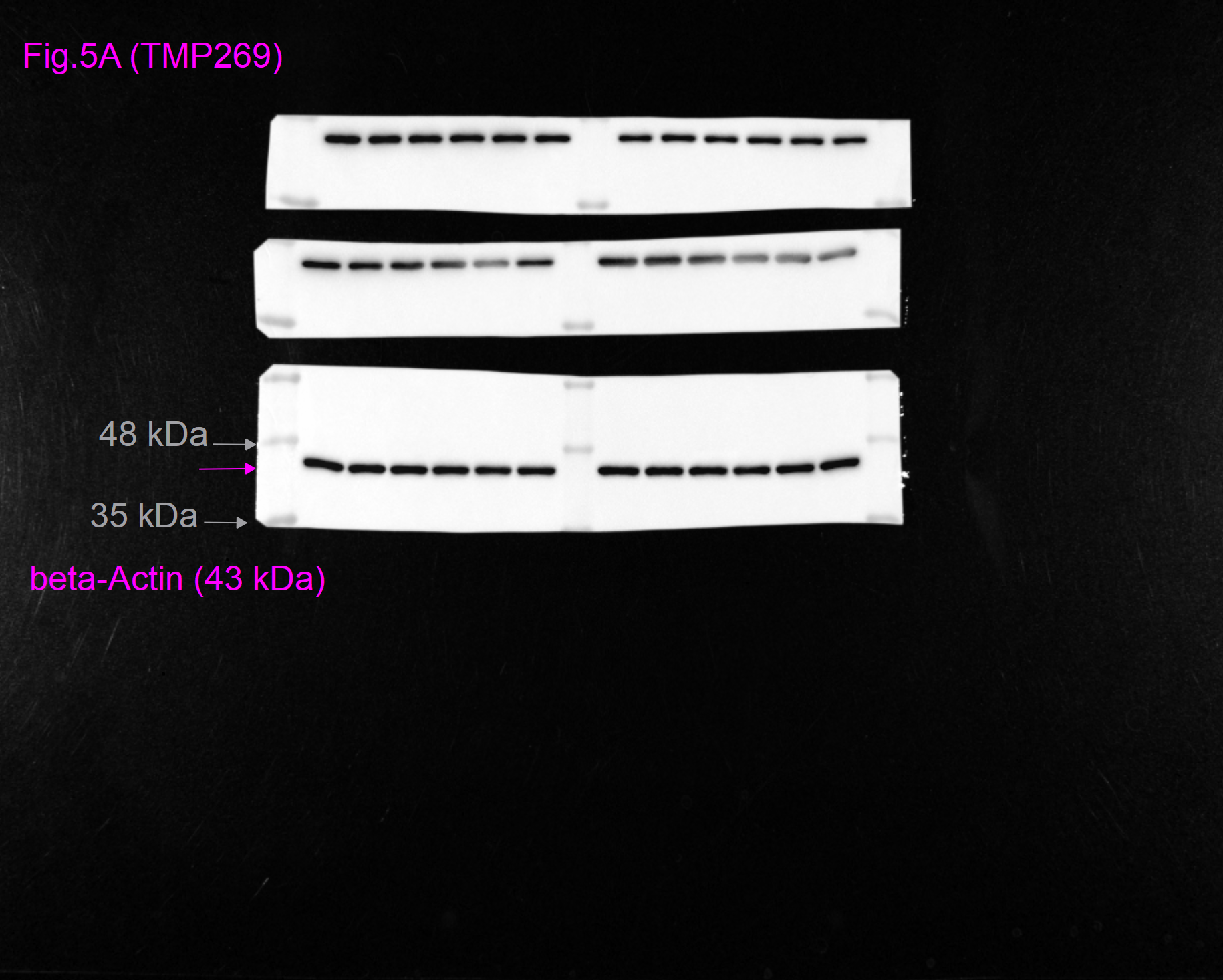

Supplement: Original Image for Fig5A actin TMP269.tif [file IENZ_A_2406025_SM9277.tif]

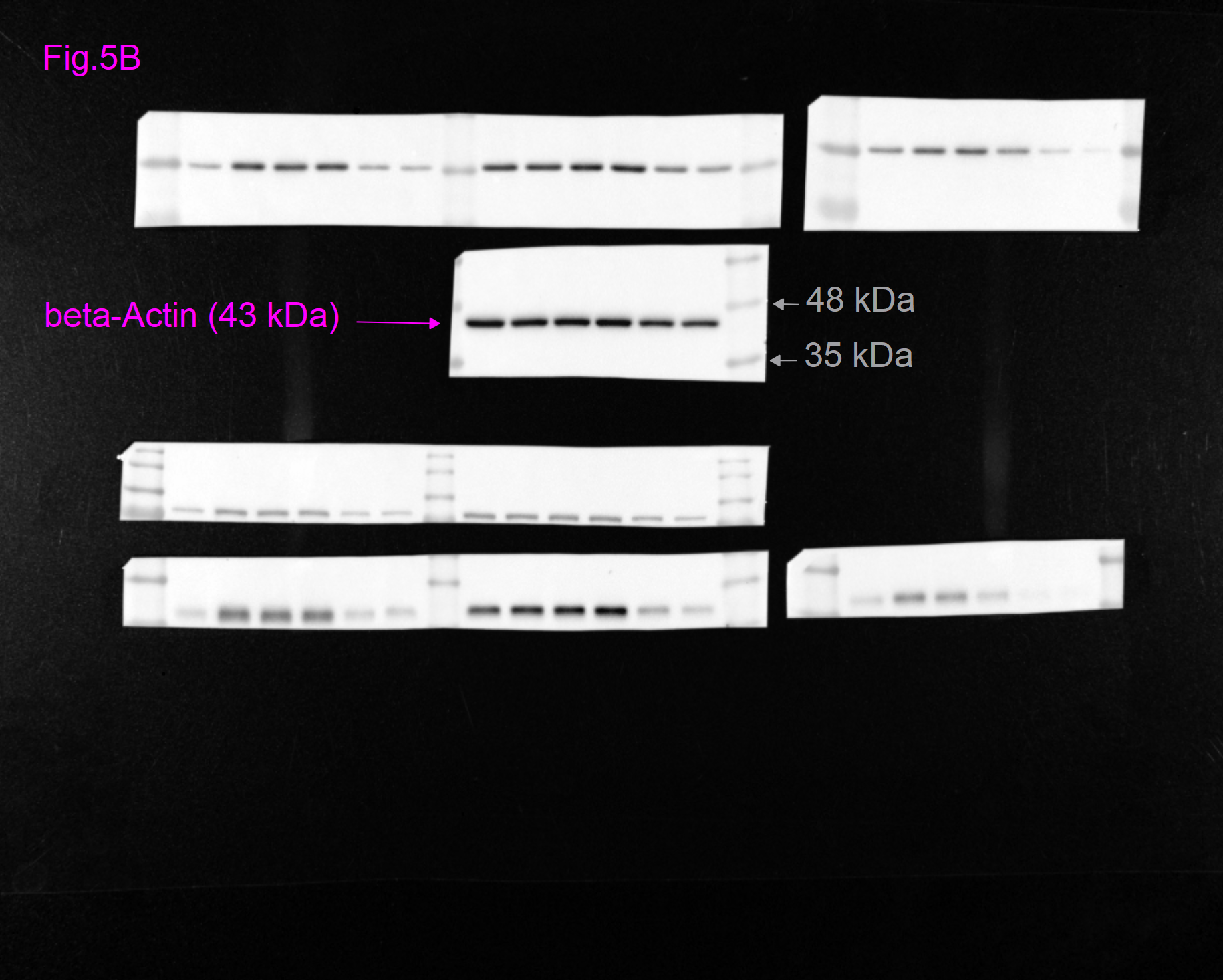

Supplement: Original Image for Fig 5B betaactin 7g.tif [file IENZ_A_2406025_SM9276.tif]

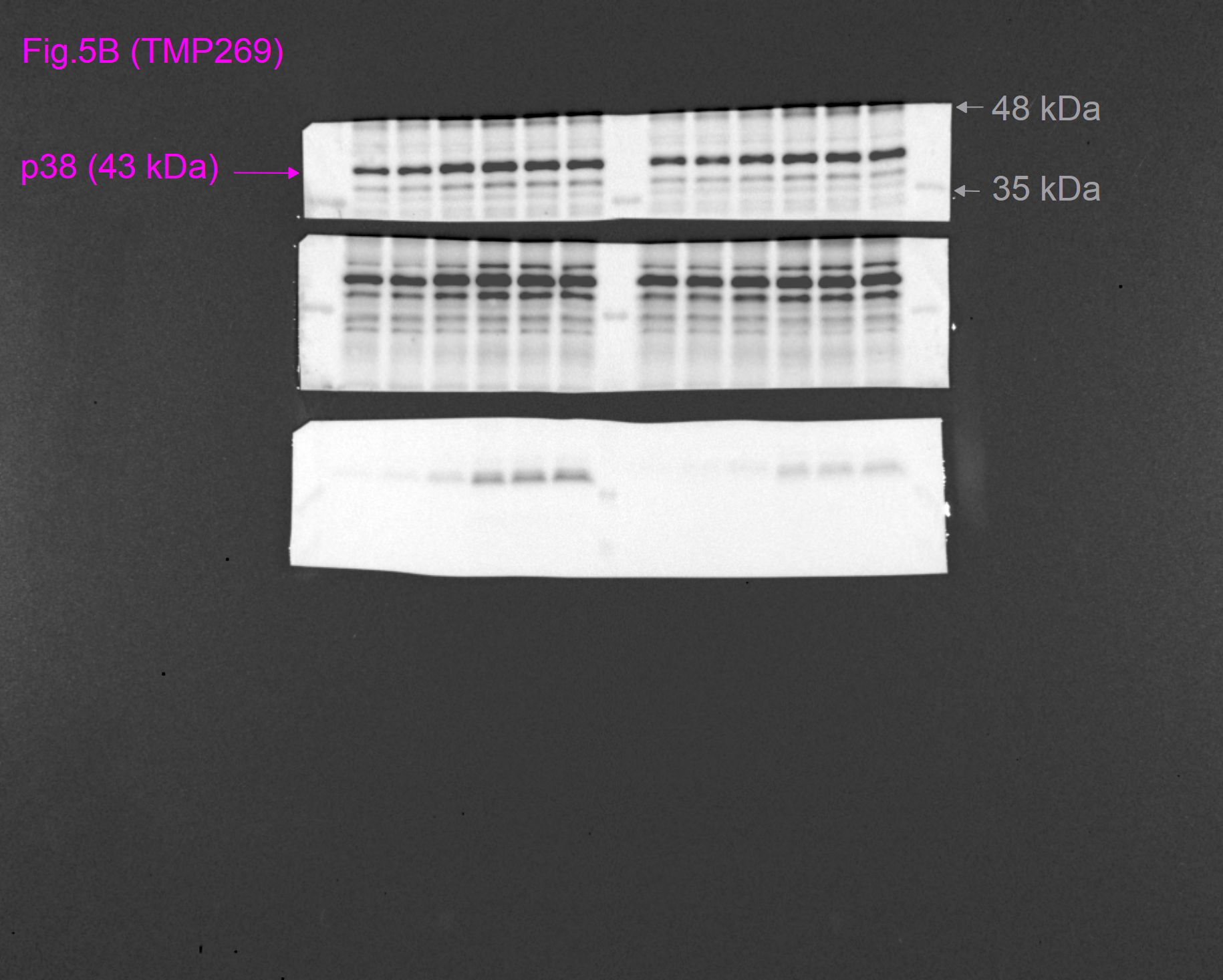

Supplement: Original Image for Fig 5B p38 TMP269.tif [file IENZ_A_2406025_SM9275.tif]

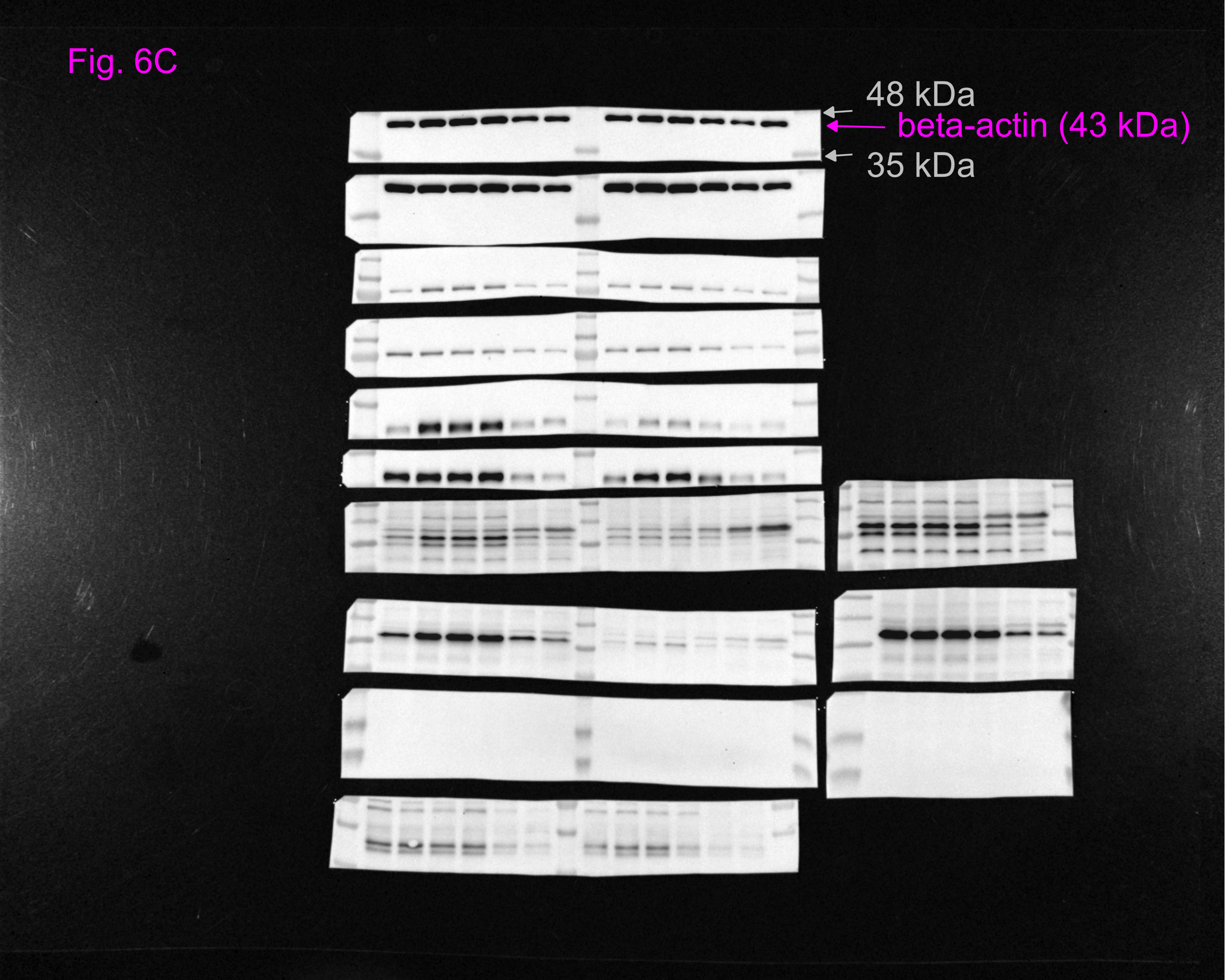

Supplement: Original Image for Fig 6C beta actin 7g 0626.tiff [file IENZ_A_2406025_SM9274.tiff]

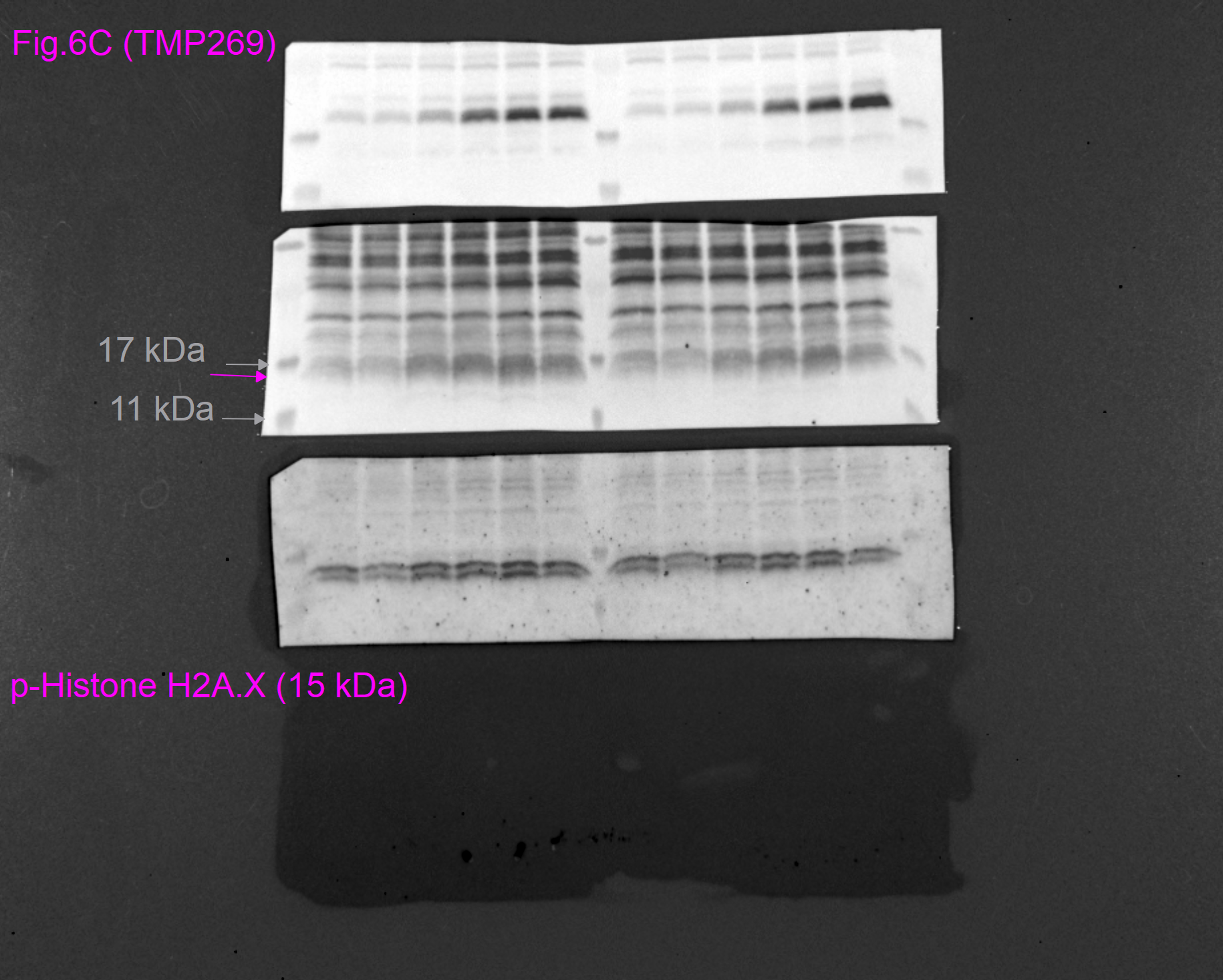

Supplement: Original Image for Fig 6C phosphoHistone H2AX TMP269.tif [file IENZ_A_2406025_SM9273.tif]

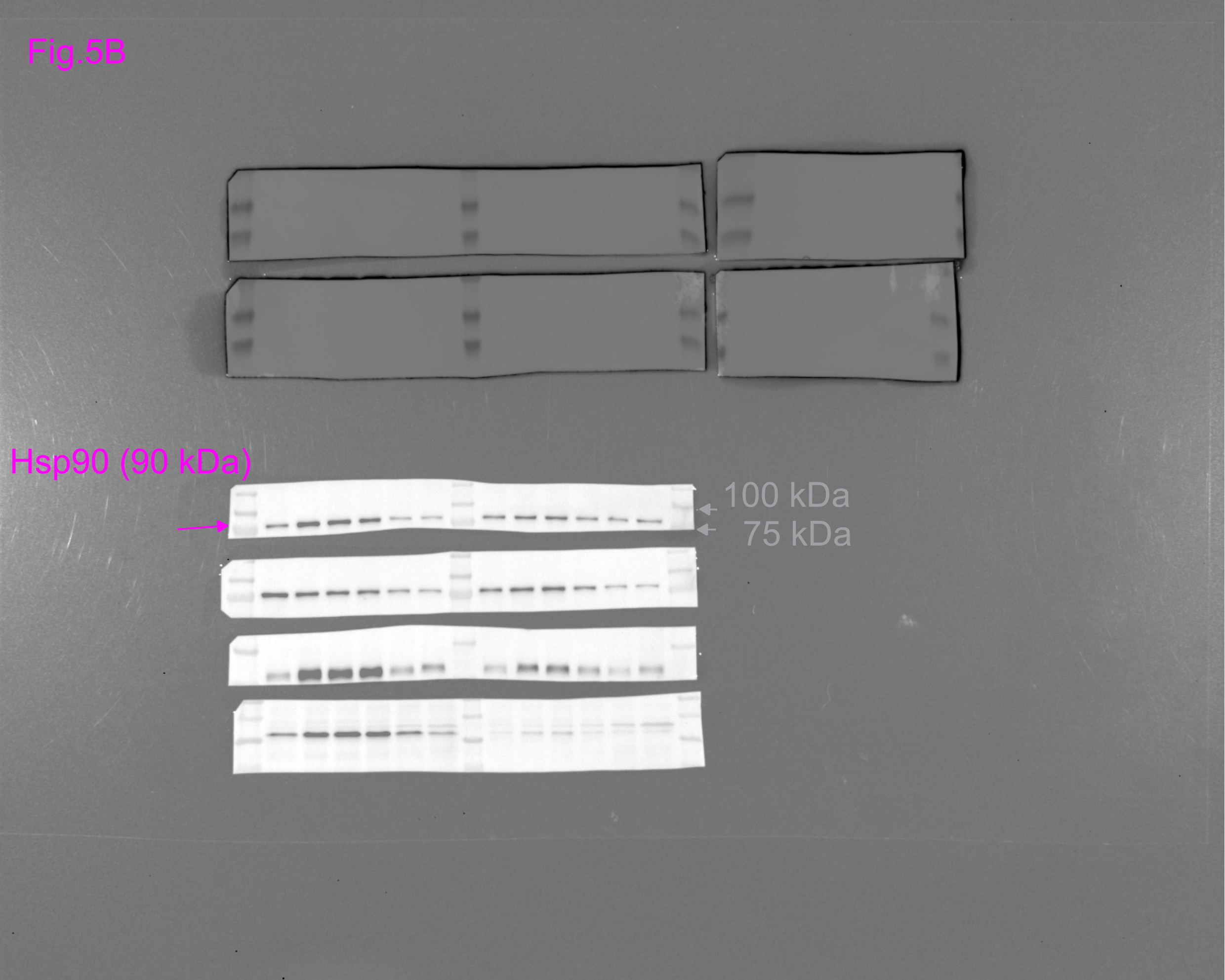

Supplement: Original Image for Fig 5B Hsp90 7g.tif [file IENZ_A_2406025_SM9272.tif]

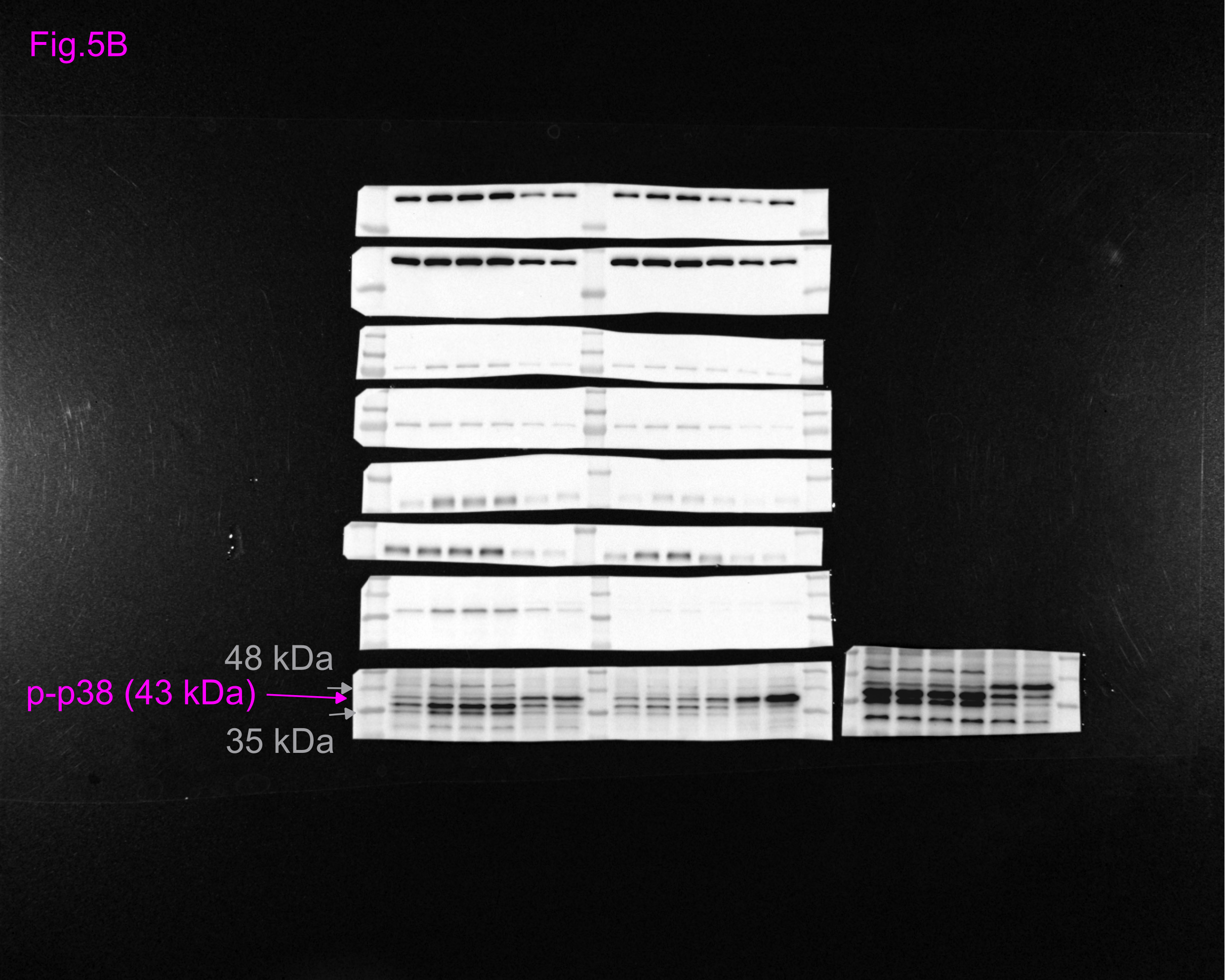

Supplement: Original Image for Fig 5B phosphop38 7g.tif [file IENZ_A_2406025_SM9271.tif]

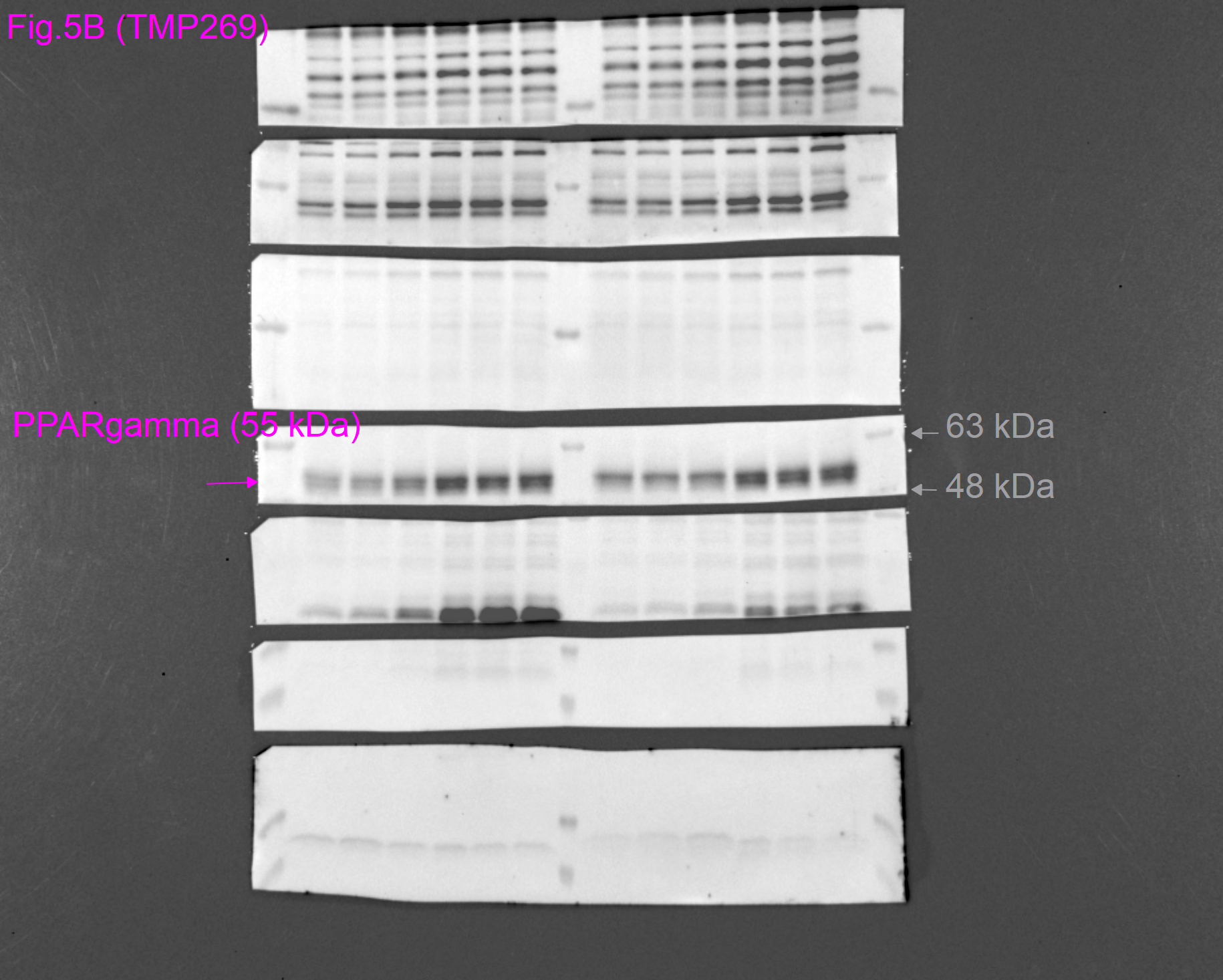

Supplement: Original Image for Fig 5B PPARr TMP269.tif [file IENZ_A_2406025_SM9270.tif]

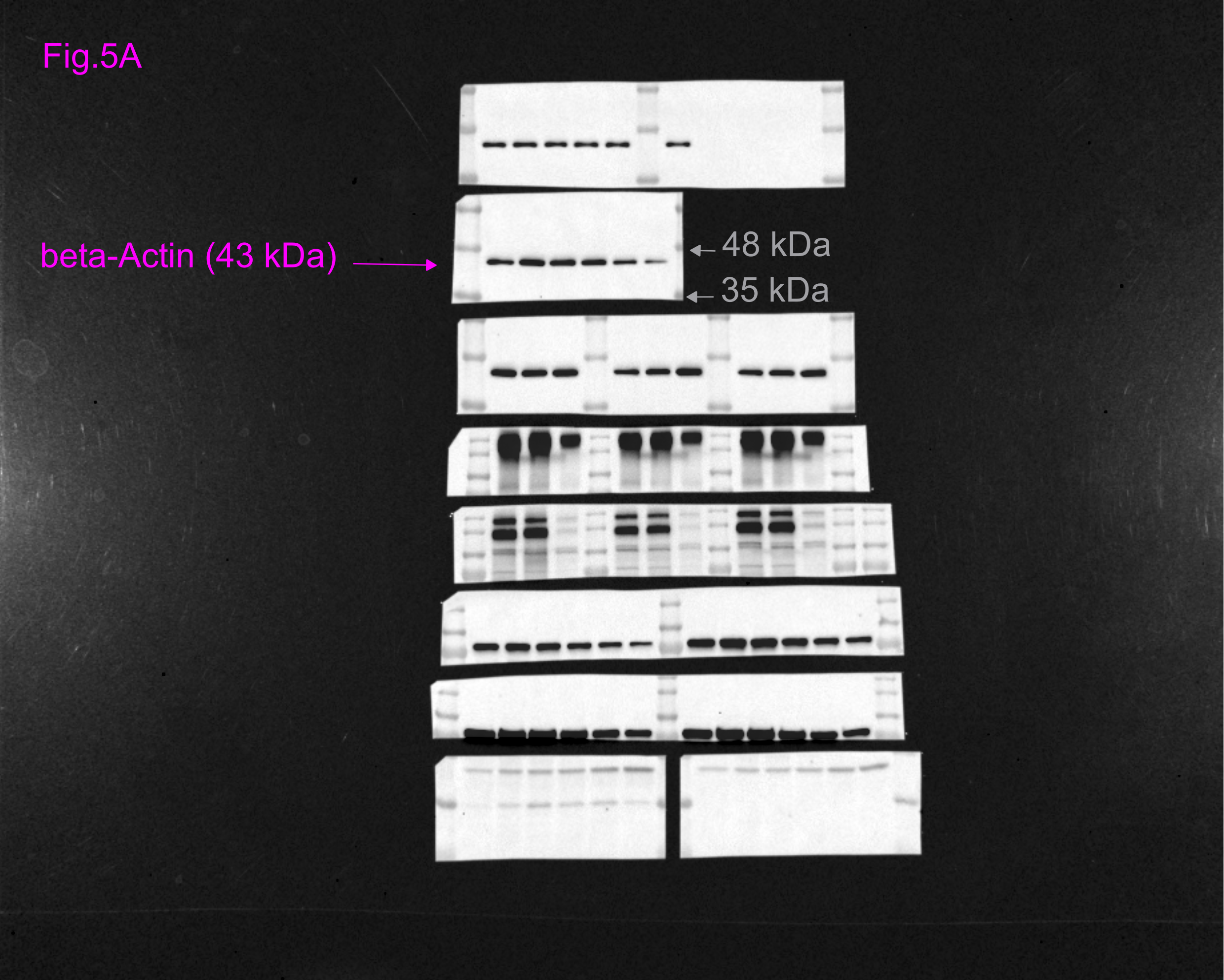

Supplement: Original Image for Fig 5A betaactin 7g.tif [file IENZ_A_2406025_SM9269.tif]

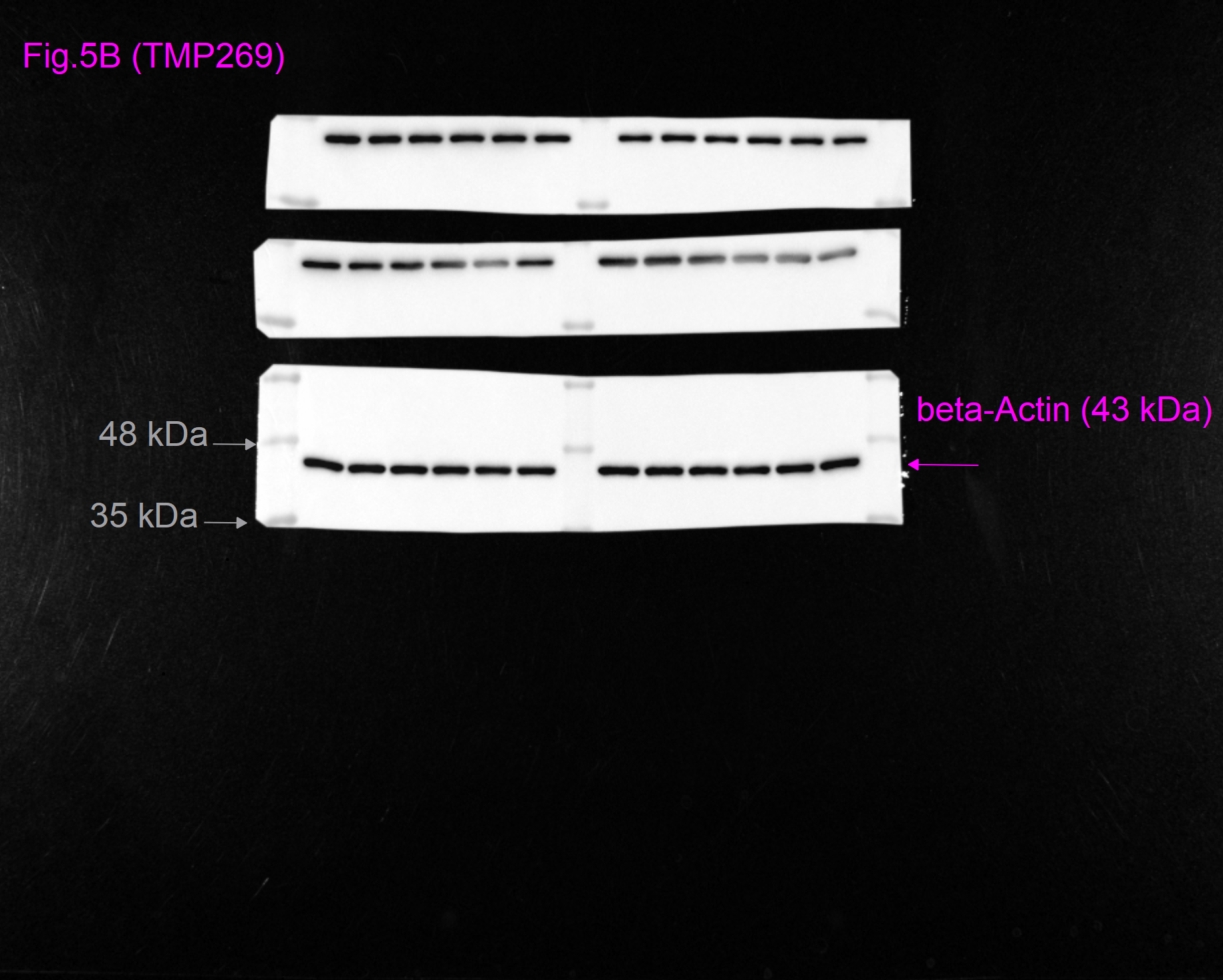

Supplement: Original Image for Fig 5B Actin TMP269.tif [file IENZ_A_2406025_SM9268.tif]

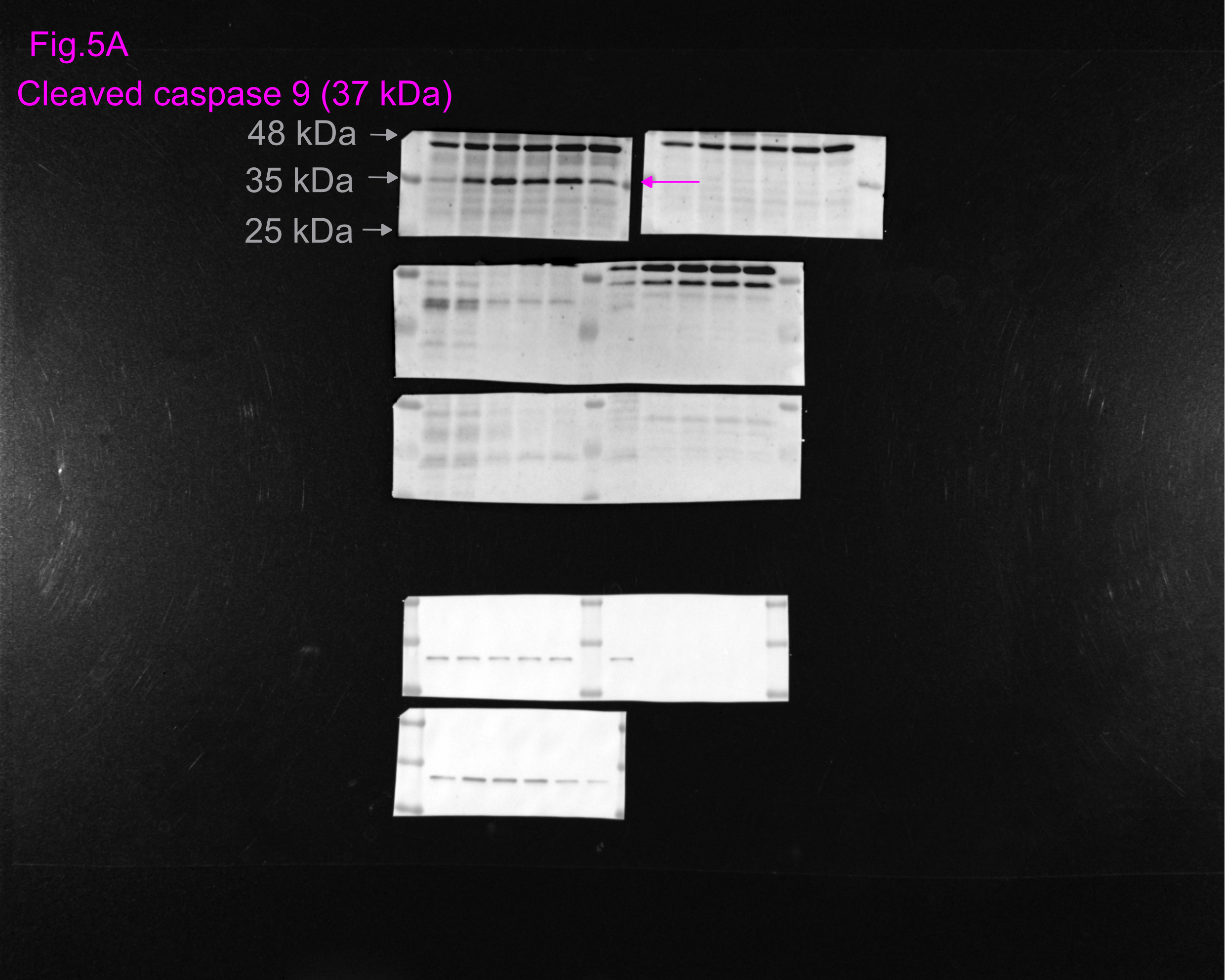

Supplement: Original Image for Fig 5A Cleaved caspase 9 7g.tif [file IENZ_A_2406025_SM9267.tif]

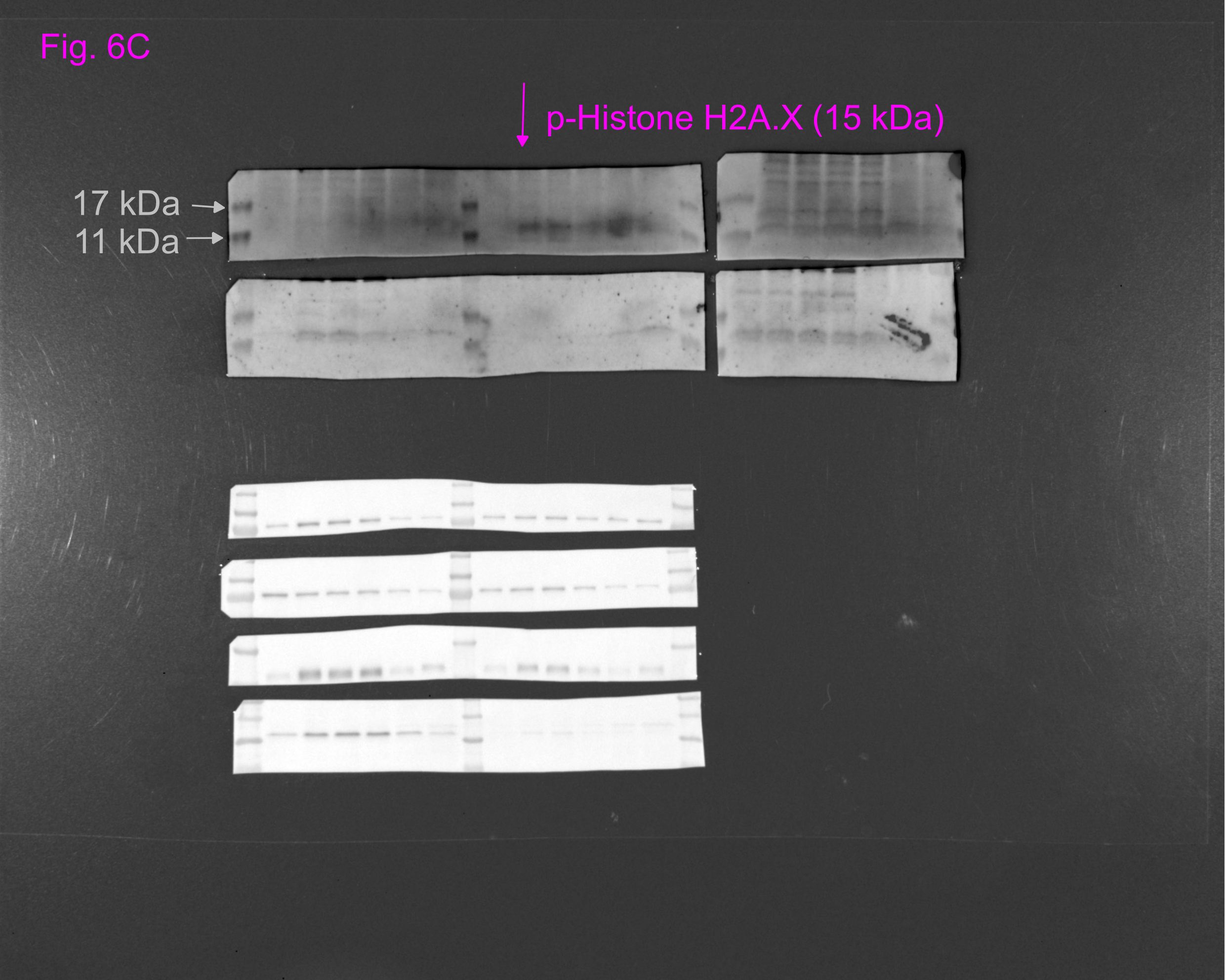

Supplement: Original Image for Fig 6C p Histone H2AX 7g 0626.tiff [file IENZ_A_2406025_SM9266.tiff]

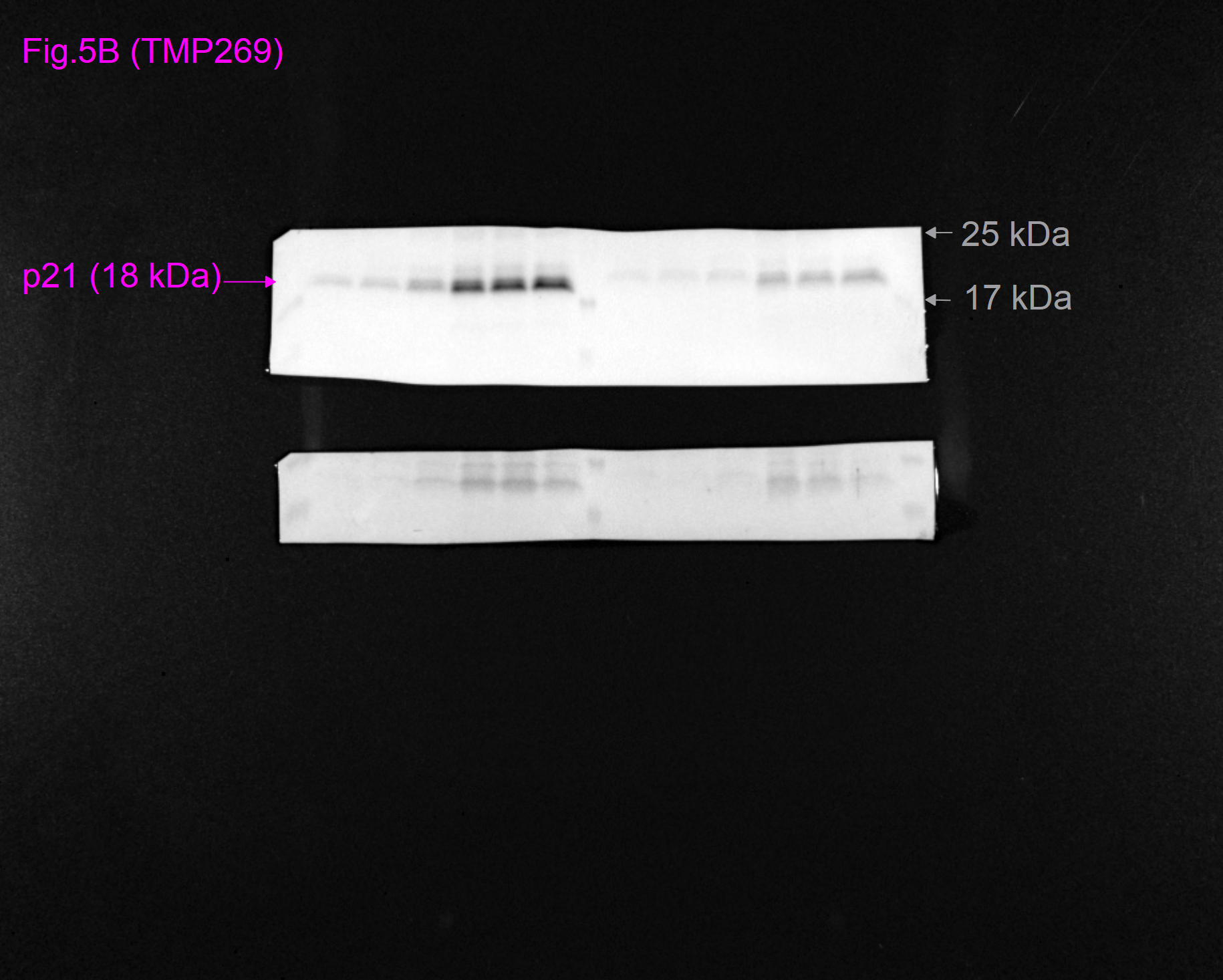

Supplement: Original Image for Fig 5B p21 TMP269.tif [file IENZ_A_2406025_SM9265.tif]

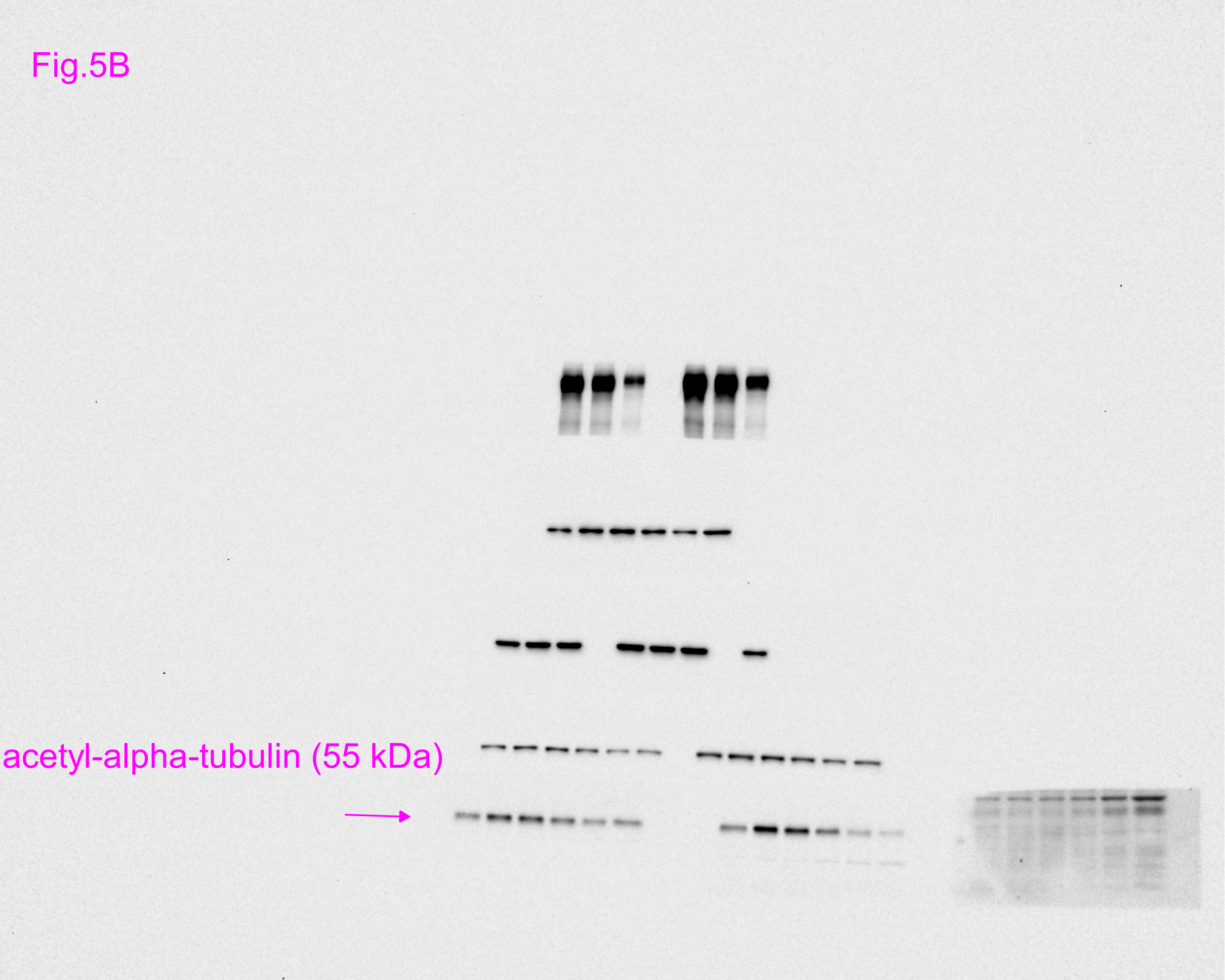

Supplement: Original Image for Fig 5B acetylalphatubulin 7g.tif [file IENZ_A_2406025_SM9264.tif]

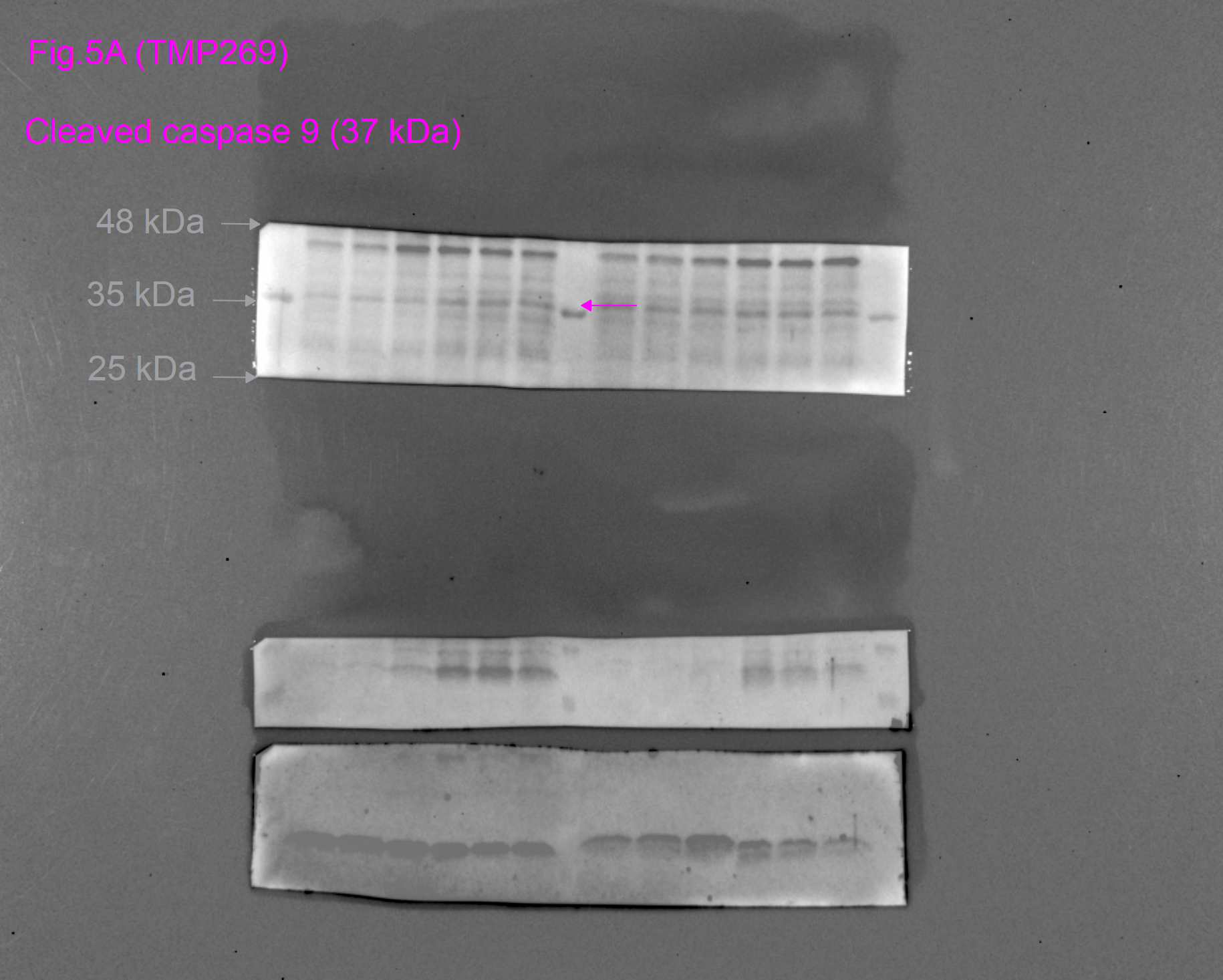

Supplement: Original Image for Fig5A Cleaved caspase 9 TMP269.tif [file IENZ_A_2406025_SM9263.tif]

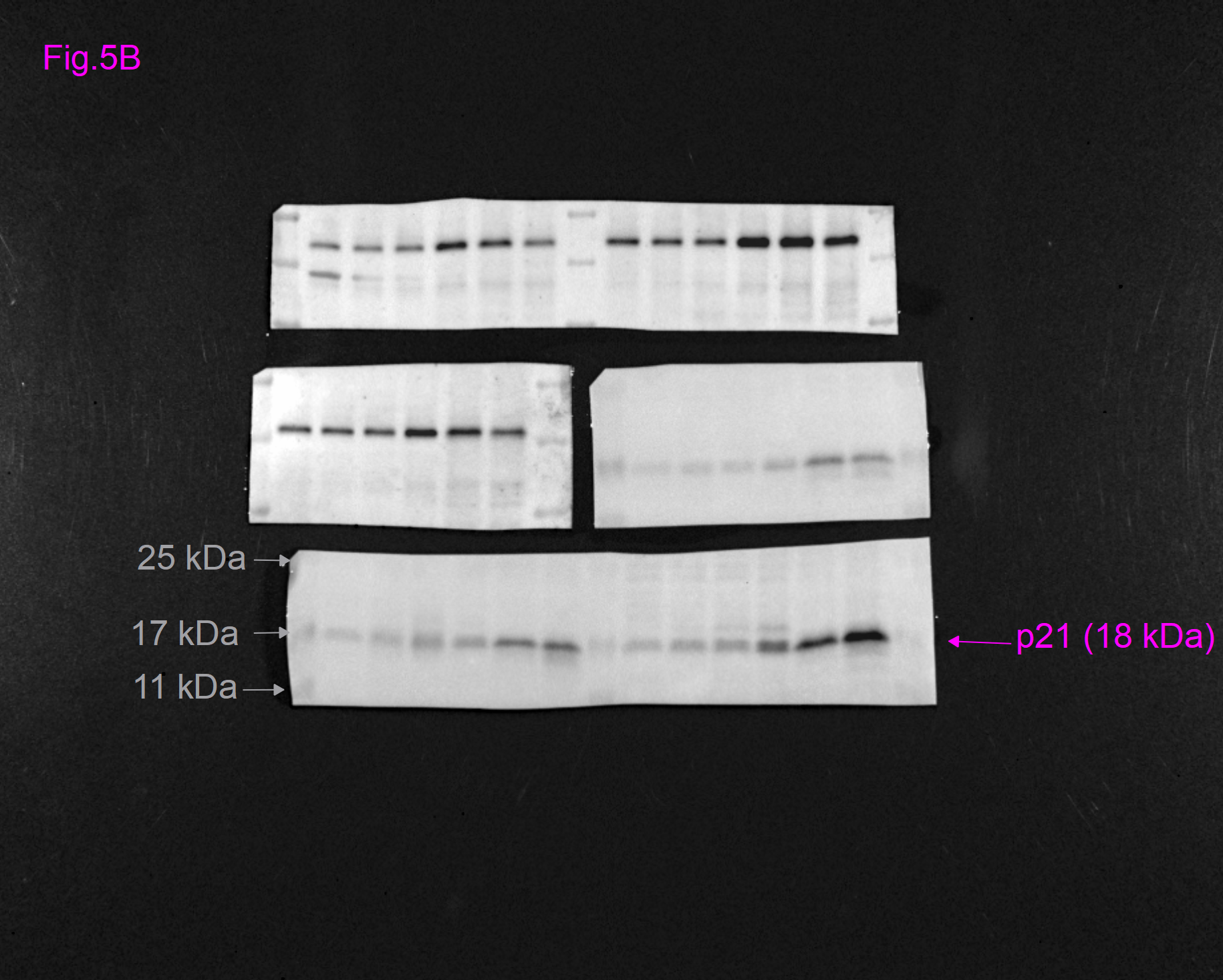

Supplement: Original Image for Fig 5B p21 7g.tif [file IENZ_A_2406025_SM9262.tif]

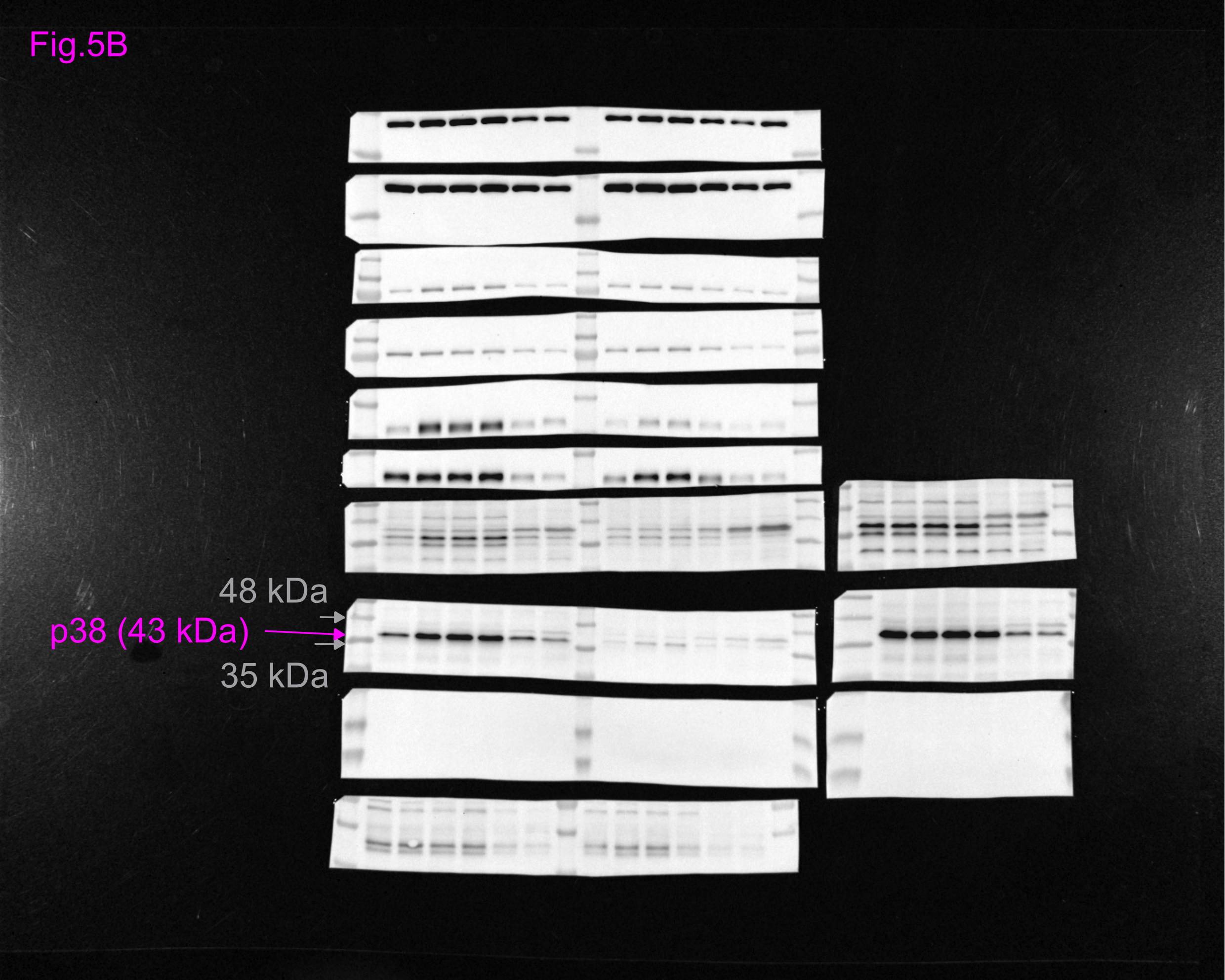

Supplement: Original Image for Fig 5B p38 7g.tif [file IENZ_A_2406025_SM9261.tif]

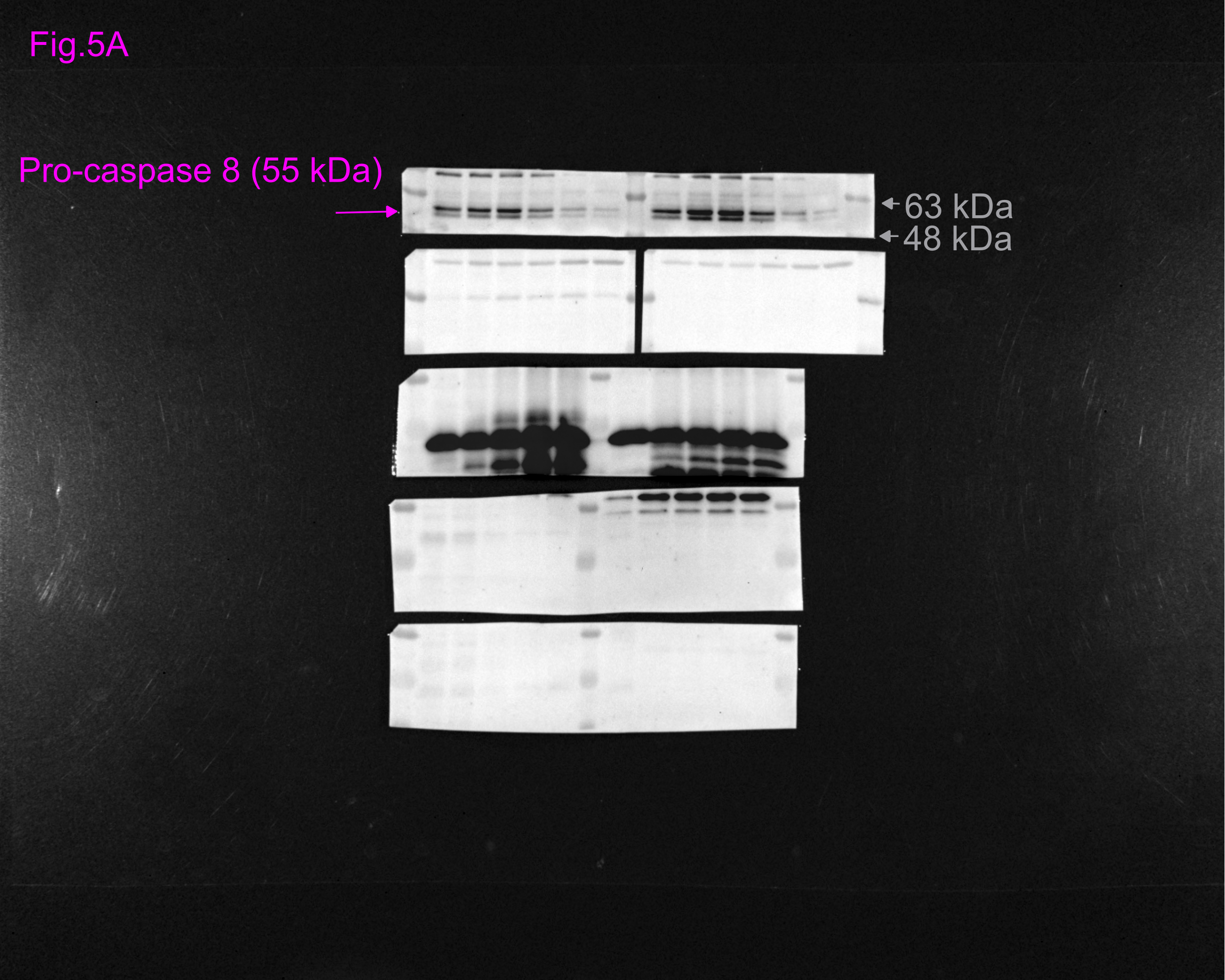

Supplement: Original Image for Fig 5A Pro caspase 8 7g.tif [file IENZ_A_2406025_SM9260.tif]

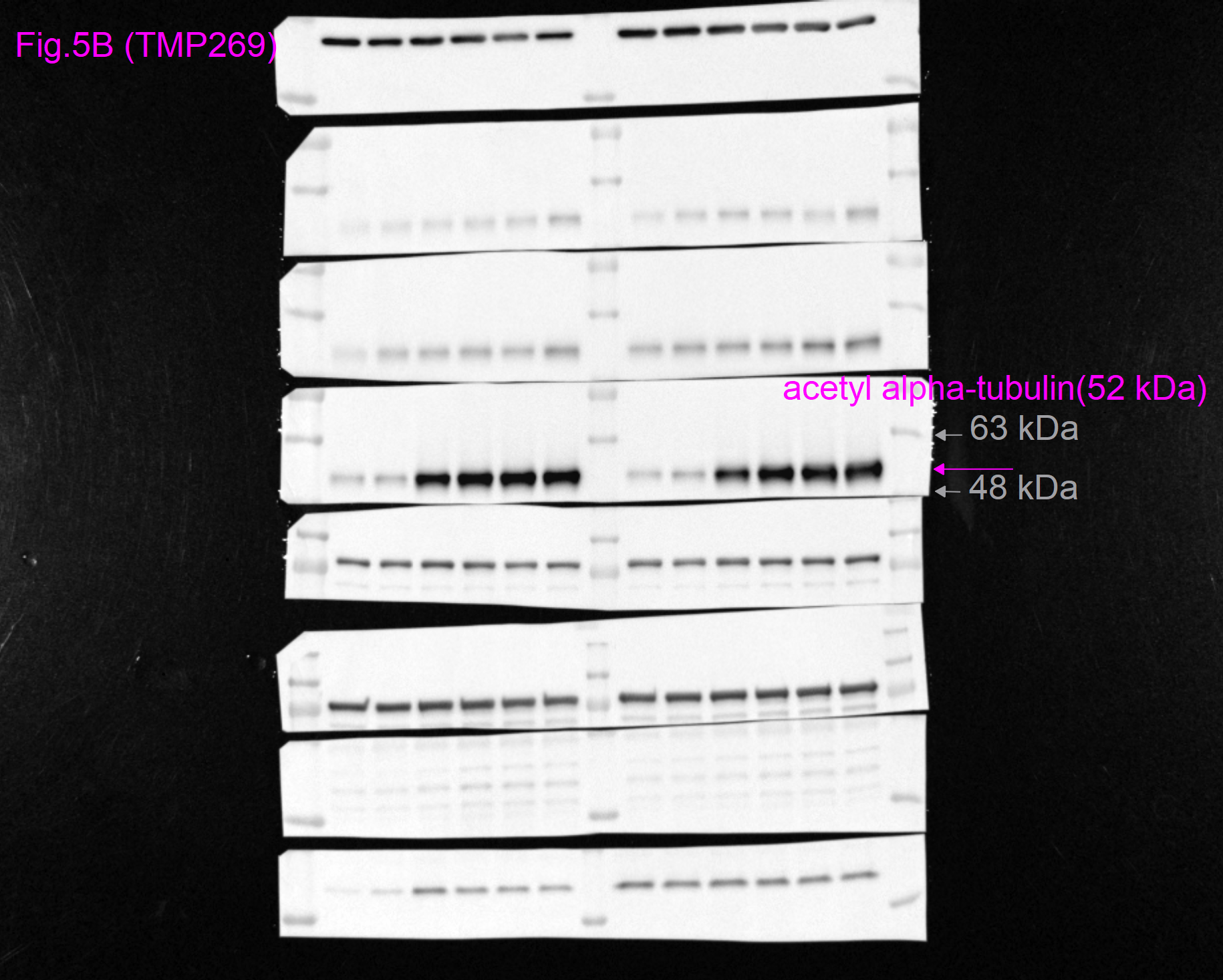

Supplement: Original Image for Fig 5B acetylalphatubulin TMP269.tif [file IENZ_A_2406025_SM9259.tif]

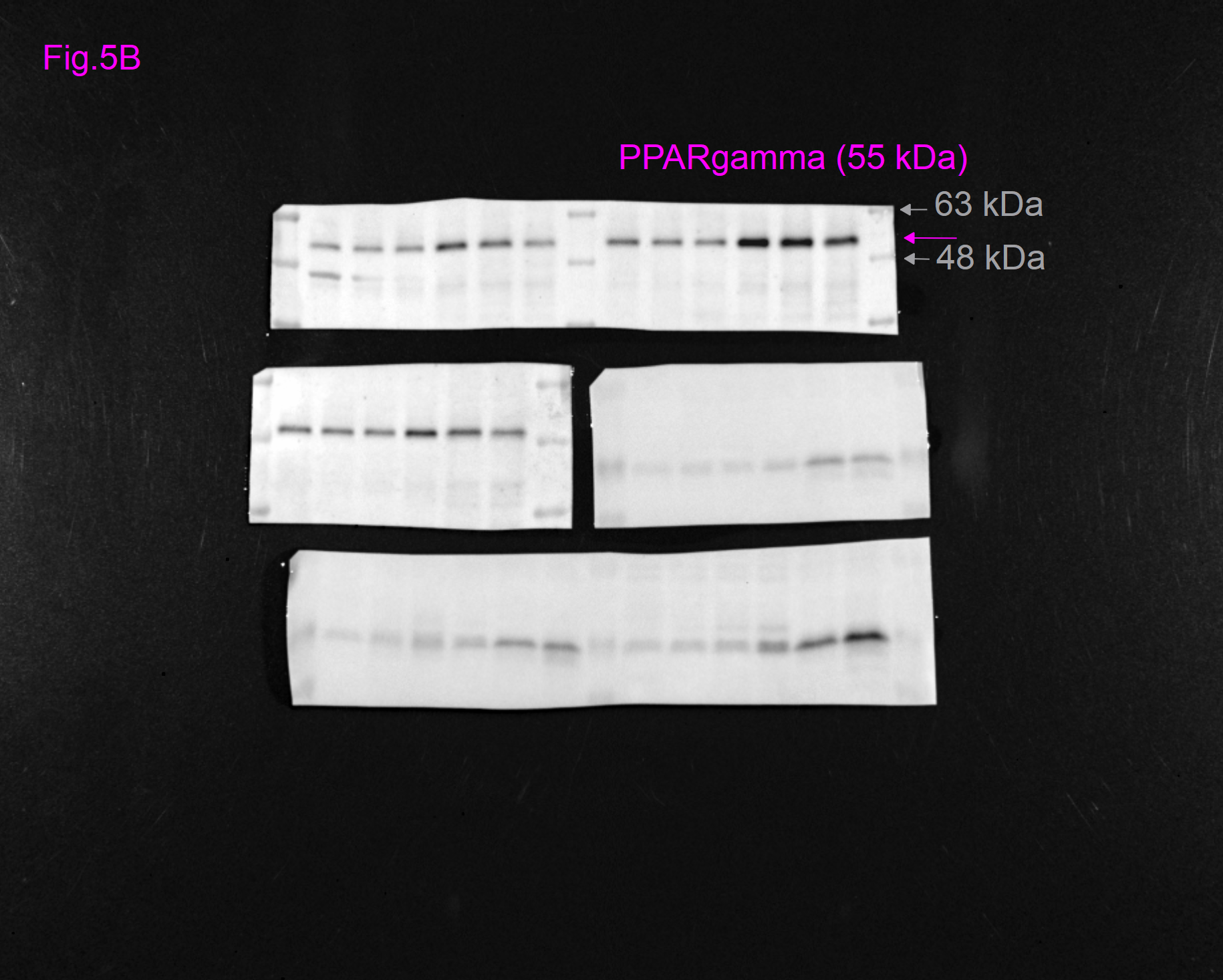

Supplement: Original Image for Fig 5B PPARr 7g.tif [file IENZ_A_2406025_SM9258.tif]

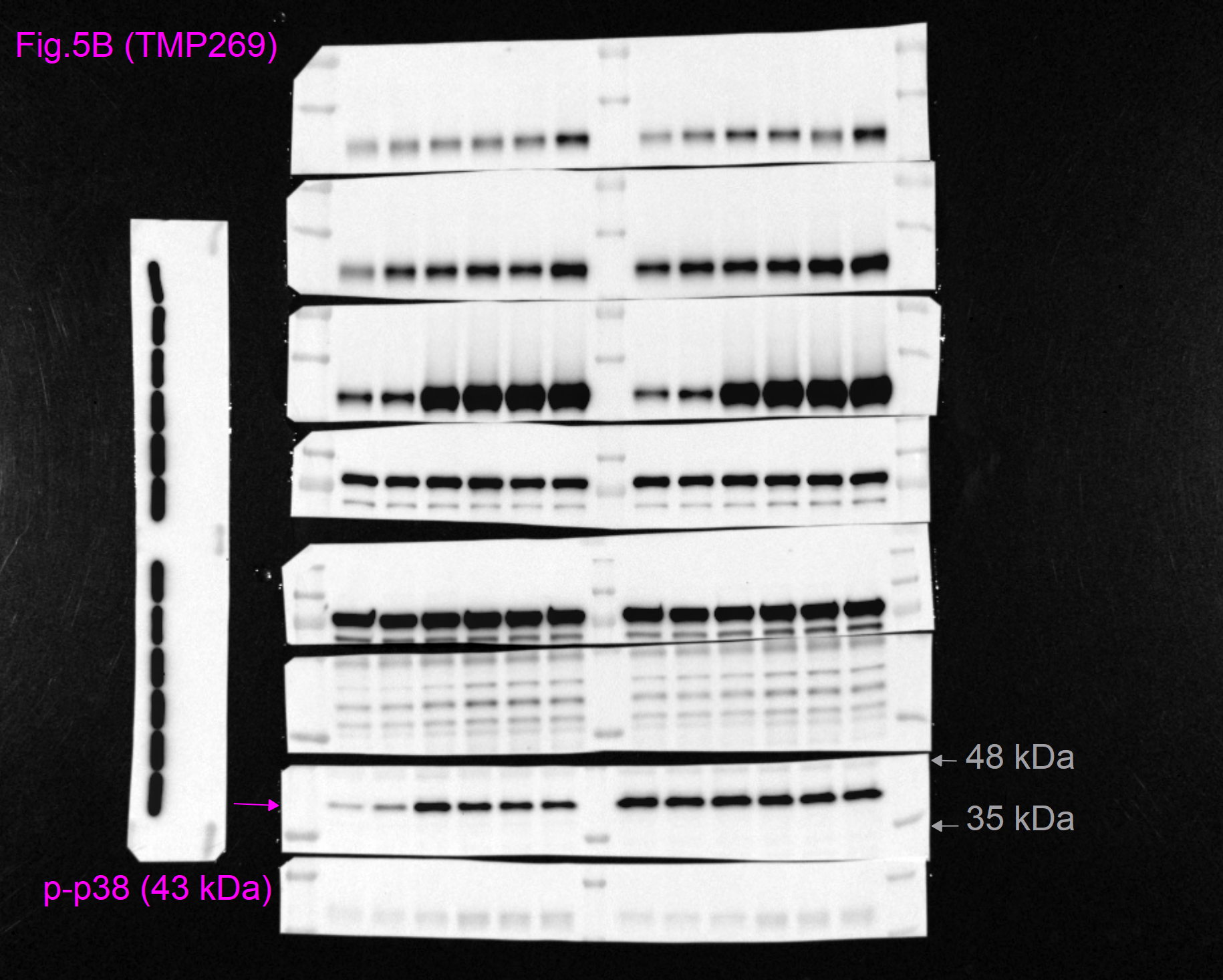

Supplement: Original Image for Fig 5B pp38 TMP269.tif [file IENZ_A_2406025_SM9257.tif]

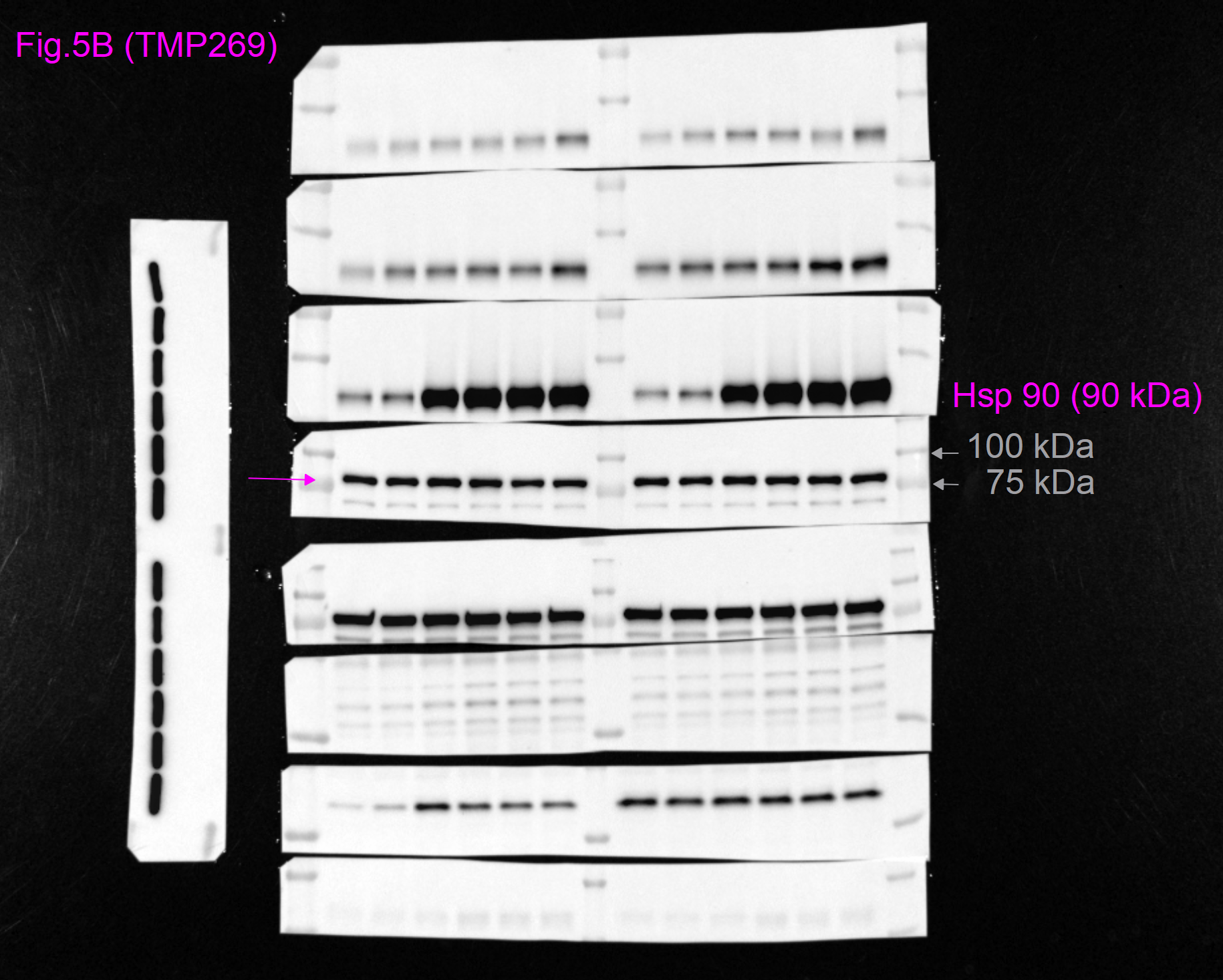

Supplement: Original Image for Fig 5B Hsp90 TMP269.tif [file IENZ_A_2406025_SM9256.tif]

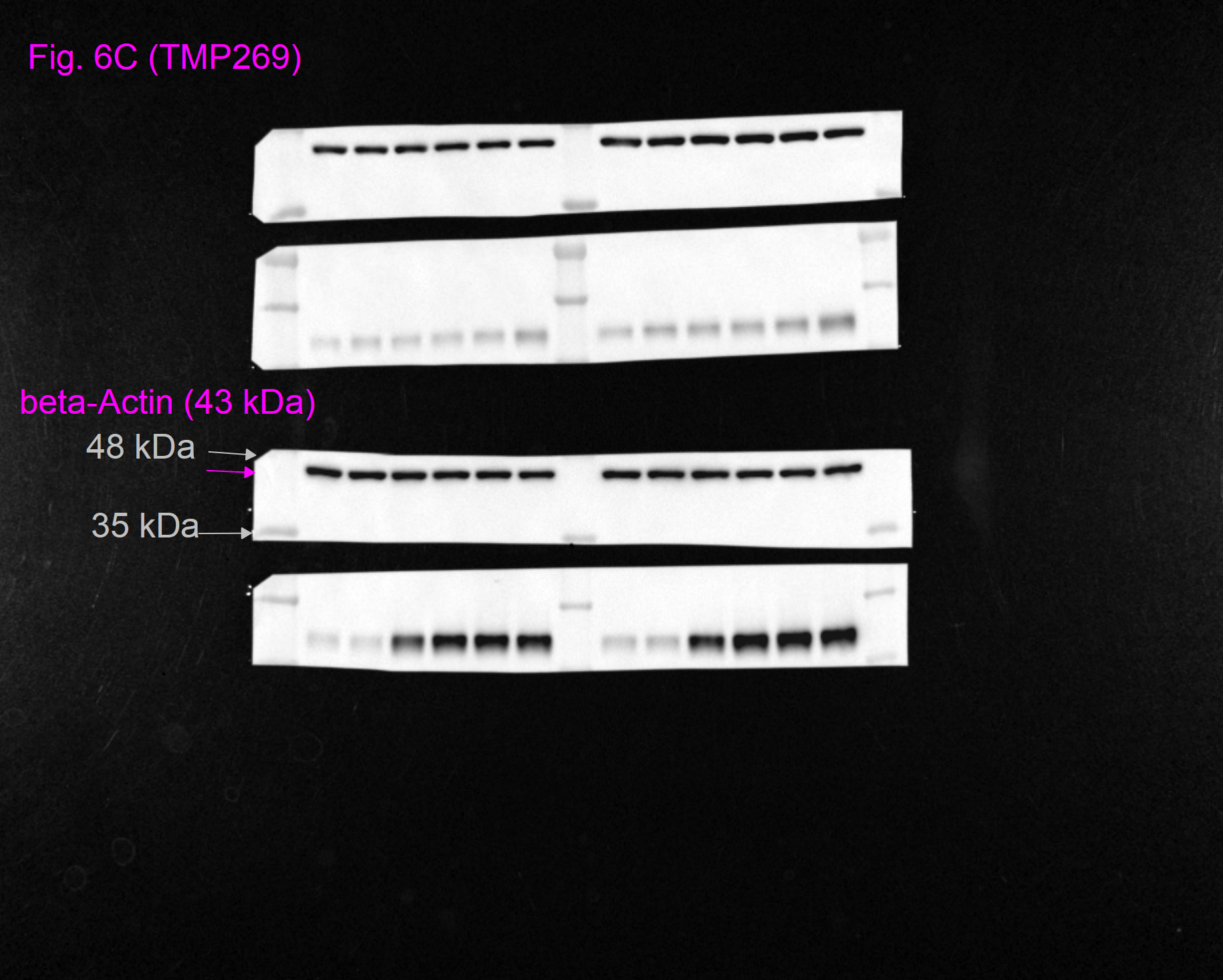

Supplement: Original Image for Fig 6C beta actin TMP269.tif [file IENZ_A_2406025_SM9254.tif]
