# Supplementary material for: Synthesis and biological evaluation of ortho-phenyl phenylhydroxamic acids containing phenothiazine with improved selectivity for class IIa histone deacetylases
Source: J Enzyme Inhib Med Chem. 2024 Sep 24;39(1):2406025. doi: 10.1080/14756366.2024.2406025 (PMC11423540; doi:10.1080/14756366.2024.2406025)
Supplement: supporting information 20240819.docx [file IENZ_A_2406025_SM9255.docx]

**Supplementary Material**

Synthesis and biological evaluation of *ortho*-phenyl phenylhydroxamic acids containing phenothiazine with improved selectivity for class IIa histone deacetylases

Kai-Cheng Hsu^a,b,c,d,1^, Yun-Yi Huang^a,1^, Jung-Chun Chu^a^, Yu-Wen Huang^e^, Jing-Lan Hu^f^, Tony Eight Lin^b,c^, Shih-Chung Yen^g^, Jing-Ru Weng^f*^, Wei-Jan Huang^a,e,h*^

*^a^Ph.D. Program in Drug Discovery and Development Industry, College of Pharmacy, Taipei Medical University, Taipei, Taiwan*

*^b^Graduate Institute of Cancer Biology and Drug Discovery, College of Medical Science and Technology, Taipei Medical University, Taipei, Taiwan*

*^c^Ph.D. Program for Cancer Molecular Biology and Drug Discovery, College of Medical Science and Technology, Taipei Medical University, Taipei, Taiwan*

*^d^Cancer Center, Wan Fang Hospital, Taipei Medical University, Taipei, Taiwan*

*^e^Graduate Institute of Pharmacognosy, College of Pharmacy, Taipei Medical University, Taipei, Taiwan*

*^f^Department of Marine Biotechnology and Resources, National Sun Yat-sen University, Kaohsiung, Taiwan*

*^g^Warshel Institute for Computational Biology, The Chinese University of Hong Kong*

*(Shenzhen), Shenzhen, Guangdong, People’s Republic of China*

*^h^School of Pharmacy, Taipei Medical University, Taipei, Taiwan*

*Corresponding authors

E-mail: wjhuang@tmu.edu.tw (W.J. Huang)

E-mail: jrweng@mail.nsysu.edu.tw (J.R. Weng)

^1^ These authors contributed equally to this work.

**Figure 1S.** Surface plasmon resonance of HDAC4 binding to compound **7g**

**
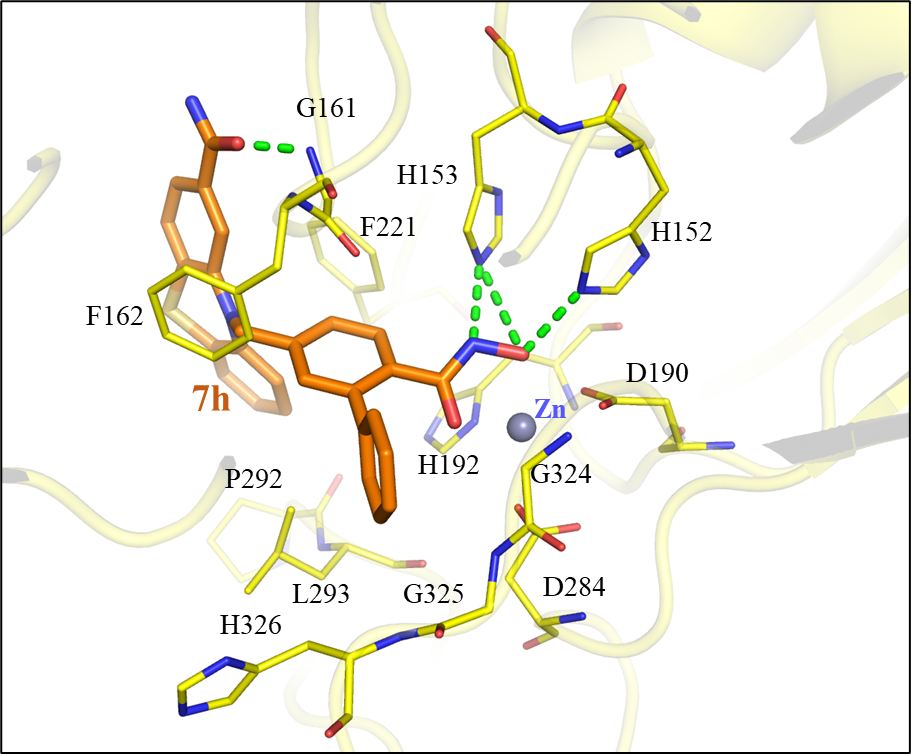
**

**Figure 2S.** Docking pose of compound **7h** in HDAC9 binding site. Compound **7h** (orange) was molecularly docked into the HDAC9 binding site (yellow). Coordination of the zinc ion was observed, and additional interactions with binding site residues suggest favorable occupation of the HDAC9 binding site. Binding site residues are labeled and depicted as lines. Hydrogen bonds are denoted as green dashes.

**Table 1S**. IC_50_ values (μM) of cytotoxicities of compounds **7a-j** and **19a-c** against human oral cancer SCC2095 cells

| Compound | SCC2095 |
| --- | --- |
| **7a** | 3.9+0.9 |
| **7b** | 5.0+0.9 |
| **7c** | 6.0+2.6 |
| **7d** | 3.2+1.7 |
| **7e** | 2.9+0.2 |
| **7f** | 4.6+1.6 |
| **7g** | 3.0+0.3 |
| **7h** | 3.6+0.2 |
| **7i** | >10 |
| **7j** | >10 |
| **19a** | 7.6+3.7 |
| **19b** | 1.8+0.3 |
| **19c** | 2.6+0.4 |
| **Etoposide** | 2.3+0.2 |
| **Vorinostat** | 2.4+0.1 |

**Table 2S.** IC_50_ values^a^ of enzyme-inhibiting activity by compound **7g** against HDAC isoform enzymes.

| **Compound** | HeLa nuclear HDACs (μM) | HDAC8 (μM) | HDAC4 (nM) | HDAC7 (nM) | HDAC9 (nM) | HDAC6 (nM) |
| --- | --- | --- | --- | --- | --- | --- |
| **7g** | >40 | 1.95+0.18 | 180±60 | 190±40 | 40±1 | 970±50 |
| **Vorinostat** | 0.04±0.002 | 1.05+0.06 | 30280±5810 | >40000 | >40000 | 10±1 |

^a^The values were obtained from at least three independent experiments.

**EDC/NHC protein crosslinking**

HDAC4 protein was crosslinked by EDC (1-ethyl-3-(3-dimethylaminopropyl) carbodiimide) and NHS (N-hydroxysuccinimide). The crosslinking reagent and protein were co- incubated 30 minutes at 25°C.

**Surface plasmon resonance (SPR)**

Binding reactivity to mutant HDAC4 protein was performed by SPR (OpenSPR, Nicoyalife). The recombinant HDAC4 protein was crosslinked by EDC/NHC regent, followed by dilution to a concentration of 50 μg/mL with PBS and immobilized onto a amine sensor chip (Nicoyalife). The analytes were tested at various dilutions in PBST, and the flow rate was maintained at 20 μL/min during analysis. The amine sensor chip was regenerated with 10 mM glycine−HCl, pH2.5. The results were analyzed using TraceDrawer software (Nicoyalife).

**Methyl 4-methyl-2-phenylbenzoate (3)**

To a solution of Methyl 2-bromo-4-methylbenzoate **2** (15.0 g, 65.51 mmol) in Dioxane-H_2_O =3: 2 (150 mL) was added Phenylboronic acid (9.6 g, 78.60 mmol), Pd(OAC)_2_ (441 mg, 1.97 mmol), Ph_3_P (2.0 g, 7.86 mmol) and K_2_CO_3_ (27.1 g, 196.50 mmol). The resultant solution was heated to 110 ℃ under N_2_ for 1 h. The reaction mixture was filtered through a celite pad. The filtrate was extracted with H_2_O (50 mL) and EtOAC (3 × 50 mL). The combined organic layer was dried over Na_2_SO_4,_ filtered and the solvent was removed in vacuo. The residue was purified using silica gel chromatography (*n*-hexane: CH_2_Cl_2_ = 4: 1) to give compound **3** (13.8 g, 93%). ^1^H NMR (Acetone-*d*_6_, 300 MHz) δ 7.69 (d, *J* = 7.8 Hz, 1H), 7.40 (t, *J* = 2.1 Hz, 1H), 7.37 (t, *J* = 1.5 Hz, 1H), 7.35 (d, *J* = 1.5 Hz, 1H), 7.29 (m, 2H), 7.27 (m, 1H), 7.22 (d, *J* = 0.9 Hz, 1H), 3.56 (s, 3H), 2.42 (s, 3H), ESI-MS *m/z*: 277 [M+H]^+^.

**Methyl 4-(bromomethyl)-2-phenylbenzoate (4)**

To a mixture of compound **3** (5.0 g, 22.10 mmol) and NBS (5.1 g, 28.71 mmol) in MeCN (10 mL) was added AIBN (363 mg, 2.21 mmol). The resulting solution was heated to 90℃ under N_2_ for 12 h. The reaction mixture was concentrated in vacuo. The residue was purified using silica gel chromatography (EtOAc: *n*-hexane =1: 99) to give compound **4** (8.7 g, 30%).^1^H NMR (Acetone-*d*_6_, 300 MHz,) δ 7.78 (d, *J* = 7.9 Hz, 1H), 7.58 (dd, *J* = 1.9, 7.9 Hz, 1H), 7.52 (d, *J* = 1.8 Hz, 1H), 7.43 (m, 1H) , 7.40 (m, 2H) , 7.34 (d, *J* = 1.9 Hz, 1H), 7.31 (d, *J* = 1.5 Hz, 1H), δ 4.73 (s, 2H), δ 3.9 (s, 3H). ESI-MS *m/z*: 305 [M+H]^+^.

**Methyl 4-[(10*H*-phenothiazin-10-yl)methyl]-2-phenylbenzoate (6a)**

The solution of phenothiazine **5a** (210 mg, 1.0 mmol) and NaH (48 mg, 2.0 mmol) in DMF (10 mL) was stirred for 30 min. Then compound **3** (500 mg, 0.16 mmol) was added to the mixture at 0 ℃. The resulting solution was warmed to RT and stirred for 1 h under N_2_. The reaction mixture was quenched with distd-H_2_O and neutralized to pH 6-7. The mixture was diluted with H_2_O (50 mL) and extracted with EtOAc (3 × 50 mL). The combined organic layer was dried over Na_2_SO_4,_ filtered and the solvent was concentrated in vacuo. The residue was purified by silica gel chromatography (EtOAc: *n*-hexane= 4: 96) to give compound **6a** (300 mg, 70 %). ^1^H NMR (Acetone-*d*_6_, 300 MHz) δ 7.74 (dd, *J* = 1.2, 7.4 Hz, 1H), 7.47 (m, 2H), 7.36 (m, 3H), 7.25 (m, 2H), 7.13 (dd, *J* = 1.4, 7.5 Hz, 2H), 7.09 (dd, *J* = 1.5, 7.4 Hz, 1H), 7.06 (dd, *J* = 1.5, 6.6 Hz, 1H), 6.91 (td, *J* = 1.1, 7.4 Hz, 2H), 6.87 (dd, *J* = 1.1, 8.1 Hz, 2H), 5.31 (s, 2H), 3.58 (s, 3H); ESI-MS *m/z* :424 [M+H]^+^.

**Methyl 4-[(2-chloro-10*H*-phenothiazin-10-yl)methyl]-2-phenylbenzoate (6b)**

Following the procedure as described for **6a**, reaction of compound **5b** (400 mg, 1.71 mmol), compound **4** (782 mg, 2.57 mmol) and NaH (82 mg, 3.42 mmol) in DMF (15 mL) gave compound **6b** (793 mg, 93 %). ^1^H NMR (Acetone-*d*_6_, 300 MHz) δ 7.75 (d, *J* = 7.9 Hz, 1H), 7.50 (m, 2H), 7.37 (m, 3H), 7.26 (m, 2H), 7.14 (m, 2H), 7.10 (dd, *J* = 1.4, 7.7 Hz, 1H), 6.95 (m, 2H), 6.89 (d, *J* = 2 Hz, 1H), 5.34 (s, 2H), 3.58 (s, 3H); ESI-MS *m/z*: 458 [M+H]^+^.

**Methyl 4-[(2-(trifluoromethyl)-10*H*-phenothiazin-10-yl)methyl]-2-phenylbenzoate (6c)**

Following the procedure as described for **6a**, reaction of compound **5c** (400 mg, 1.5 mmol), compound **4** (687 mg, 2.25 mmol) and NaH (72 mg, 3.00 mmol) in DMF (15 mL) gave compound **6c** (573 mg, 78%). ^1^H NMR (Acetone-*d*_6_, 300 MHz) δ 7.75 (d, *J* = 8.43 Hz, 1H), 7.51 (m, 2H), 7.38 (m, 1H), 7.36 (m, 2H), 7.33 (s, 1H), 7.24 (m, 3H), 7.17 (dd, *J* = 1.3, 7.2 Hz, 1H), 7.13 (m, 2H), 6.97 (m, 1H), 6.94 (m, 1H), 5.40 (s, 2H), 3.57 (s, 3H); ESI-MS *m/z*: 492 [M+H]^+^.

**Methyl 4-[(2-methoxy-10*H*-phenothiazin-10-yl)methyl]-2-phenylbenzoate (6d)**

Following the procedure as described for **6a**, reaction of compound **5d** (400 mg, 1.75 mmol), compound **4** (801 mg, 2.63 mmol), and NaH (84 mg, 3.50 mmol) in DMF (15 mL) gave compound **6d** (635 mg, 80 %). ^1^H NMR (Acetone-*d*_6_, 300 MHz) δ 7.74 (d, *J* = 7.8 Hz, 1H), 7.48 (m, 2H), 7.38 (m, 1H), 7.35 (m, 2H), 7.26 (m, 2H), 7.14 (dd, *J* = 1.7, 7.5 Hz, 1H), 7.06 (dd, *J* = 1.9, 8.0 Hz, 1H), 7.02 (d, *J* = 8.4 Hz, 1H), 6.91 (m, 1H), 6.88 (m, 1H), 6.53 (dd, *J* = 2.3, 8.3 Hz, 1H), 6.46 (d, *J* = 2.4 Hz, 1H), 5.31 (s, 2H), 3.65 (s, 3H), 3.57 (s, 3H); ESI-MS *m/z* :454 [M+H]^+^.

**Methyl 4-{[2-(methylthio)-10*H*-phenothiazin-10-yl]methyl}-2-phenylbenzoate (6e)**

Following the procedure as described for **6a**, reaction of compound **5e** (400 mg, 1.63 mmol), compound **4** (746 mg, 2.445 mmol), and NaH (78 mg, 3.26 mmol) in DMF (15 mL) gave compound **6e** (510 mg, 66 %). ^1^H NMR (Acetone-*d*_6_, 300 MHz) δ 7.75 (d, *J* = 7.4 Hz, 1H), 7.50 (m,2H), 7.39 (m, 1H), 7.35 (m, 2H), 7.27 (m, 1H), 7.25 (m, 1H), 7.13 (m, 1H), 7.09 (m, 1H), 7.06 (d, *J* = 8.0 Hz, 1H), 6.92 (d, *J* = 7.5 Hz, 2H), 6.83 (dd, *J* = 1.8, 7.8 Hz, 1H), 6.76 (d, *J* = 1.8 Hz, 1H), 5.33 (s, 2H), 3.57 (s, 3H), 2.30 (s, 3H); ESI-MS *m/z*: 470 [M+H]^+^.

**Methyl 4-[(2-acetyl-10*H*-phenothiazin-10-yl)methyl]-2-phenylbenzoate (6f)**

Following the procedure described for **6a**, reaction of compound **5f** (400 mg, 1.66 mmol), compound **4** (760 mg, 2.49 mmol) and NaH (80 mg, 3.32 mmol) in DMF (20 mL) gave compound **6f** (400 mg, 52 %). ^1^H NMR (Acetone-*d*_6_, 300 MHz) δ 7.74 (d, *J* = 7.9 Hz, 1H), δ 7.53 (m, 3H), 7.38 (m, 2H), 7.35 (m, 2H), 7.25 (m, 3H), 7.14 (m, 1H), 7.11 (dd, *J* = 1.5, 7. Hz, 1H), 6.95 (m, 2H), 5.36 (s, 2H), 3.57 (s, 3H), 2.42 (s, 1H); ESI-MS *m/z*: 466 [M+H]^+^.

**Methyl 4-[(2-cyano-10H-phenothiazin-10-yl)methyl]-2-phenylbenzoate (6g)**

Following the procedure described for **6a**, reaction of compound **6g** (800 mg, 3.75 mmol), compound **4** (1.6 g, 5.36 mmol), and NaH (171 mg, 7.14 mmol) in DMF (30 mL) gave compound **6g** (1.0 g, 62 %). ^1^H NMR (Acetone-*d*_6_, 300 MHz) δ 7.75 (d, *J* = 8.0 Hz, 1H), 7.50 (m, 2H), 7.39 (m, 1H), 7.37 (m, 1H), 7.34 (d, *J* = 1.8, 6.3 Hz, 1H), 7.30 (s, 1H), 7.29 (d, *J* = 1.4 Hz, 1H), 7.27 (d, *J* = 1.8 Hz, 1H), 7.25 (m, 1H), 7.14 (m, 3H), 6.99 (dd, *J* = 1.1, 7.4 Hz, 1H), 6.94 (d, *J* = 8.7 Hz, 1H), 5.38 (s, 2H), 3.58 (s, 3H); ESI-MS *m/z*: 449 [M+H]^+^.

**Methyl 4-[(adamantan-1-ylamino)methyl]-2-phenylbenzoate (9)**

The solution of compound **8** (700 mg, 3.73 mmol) and K_2_CO_3_ (567 mg, 4.10 mmol) in DMF (30 mL) was stirred at RT for 30 min. Then compound **4** (1.1 g, 3.73 mmol) was added to the resulting solution and the reaction mixture was warmed to RT under N_2_ for 24 h. The reaction mixture was diluted with distd-H_2_O, neutralized to pH 6-7, and extracted with EtOAc (3 × 50 mL). The combined organic layer was dried over Na_2_SO_4,_ filtered and the solvent was removed in vacuo. The residue was purified by silica gel chromatography (*n*-hexane: EtOAc= 6: 1) to give compound **9** (400 mg, 30 %). ^1^H NMR (Acetone-*d*_6_, 300 MHz) δ 7.72 (d, *J* = 7.9 Hz, 1H), 7.47 (d, *J* = 8.0 Hz, 1H), 7.43 (m, 1H), 7.40 (dd, *J* = 1.7, 7.2 Hz, 1H), 7.34 (d, *J* = 5.2 Hz, 1H), 7.29 (dd, *J* = 1.8, 8.1 Hz, 1H), 3.87 (s, 2H), 3.57 (s, 3H), 1.72 (d, *J* = 2.8 Hz, 7H), 1.65 (t, *J* = 2.6 Hz, 6H); ESI-MS *m/z*: 376 [M+H]^+^.

**Methyl 4-[(1,3,4,5-tetrahydro-*2H*-benzo[c]azepin-2-yl)methyl]-2-phenylbenzoate (11)**

According to the same procedure described for **6a**, reaction of compound **10** (500 mg, 2.72 mmol), compound **4** (1.25 g, 4.08 mmol), and NaH (130 mg, 5.44 mmol) in DMF (30 mL) gave compound **11** (911 mg, 90 %). ^1^H NMR (Acetone-*d*_6_, 300 MHz) δ 7.72 (d, *J* = 7.9 Hz, 1H), 7.47 (d, *J* = 3 Hz, 1H), 7.37 (m, 1H), 7.34 (d, *J* = 3 Hz, 1H), 7.29 (t, *J* = 2.5 Hz, 1H), 7.13 (m, 2H), 7.07 (m, 1H), 6.94 (d, *J* = 11.4 Hz, 1H), 3.87 (s, 2H), 3.63 (s, 3H), 3.59 (s, 3H), 3.12 (t, *J* = 8.8 Hz, 2H), 1.74 (m, 2H); ESI-MS *m/z*: 371 [M+H]^+^.

**Methyl 4-methyl-2-(3,4,5-trimethoxyphenyl)benzoate (12)**

Following the procedure as described for **3**, reaction of compound **2** (100 mg, 0.43 mmol), compound **16a** (110 mg, 0.52 mmol), Pd(OAc)_2_ (3 mg, 0.01 mmol), Ph_3_P (13 mg, 0.05 mmol), and K_2_CO_3_ (180 mg, 1.30 mmol) in Dioxane-H_2_O = 3: 2 (10 mL) gave compound **12** (133 mg, 98 %). ^1^H NMR (Acetone-*d*_6_, 300 MHz) δ 7.63 (d, *J* = 7.7 Hz, 1H), 7.27 (m, 2H), 6.58 (s, 2H), 3.84 (s, 6H), 3.76 (s, 3H), 3.63 (s, 3H), 2.41 (s, 3H).

**Methyl 4-methyl-2-(2-bromo-3,4,5-trimethoxyphenyl)benzoate (13)**

Following the procedure as described for compound **4**, reaction of compound **12** (100 mg, 0.32 mmol), NBS (73.4 mg, 0.41 mmol), and AIBN (5 mg, 0.03 mmol) in ACN (10 mL) gave compound **13** (98 mg, 78 %). ^1^H NMR (Acetone-*d*_6_, 300 MHz) δ 7.88 (d, *J* = 7.9 Hz, 1H), 7.33 (m, 1H), 7.09 (m, 1H), 6.72 (s, 1H), 3.88 (s, 3H), 3.86 (s, 3H), 3.85 (s, 3H), 3.60 (s, 3H), 2.42 (s, 3H).

**Methyl 2-bromo-4-((2-cyano-10H-phenothiazin-10-yl)methyl)benzoate (15)**

Following the procedure described for **5a**, reaction of compound **14** (3.0 g, 9.74 mmol), compound **5g** (1.4 g, 6.09 mmol), and NaH (292 mg, 12.18 mmol) in DMF (50 mL) gave compound **15** (2.4 g, 88 %). ^1^H NMR (CDCl_3_, 300 MHz) δ 7.78 (d, *J* = 8.0 Hz, 1H), 7.64 (d, *J* = 1.4 Hz, 1H), 7.27 (m, 1H), 7.15 (m, 2H), 7.09 (dd, *J* = 7.5 Hz, 1H), 7.04 (m, 1H), 6.93 (td, *J* =1.2, 7.5 Hz, 1H), 6.71 (m, 1H), 6.58 (dd, *J* = 8.0, 1.1 Hz, 1H), 5.04 (s, 2H), 3.93 (s, 3H); ESI-MS *m/z*: 451 [M+H]^+^.

***Methyl 4-[(2-cyano-10H-phenothiazin-10-yl)methyl]-2-(3,4,5-trimethoxyphenyl)benzoate (17a)***

Following the procedure as described for **3**, reaction of compound **15** (600 mg, 1.33 mmol), compound **16a** (337 mg, 1.59 mmol), Pd(OAc)_2_ (9 mg, 0.04 mmol), Ph_3_P (42 mg, 0.16 mmol), and K_2_CO_3_ (551 mg, 3.99 mmol) in Dioxane-H_2_O (30 mL-20 mL) gave compound **17a** (480 mg, 67 %). ^1^H NMR (CDCl_3_, 300 MHz) δ 7.74 (d, *J* = 7.9 Hz, 1H), 7.31 (m, 2H), 7.13 (s, 2H), 7.04 (m, 2H), 6.93 (td, *J* = 1.1, 7.4 Hz, 1H), 6.78 (m, 1H), 6.65 (dd, *J* = 1.1, 8.1 Hz), 6.49 (s, 2H), 5.11 (s, 2H), 3.88 (s, 3H), 3.84 (s, 6H), 3.68 (s, 3H) ; ESI-MS *m/z*: 539 [M+H]^+^.

***Methyl 4-[(2-cyano-10H-phenothiazin-10-yl)methyl]-2-(4-methoxyphenyl)benzoate (17b)***

Following the procedure as described for **3**, reaction of compound **15** (500 mg, 1.11 mmol), compound **16b** (202 mg, 1.33 mmol), Pd(OAc)_2_ (7 mg, 0.03 mmol), Ph_3_P (35 mg, 0.13 mmol), and K_2_CO_3_ (459 mg, 3.32 mmol) in Dioxane-H_2_O (30 mL-20 mL) gave compound **17b** (496 mg, 93%). ^1^H NMR (CDCl_3_, 300 MHz) δ 7.77 (d, *J* = 7.9 Hz, 1H), 7.28 (m, 2H), 7.21 (d, *J* = 8.8 Hz, 2H), 7.13(m, 2H ), 7.04 (m, 2H), 6.92 (m, 3H), 6.77 (s, 1H), 6.64 (dd, *J* = 1.1, 8.2 Hz, 1H), 5.09 (m, 2H), 3.84 (s, 3H), 3.67 (s, 3H); ESI-MS *m/z*: 479 [M+H]^+^.

**Methyl 4-[(2-cyano-10H-phenothiazin-10-yl)methyl]-2-(4-fluorophenyl)benzoate (17c)**

Following the procedure as described for **3**, reaction of compound **15** (500 mg, 1.11 mmol), compound **16c** (186 mg, 1.33 mmol), Pd(OAc)_2_ (7 mg, 0.03 mmol), Ph_3_P (35 mg, 0.13 mmol), and K_2_CO_3_ (459 mg, 3.32 mmol) in Dioxane-H_2_O (30 mL-20 mL) gave compound **17c** (482 mg, 93%). ^1^H NMR (CDCl_3_, 300 MHz) δ 7.82 (d, *J* = 7.9 Hz, 1H), 7.29 (m, 3H), 7.22 (m, 1H), 7.12 (m, 3H), 7.04 (m, 3H), 6.93 (td, *J* = 1.1, 7.4 Hz, 1H), 6.76 (d, *J* = 0.6 Hz, 1H), 6.63 (d, *J* = 8.1 Hz, 1H), 5.10 (s, 2H), 3.66 (s, 3H); ESI-MS *m/z*: 467 [M+H]^+^.

**4-[(2-Cyano-10H-phenothiazin-10-yl)methyl]-2-(3,4,5-trimethoxyphenyl)benzoic acid (18a)**

The reaction solution of compound **17a** (300 mg, 0.56 mmol), and LiOH (70 mg, 1.67 mmol) in THF-MeOH-H_2_O (6 mL-2 mL-2 mL) was heated to 45 ^o^C under N_2_ for 18 h. The reaction mixture was diluted with distd-H_2_O (50 mL), acidified with 1N HCl to pH = 2, and extracted with EtOAc (3 × 50 mL). The combined organic layer was dried over Na_2_SO_4,_ filtered and the solvent was concentrated in vacuo. The residue was purified by silica gel chromatography (MeOH: CH_2_Cl_2_= 3: 97) to give compound **18a** (185 mg, 63%). ^1^H NMR (CDCl_3_, 300 MHz) δ 7.89 (d, *J* = 8.4 Hz, 1H), 7.31 (m, 2H), 7.14 (s, 2H), 7.05 (m, 2H), 6.93 (td, *J* = 1.1, 3.7 Hz, 1H), 6.77 (s, 1H), 6.64 (dd, *J* = 1.1, 8.1 Hz, 1H), 6.52 (s, 2H), 5.11 (s, 2H), 3.88 (s, 3H), 3.83 (s, 6H); ESI-MS *m/z*: 525 [M+H]^+^.

***4-[(2-Cyano-10H-phenothiazin-10-yl)methyl]-2-(4-methoxyphenyl)benzoic acid (18b)***

Following the procedure as described for **18a**, reaction of compound **17b** (350 mg, 0.73 mmol), and LiOH (92 mg, 2.19 mmol) in THF-MeOH-H_2_O (7.5 mL-2.5 mL-2.5 mL) gave compound **18b** (270 mg, 80%). ^1^H NMR (Acetone-*d*_6_, 300 MHz) δ 7.79 (dd, *J* = 1.5, 7.1 Hz, 1H), 7.44 (m, 2H), 7.29 (m, 2H), 7.24 (d, *J* = 8.8 Hz, 2H), 7.14 (m, 3H), 6.98 (m, 1H), 6.93 (d, *J* = 8.8 Hz, 2H), 6.92 (m, 1H), 5.36 (s, 2H), 3.81 (s, 3H) ; ESI-MS *m/z*: 465 [M+H]^+^.

***4-[(2-Cyano-10H-phenothiazin-10-yl)methyl]-2-(4-fluorophenyl)benzoic acid (18c)***

Following the procedure as described for **18a**, reaction of compound **17c** (400 mg, 0.86 mmol), and LiOH (108 mg, 2.57 mmol) in THF-MeOH-H_2_O (12 mL-4 mL-4 mL) gave compound **18c** (307 mg, 79%). ^1^H NMR (Acetone-*d*_6_, 300 MHz) δ 7.86 (dd, *J* = 0.8, 7.5 Hz, 1H), 7.50 (m, 2H), 7.33 (m, 2H), 7.30 (m, 2H), 7.14 (m, 6H), 6.98 (td, *J* = 1.2, 7.5 Hz, 1H), 6.94 (dd, *J* = 0.9, 8.2 Hz, 1H), 5.38 (s, 2H); ESI-MS *m/z*: 453 [M+H]^+^.


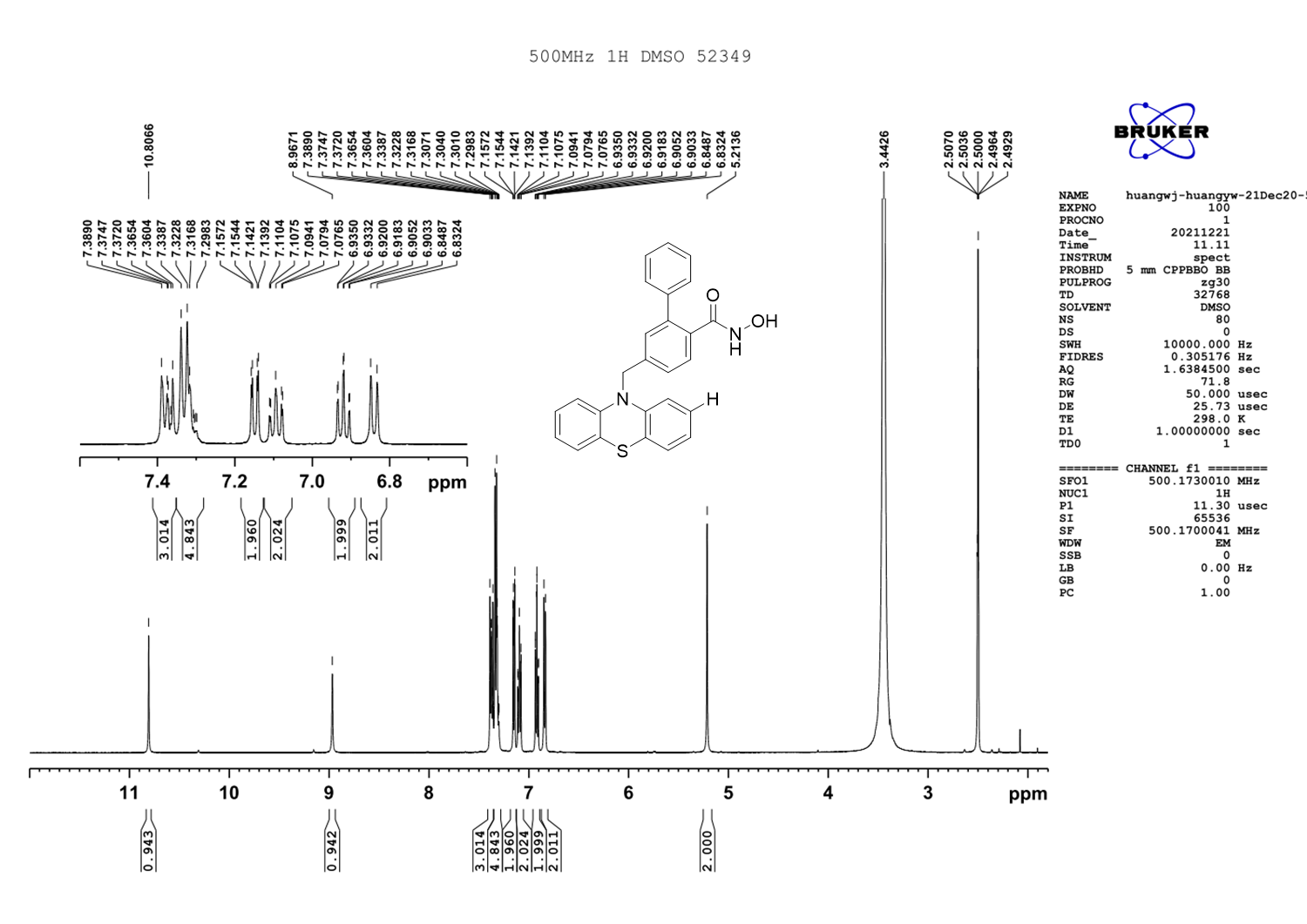


**Figure 3S.** ^1^H-NMR spectrum of compound **7a** (DMSO-*d_6_*, 500 MHz)


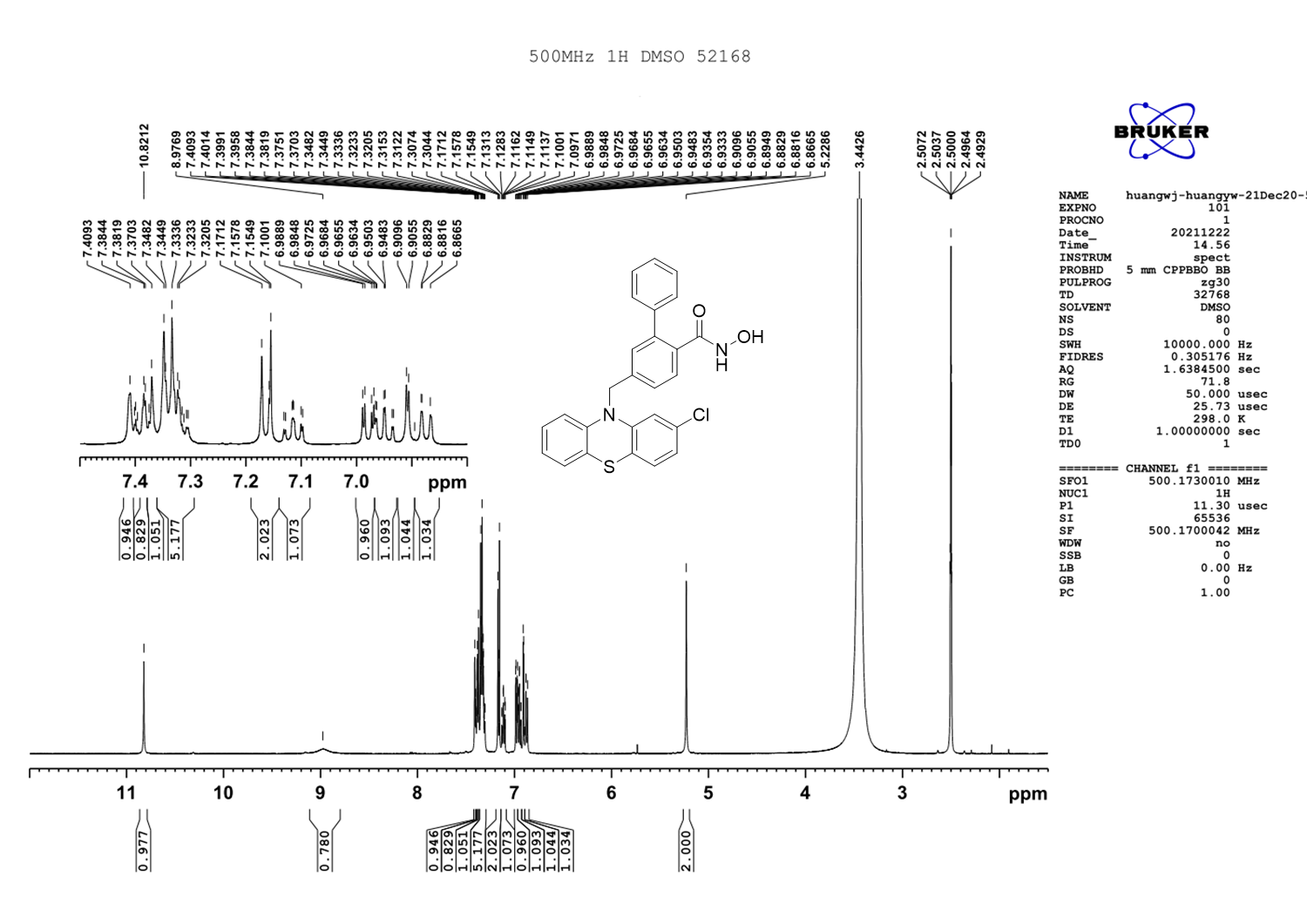


**Figure 4S.** ^1^H-NMR spectrum of compound **7b** (DMSO-*d_6_*, 500 MHz)


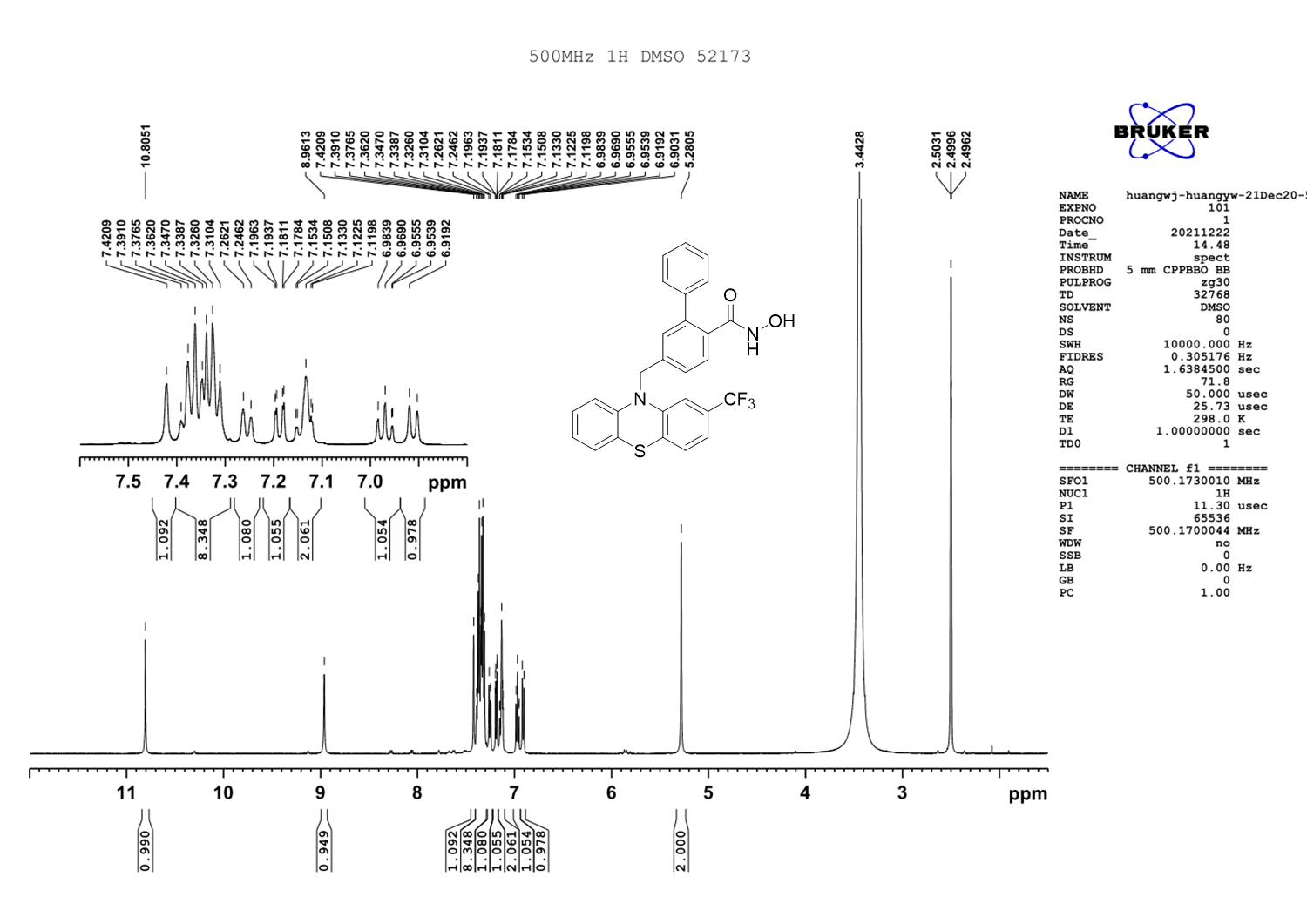


**Figure 5S.** ^1^H-NMR spectrum of compound **7c** (DMSO-*d_6_*, 500 MHz)


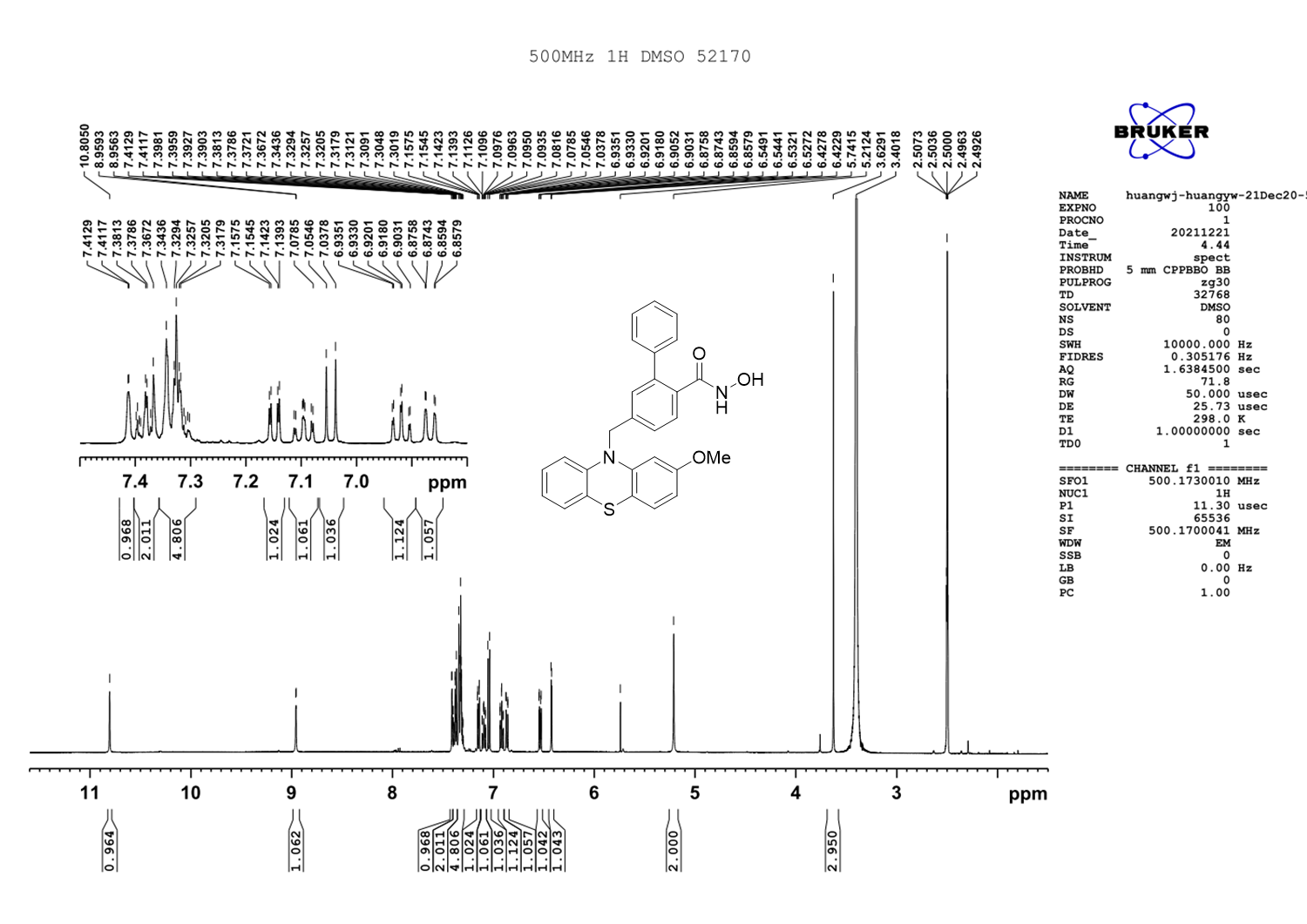


**Figure 6S.** ^1^H-NMR spectrum of compound **7d** (DMSO-*d_6_*, 500 MHz)


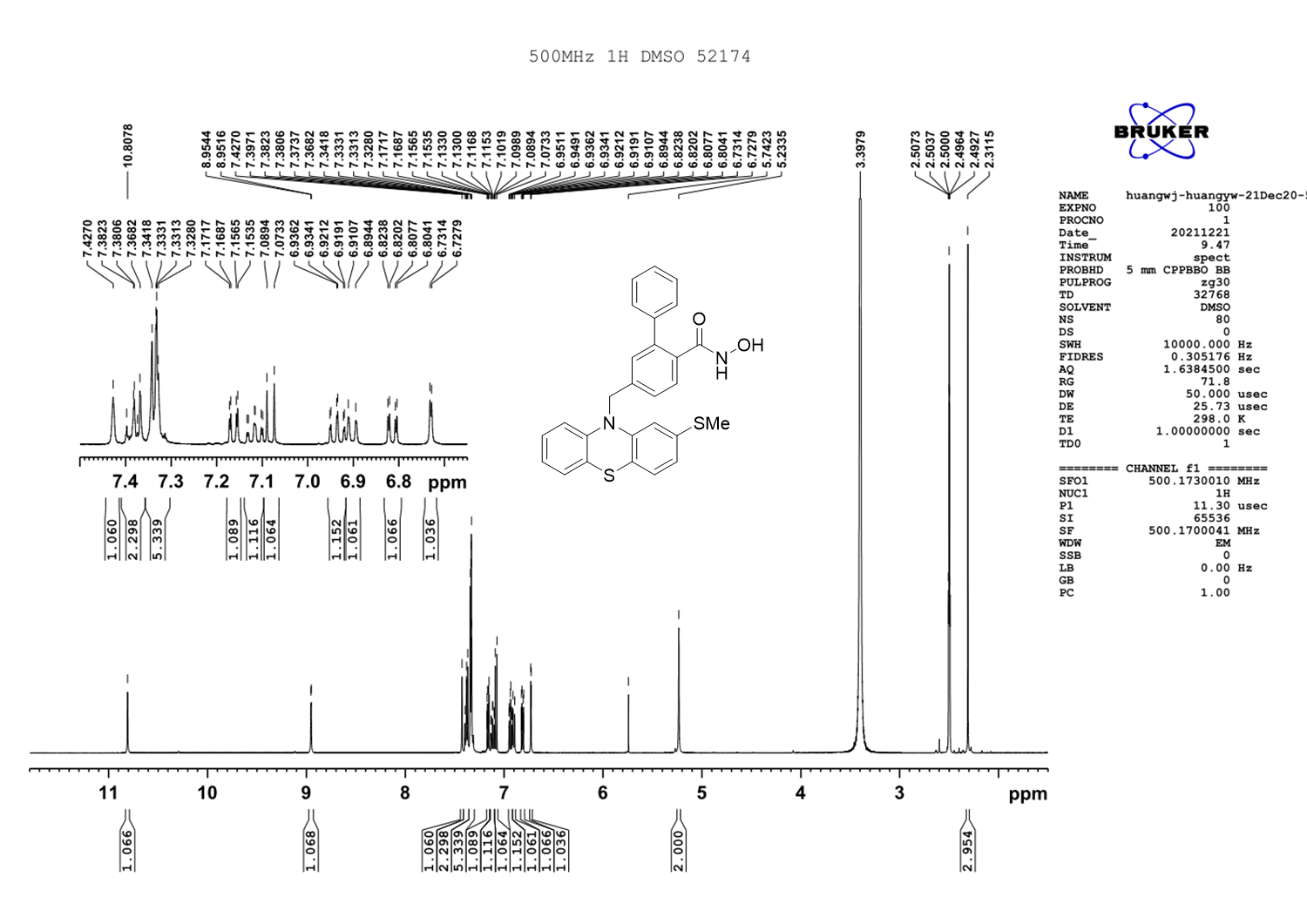


**Figure 7S.** ^1^H-NMR spectrum of compound **7e** (DMSO-*d_6_*, 500 MHz)


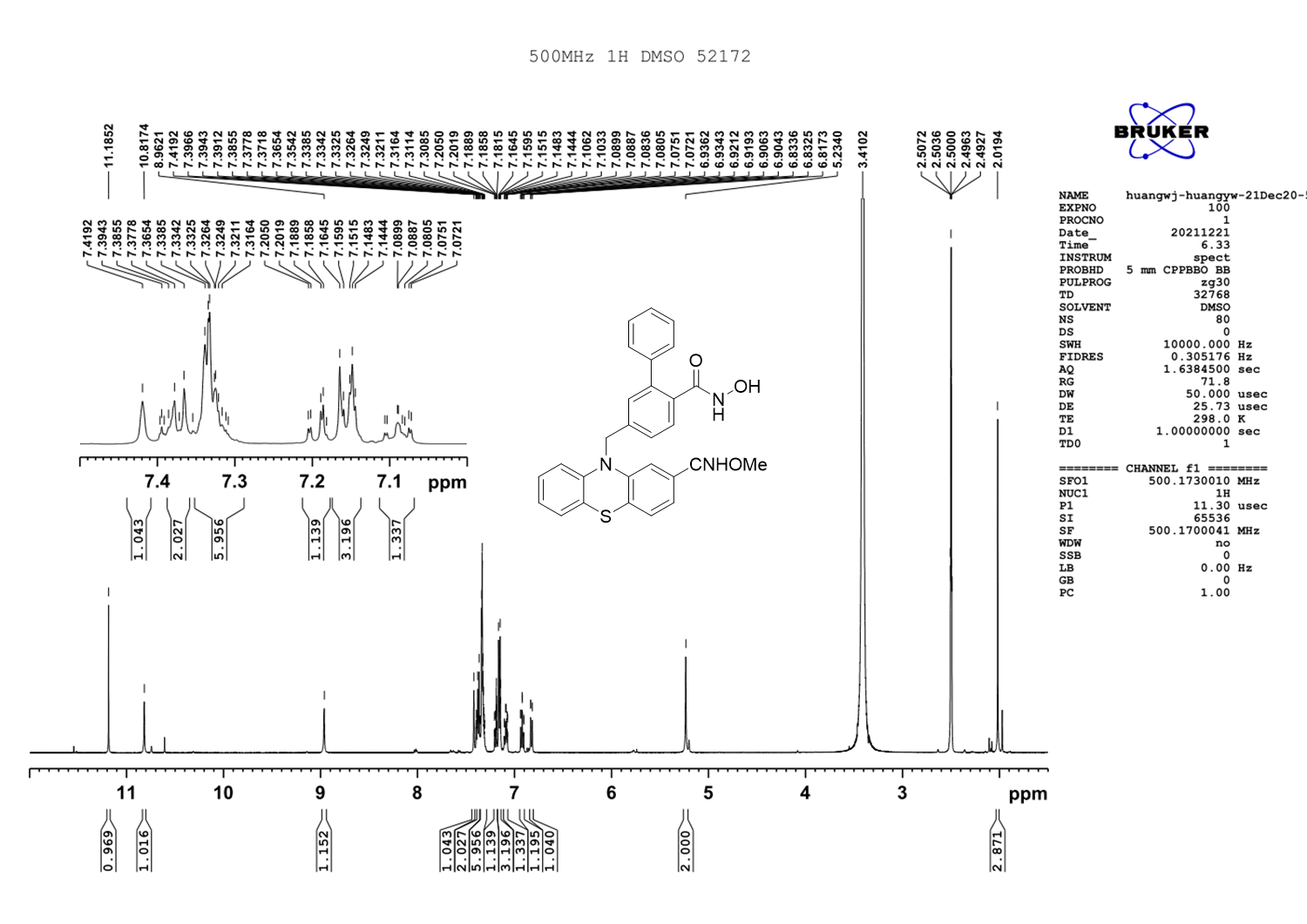


**Figure 8S.** ^1^H-NMR spectrum of compound **7f** (DMSO-*d_6_*, 500 MHz)


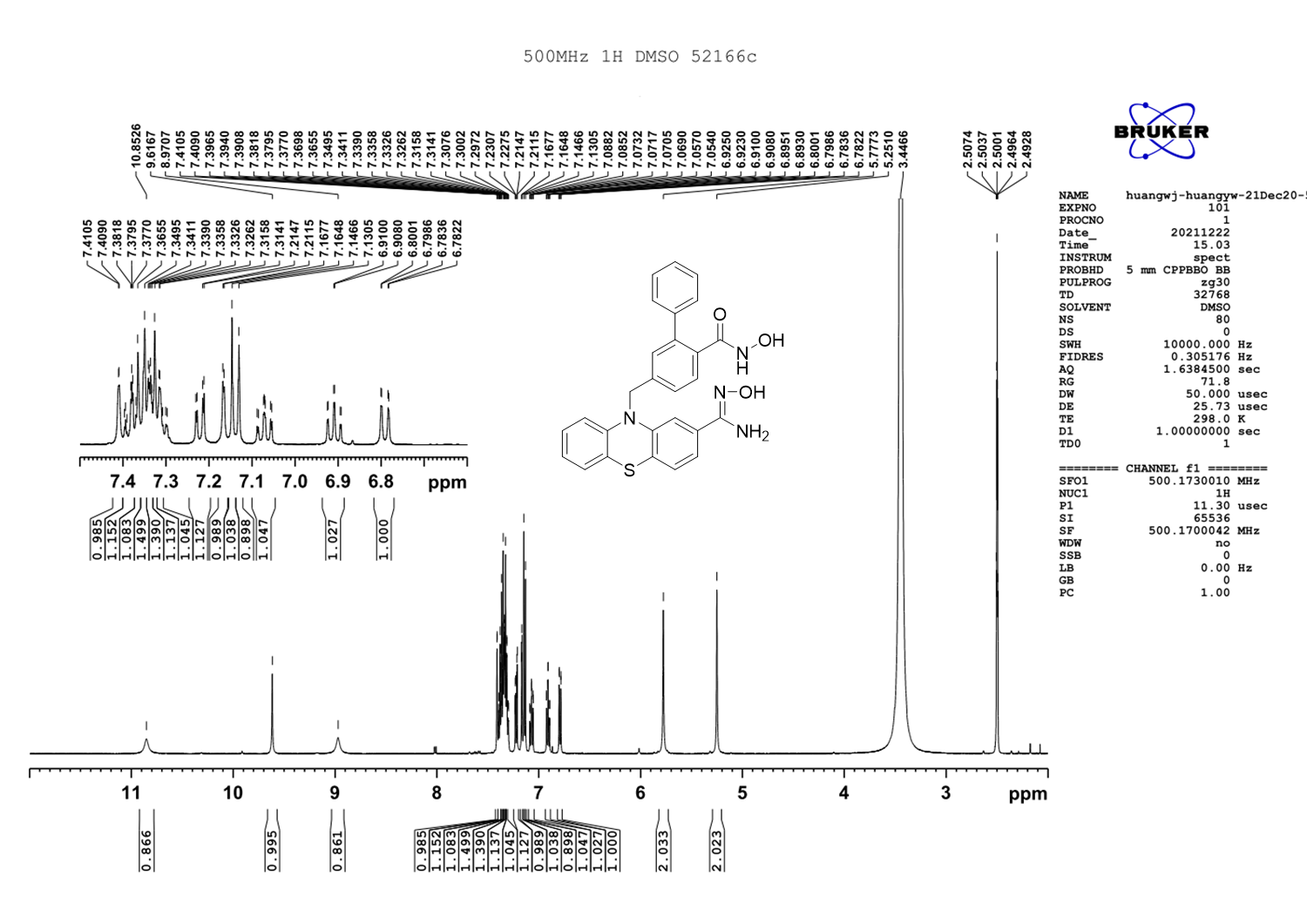


**Figure 9S.** ^1^H-NMR spectrum of compound **7g** (DMSO-*d_6_*, 500 MHz)


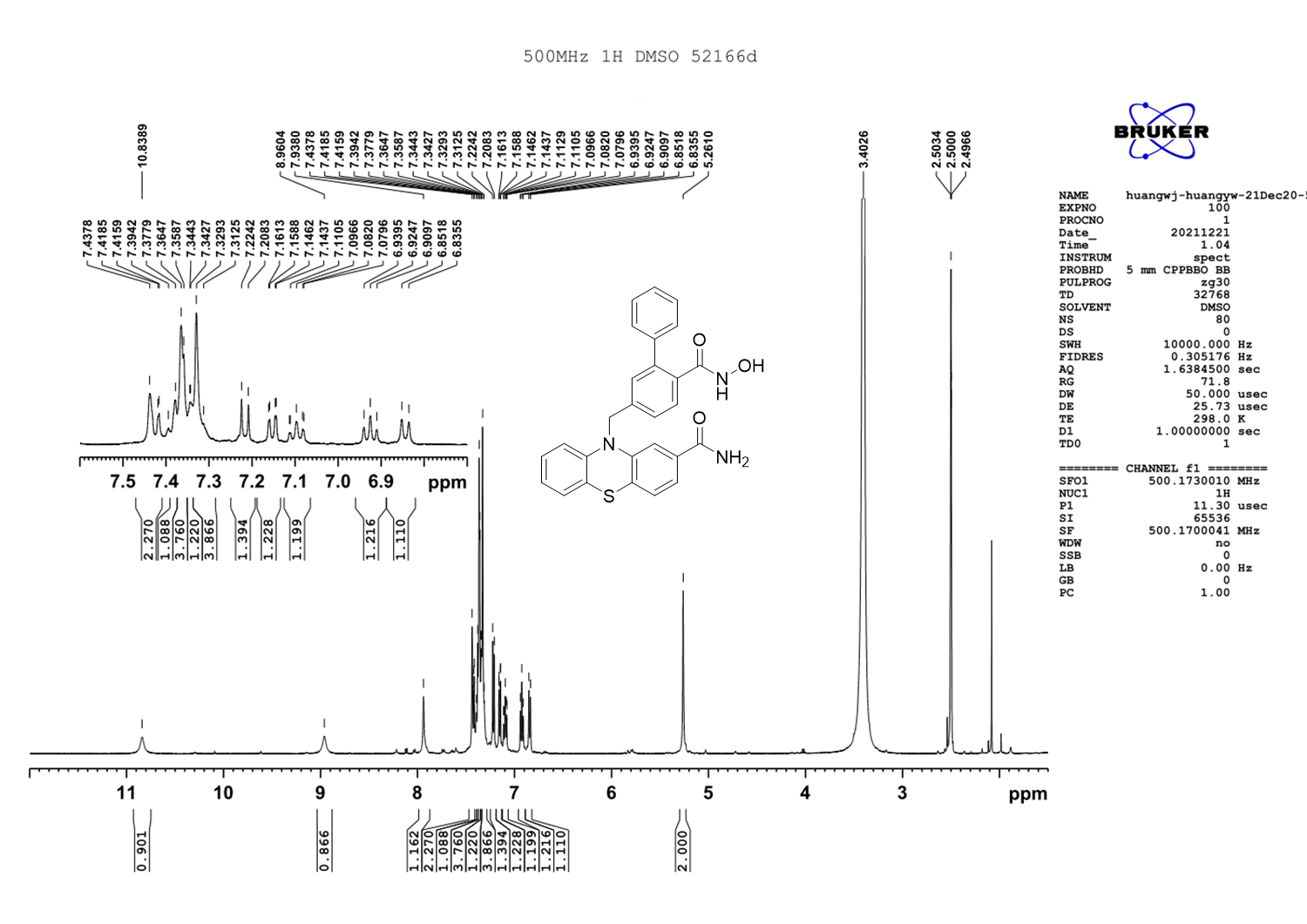


**Figure 10S.** ^1^H-NMR spectrum of compound **7h** (DMSO-*d_6_*, 500 MHz)


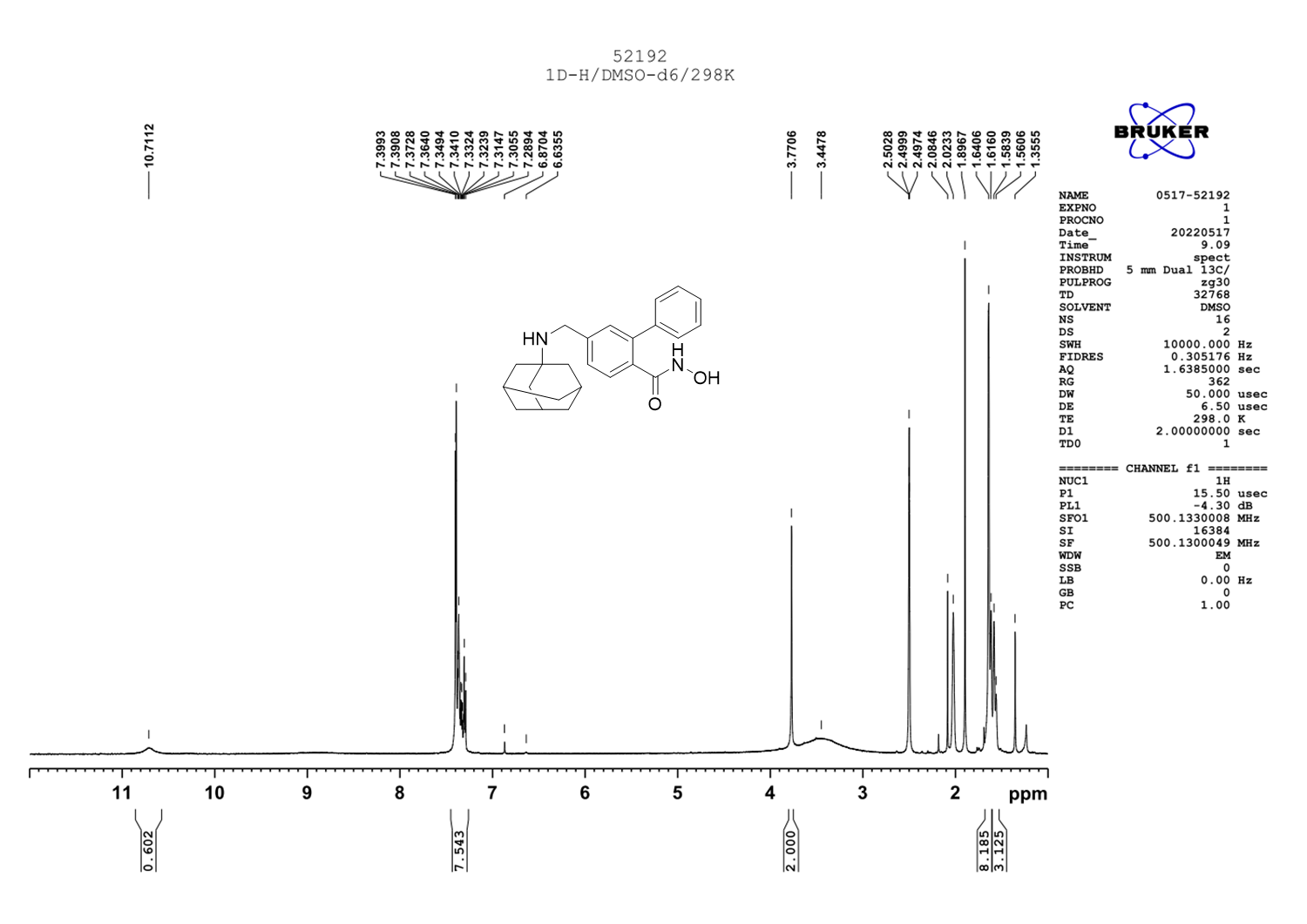


**Figure 11S.** ^1^H-NMR spectrum of compound **7i** (DMSO-*d_6_*, 500 MHz)


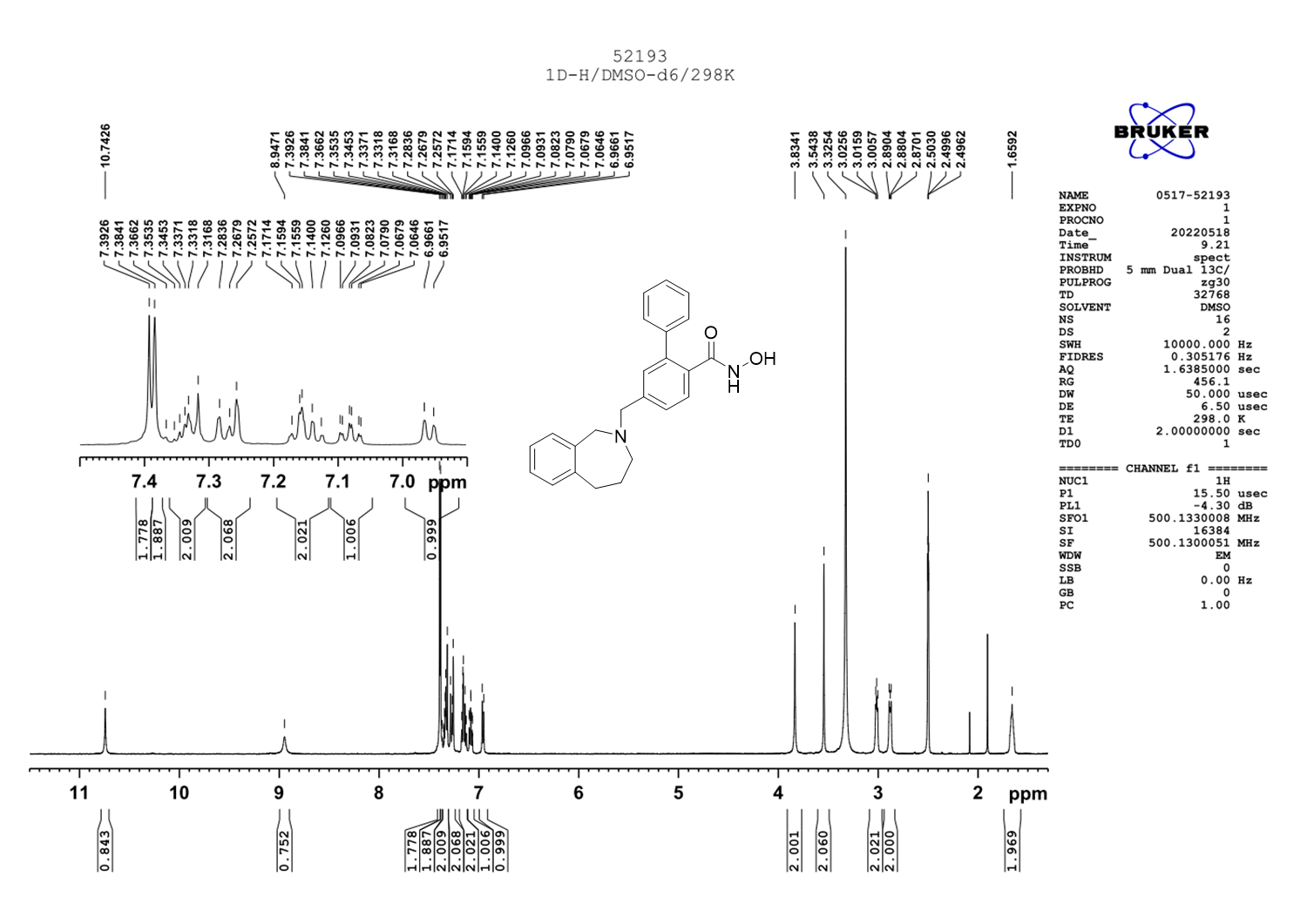


**Figure 12S.** ^1^H-NMR spectrum of compound **7j** (DMSO-*d_6_*, 500 MHz)


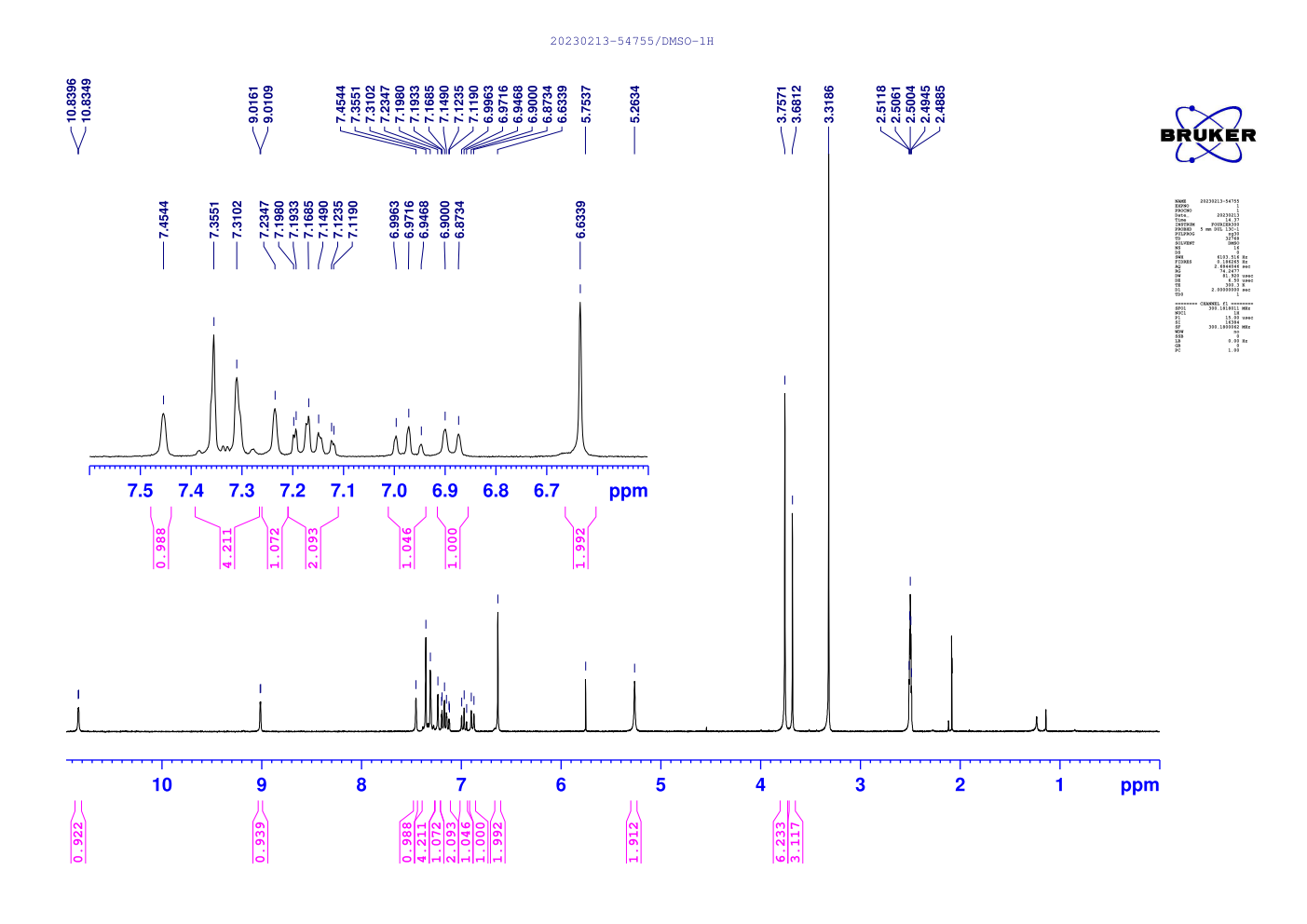


**Figure 13S.** ^1^H-NMR spectrum of compound **19a** (DMSO-*d_6_*, 300 MHz)


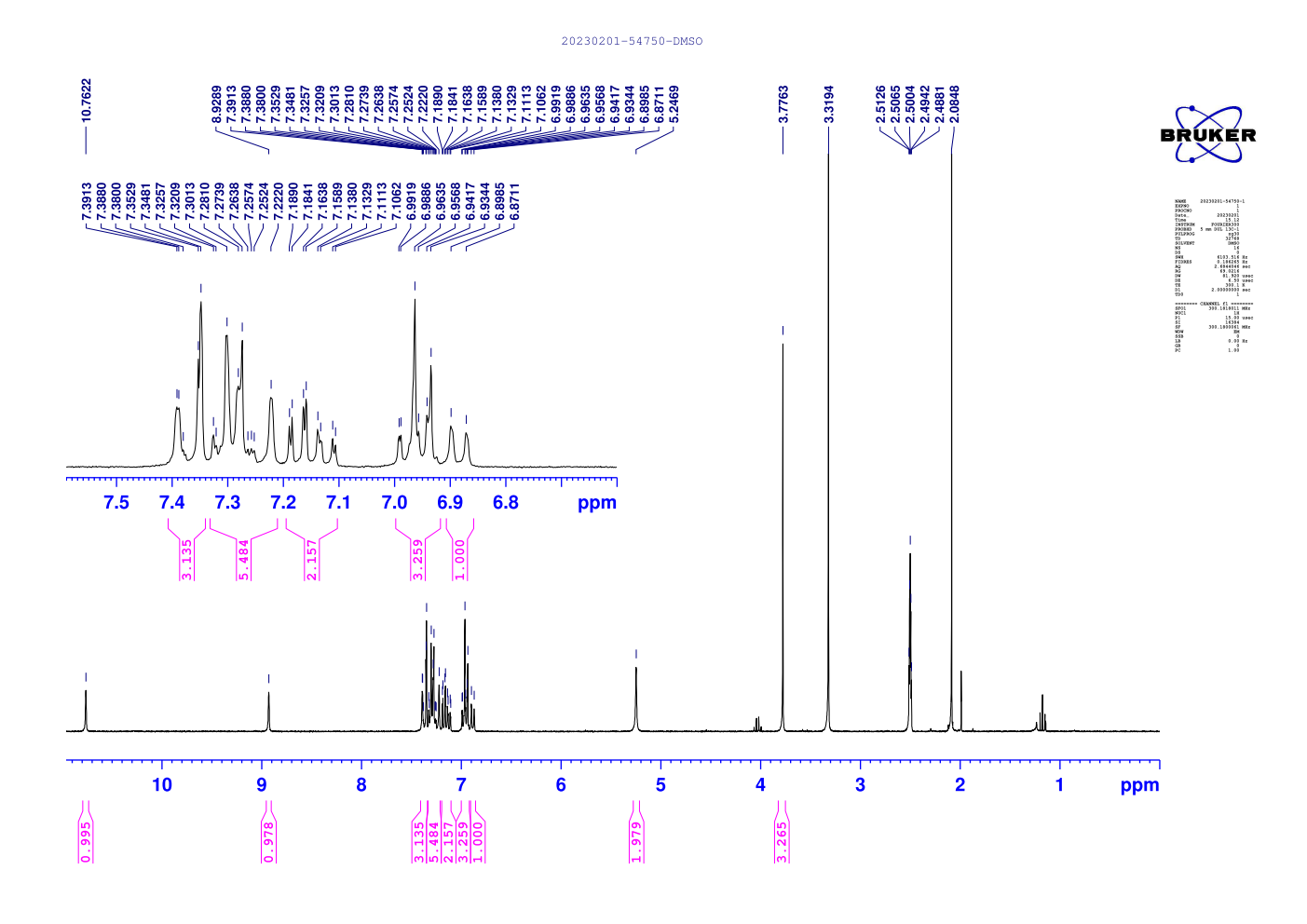


**Figure 14S.** ^1^H-NMR spectrum of compound **19b** (DMSO-*d_6_*, 300 MHz)


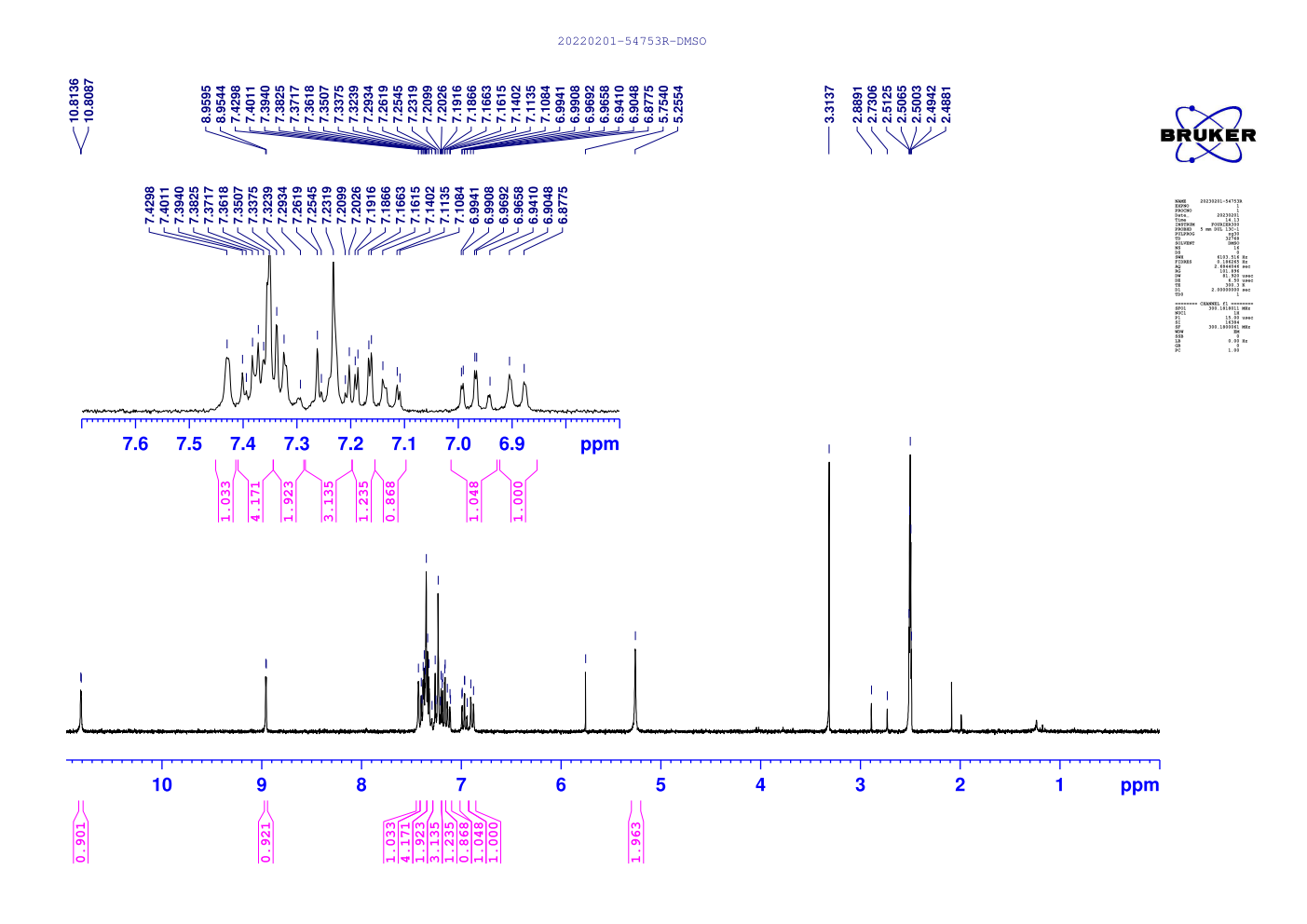


**Figure 15S.** ^1^H-NMR spectrum of compound **19c** (DMSO-*d_6_*, 300 MHz)


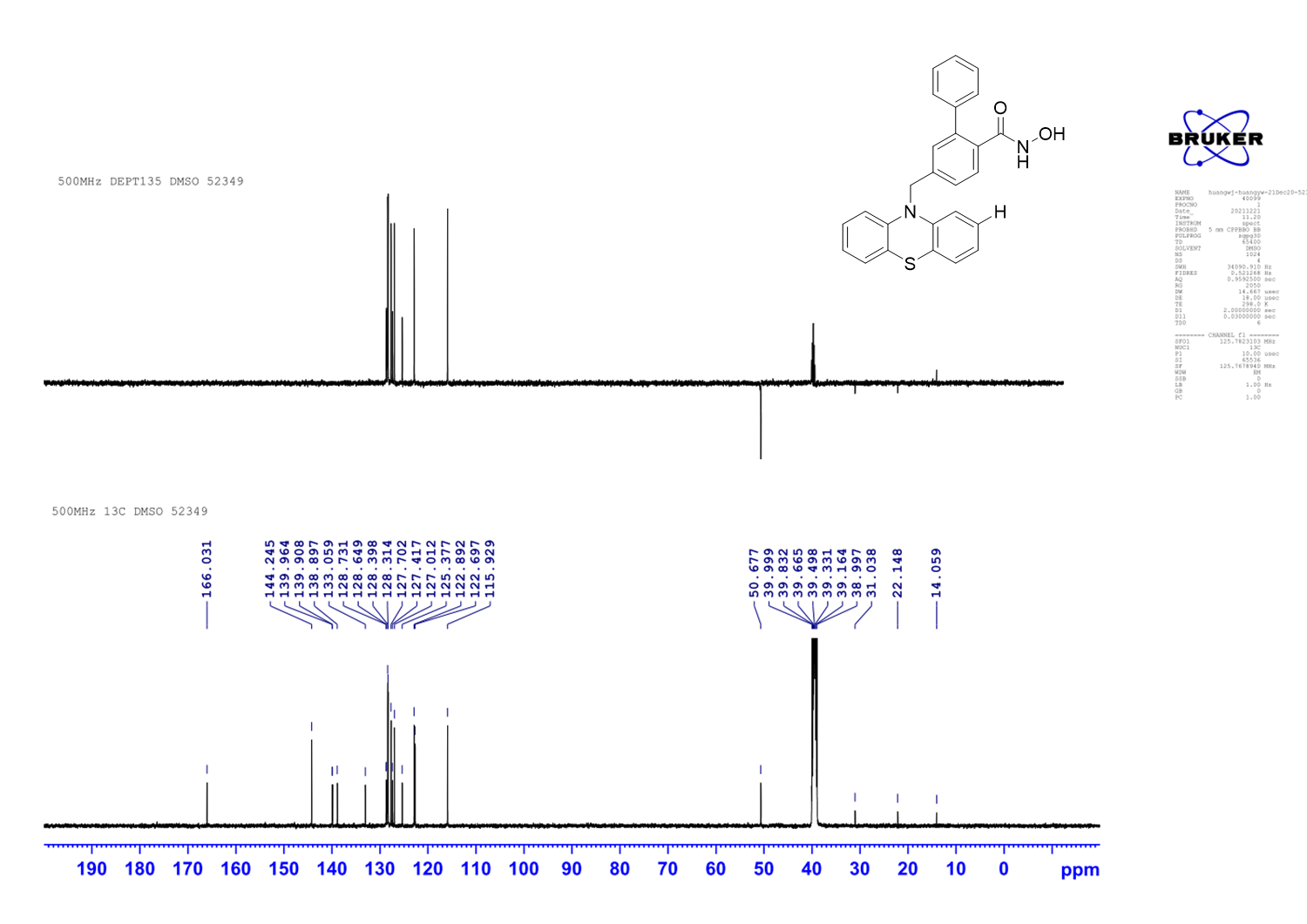


**Figure 16S.** ^13^C-NMR and DEPT 135 spectrum of compound **7a** (DMSO-*d_6_*, 125 MHz)


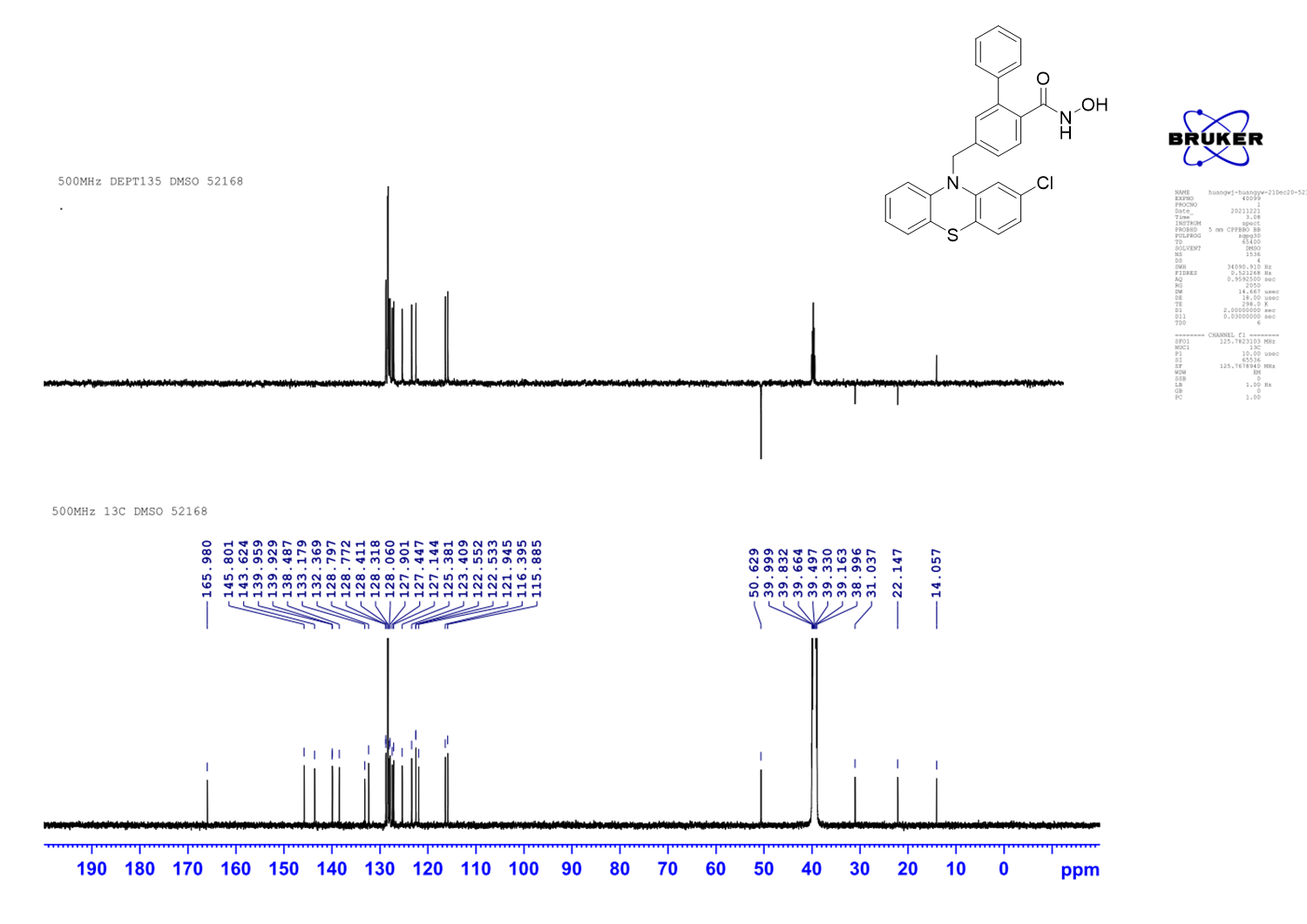


**Figure 17S.** ^13^C-NMR and DEPT 135 spectrum of compound **7b** (DMSO-*d_6_*, 125 MHz)


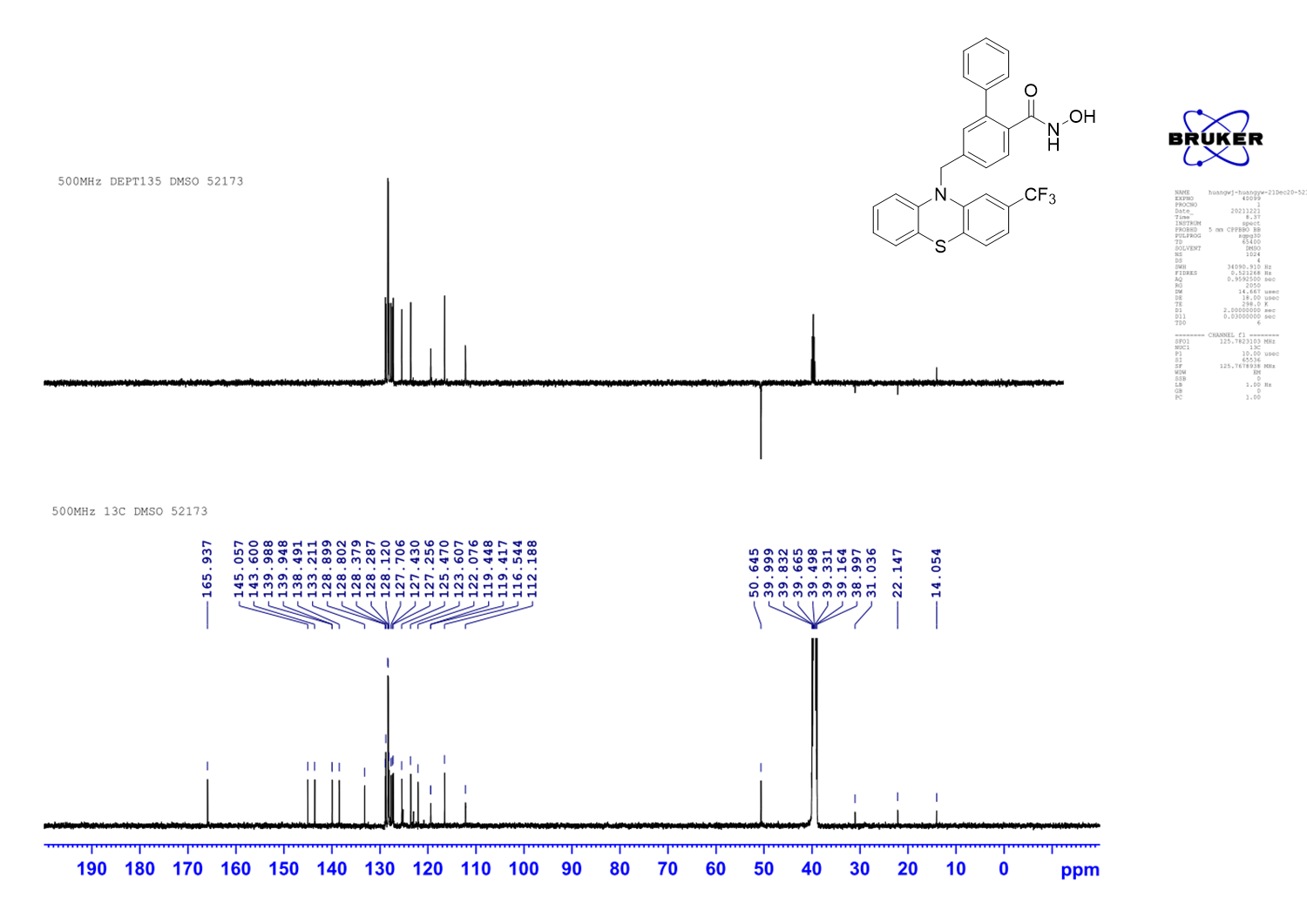


**Figure 18S.** ^13^C-NMR and DEPT 135 spectrum of compound **7c** (DMSO-*d_6_*, 125 MHz)


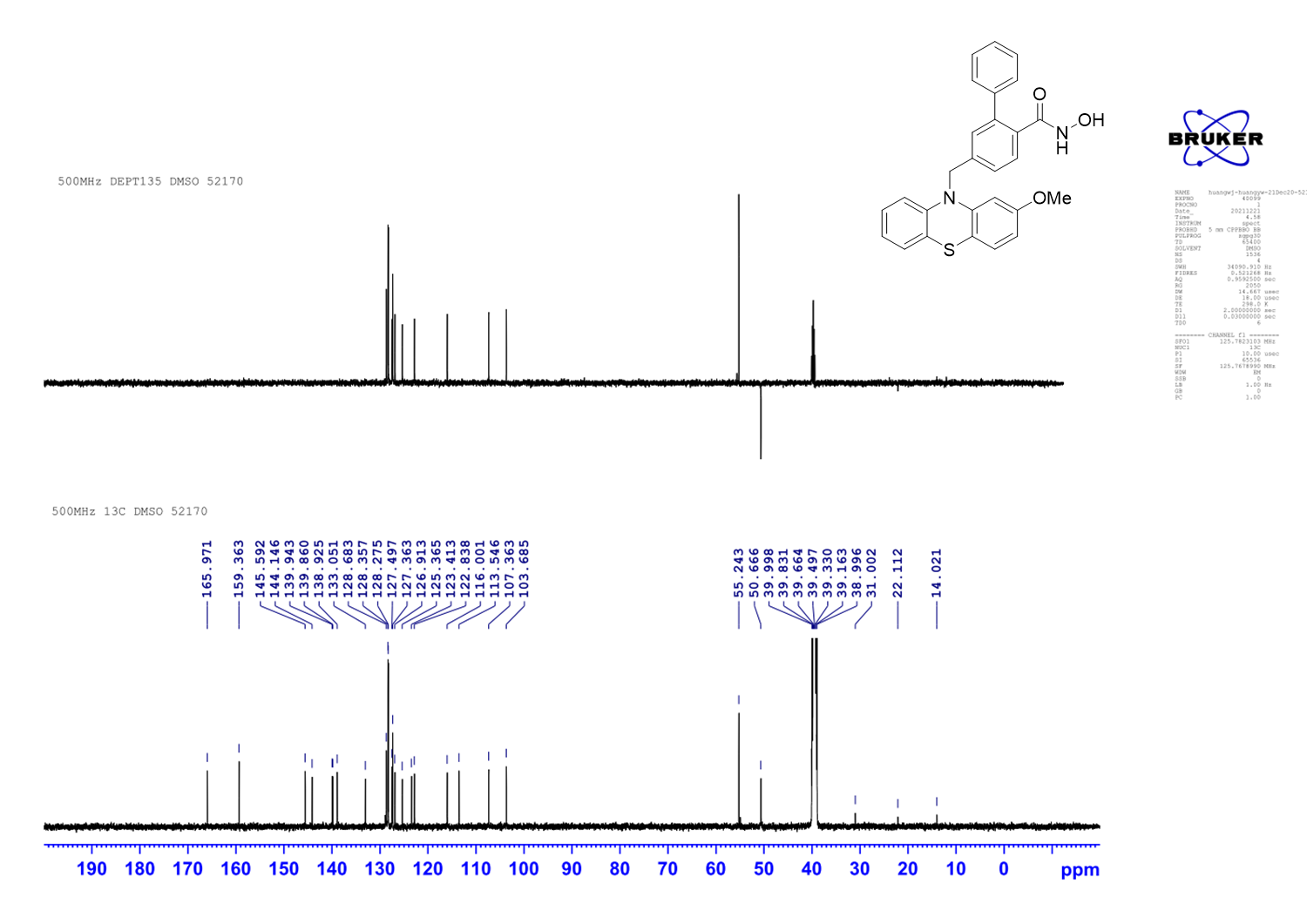


**Figure 19S.** ^13^C-NMR and DEPT 135 spectrum of compound **7d** (DMSO-*d_6_*, 125 MHz)


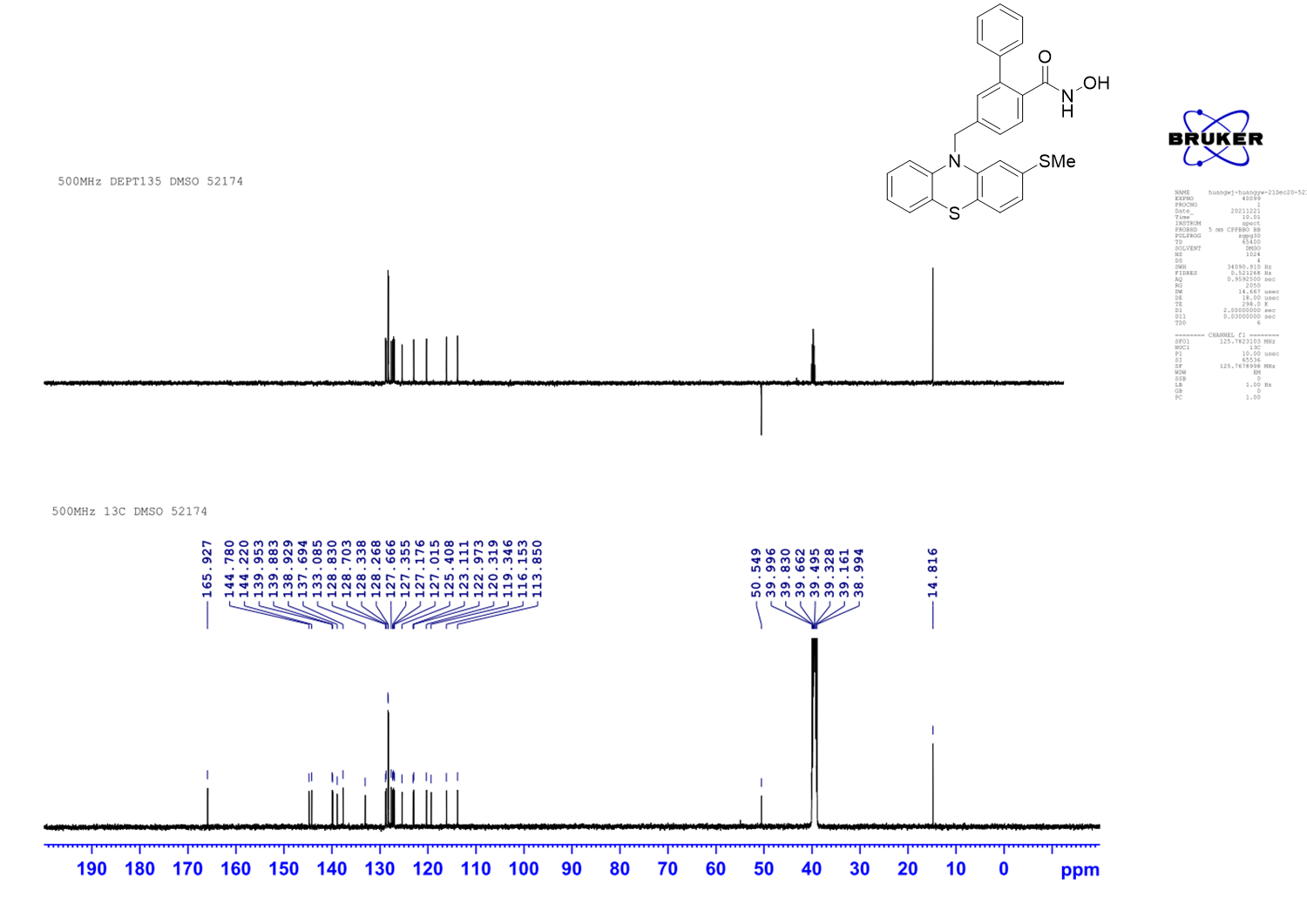


**Figure 20S.** ^13^C-NMR and DEPT 135 spectrum of compound **7e** (DMSO-*d_6_*, 125 MHz)


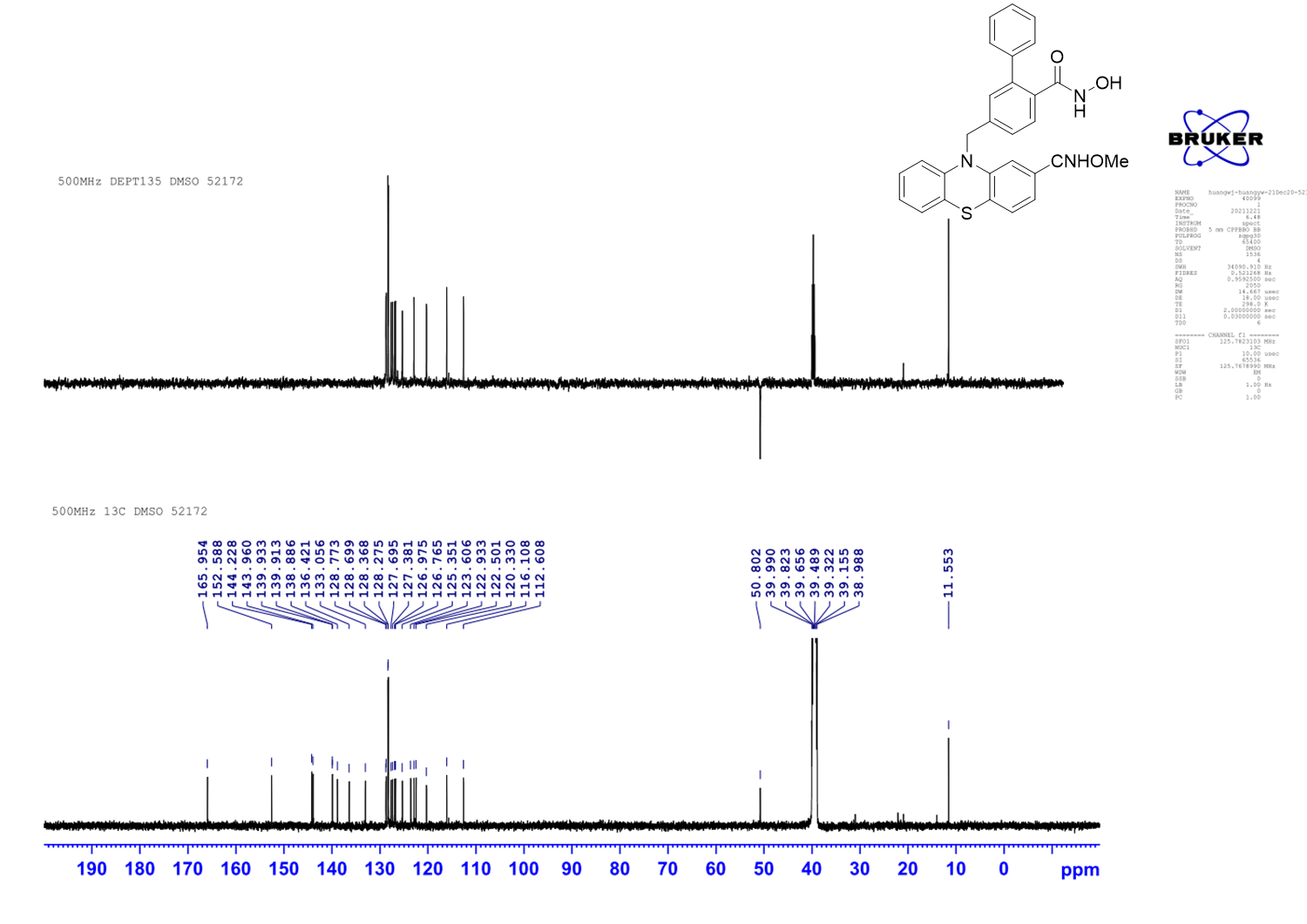


**Figure 21S.** ^13^C-NMR and DEPT 135 spectrum of compound **7f** (DMSO-*d_6_*, 125 MHz)


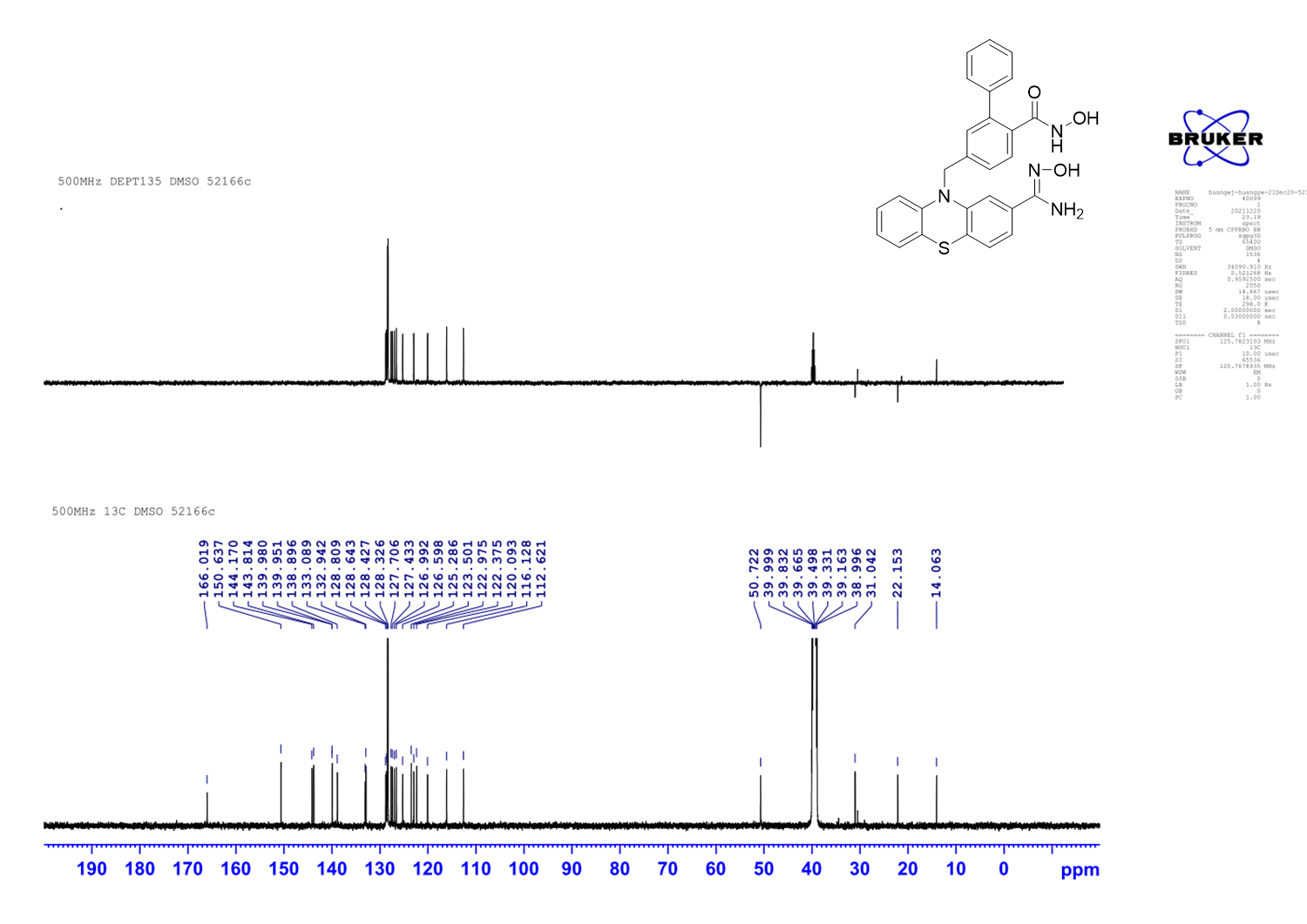


**Figure 22S.** ^13^C-NMR and DEPT 135 spectrum of compound **7g** (DMSO-*d_6_*, 125 MHz)


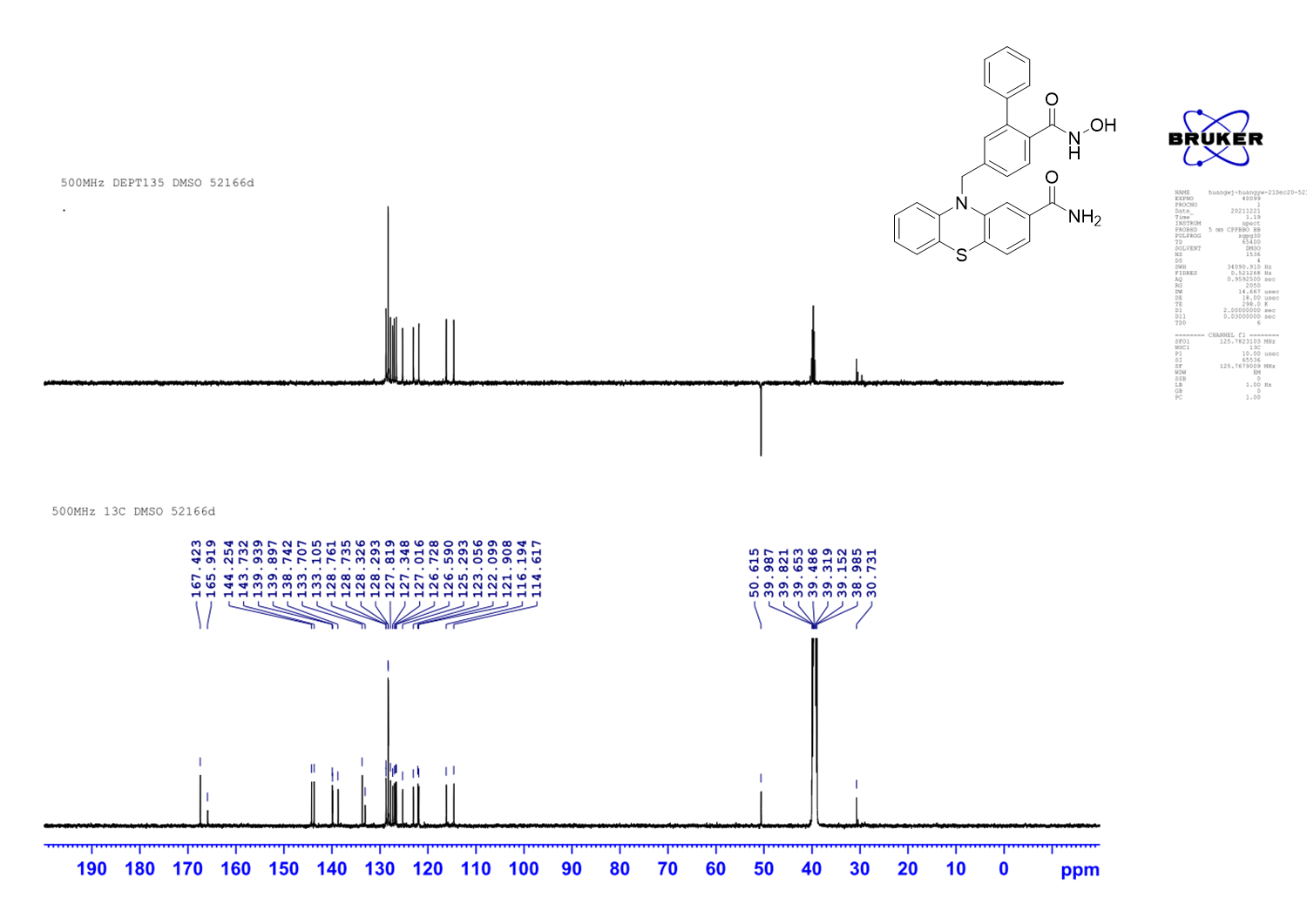


**Figure 23S.** ^13^C-NMR and DEPT 135 spectrum of compound **7h** (DMSO-*d_6_*, 125 MHz)


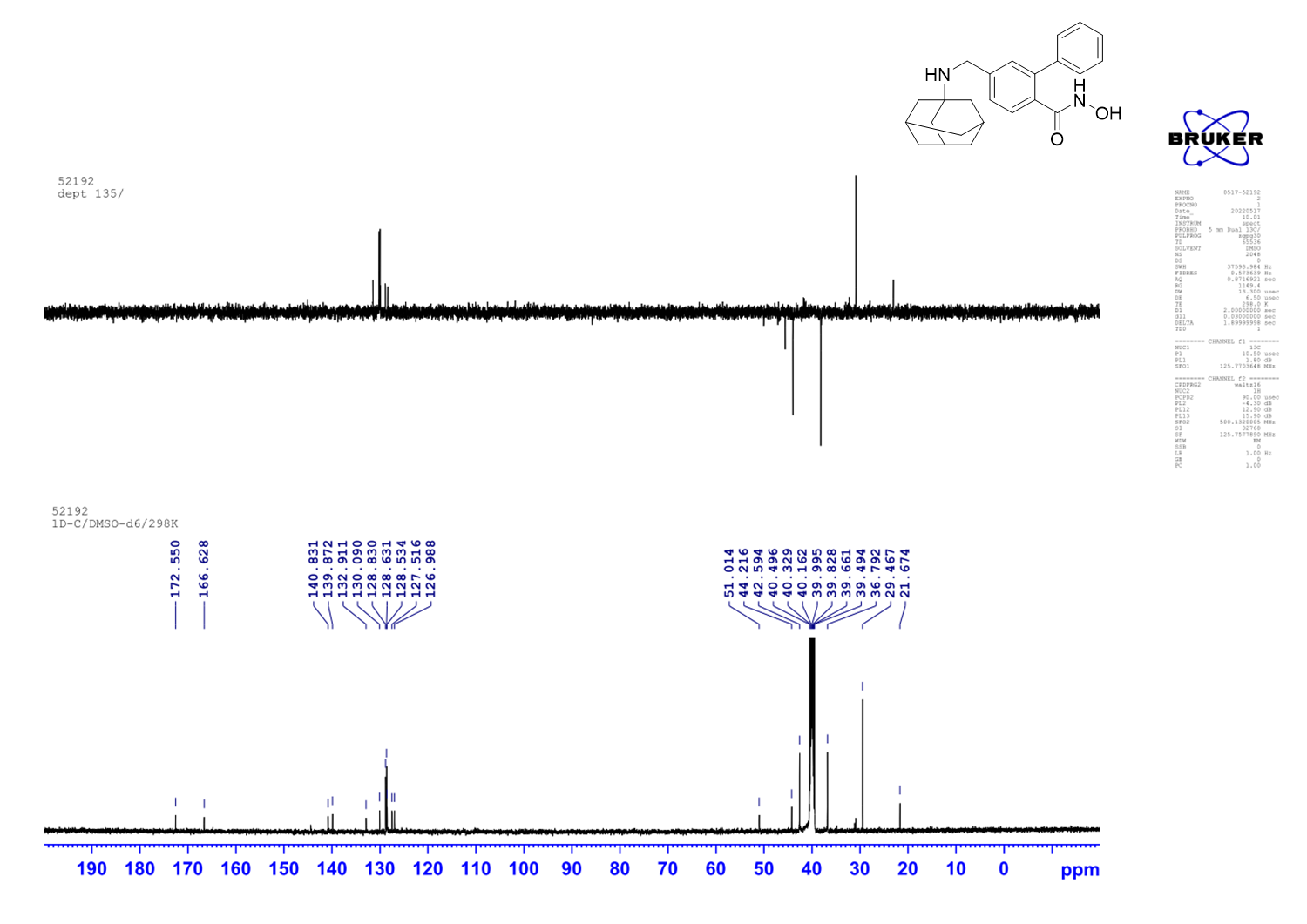


**Figure 24S.** ^13^C-NMR and DEPT 135 spectrum of compound **7i** (DMSO-*d_6_*, 125 MHz)


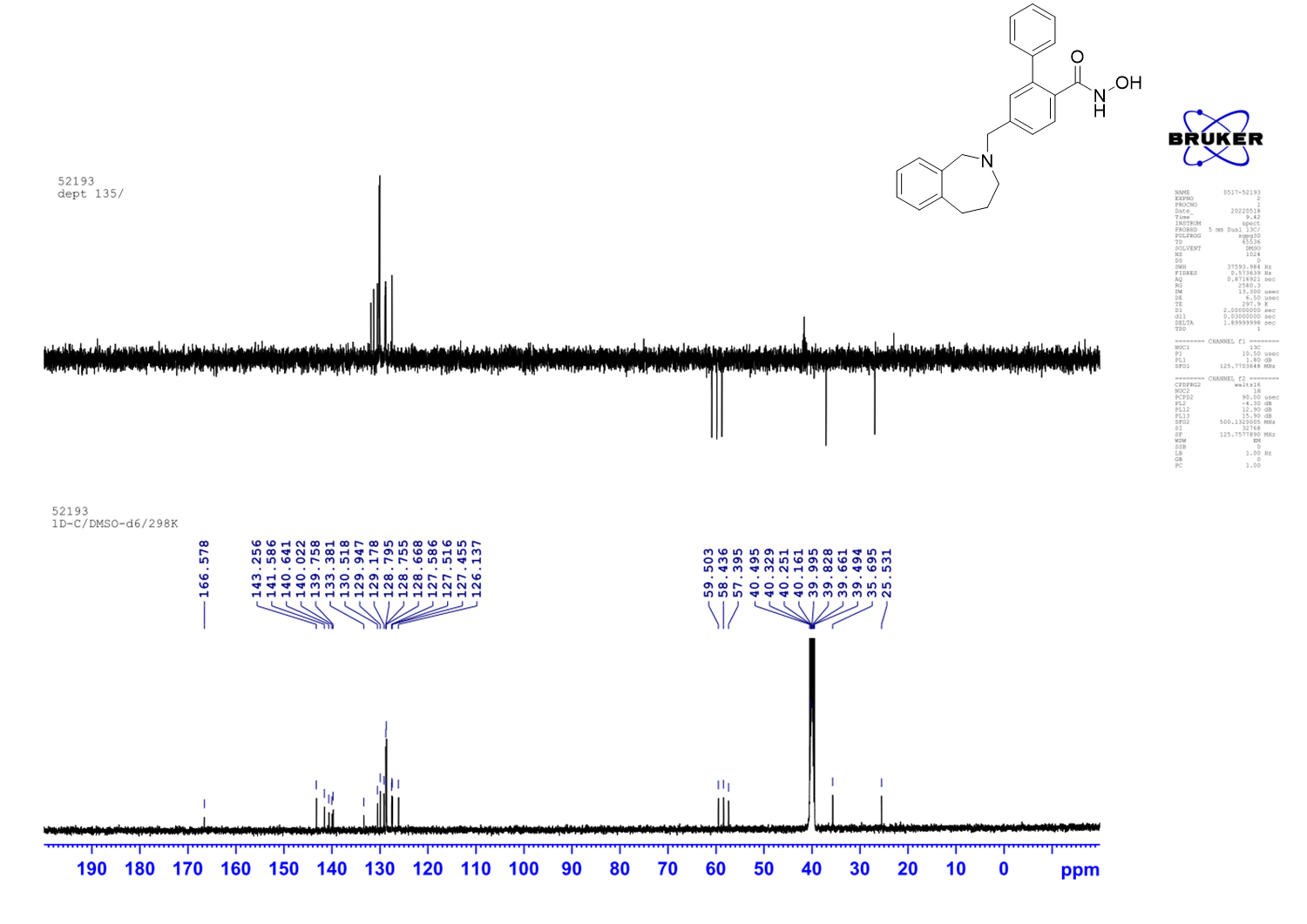


**Figure 25S.** ^13^C-NMR and DEPT 135 spectrum of compound **7j** (DMSO-*d_6_*, 125 MHz)


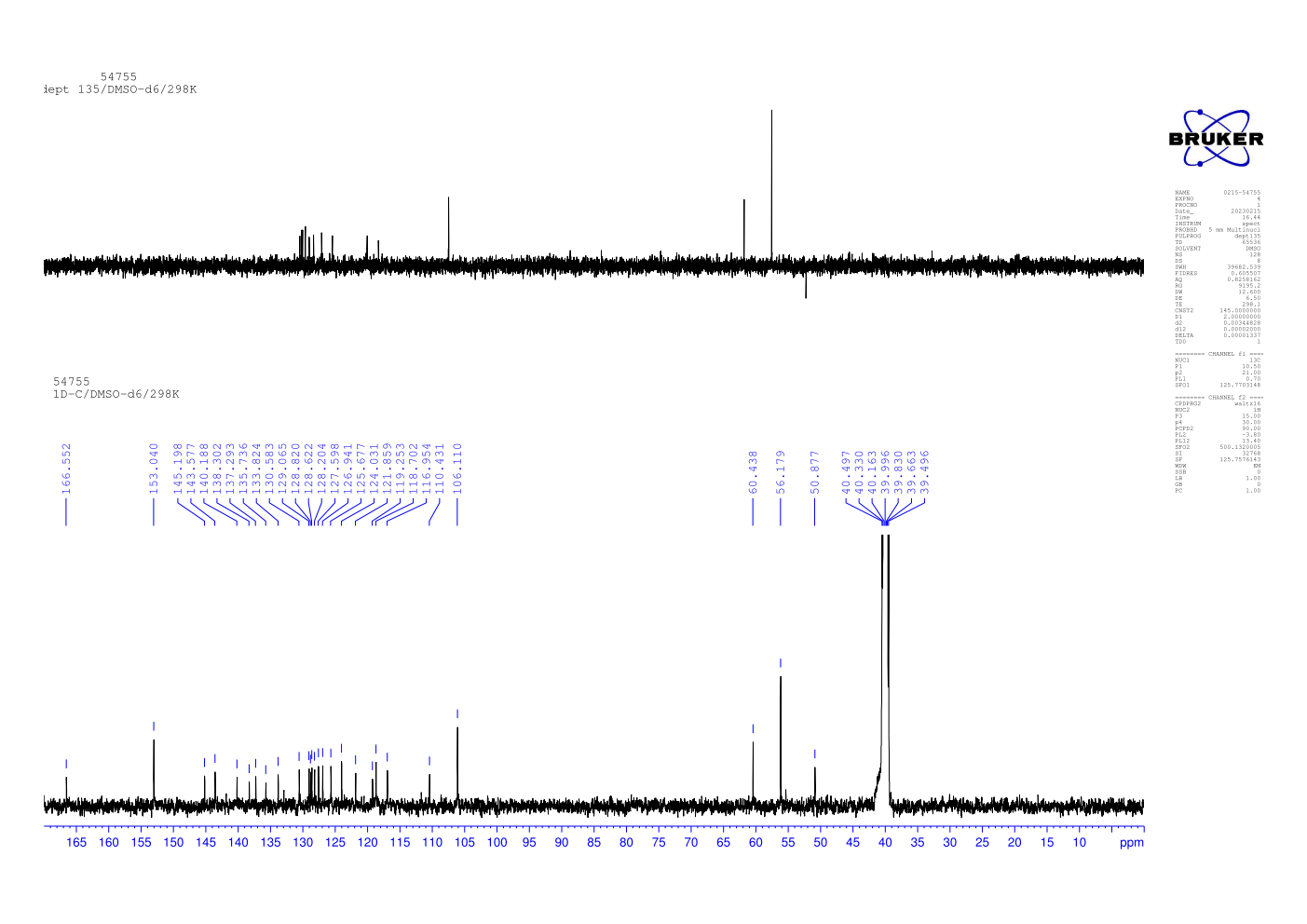


**Figure 26S.** ^13^C-NMR and DEPT 135 spectrum of compound **19a** (DMSO-*d_6_*, 125 MHz)


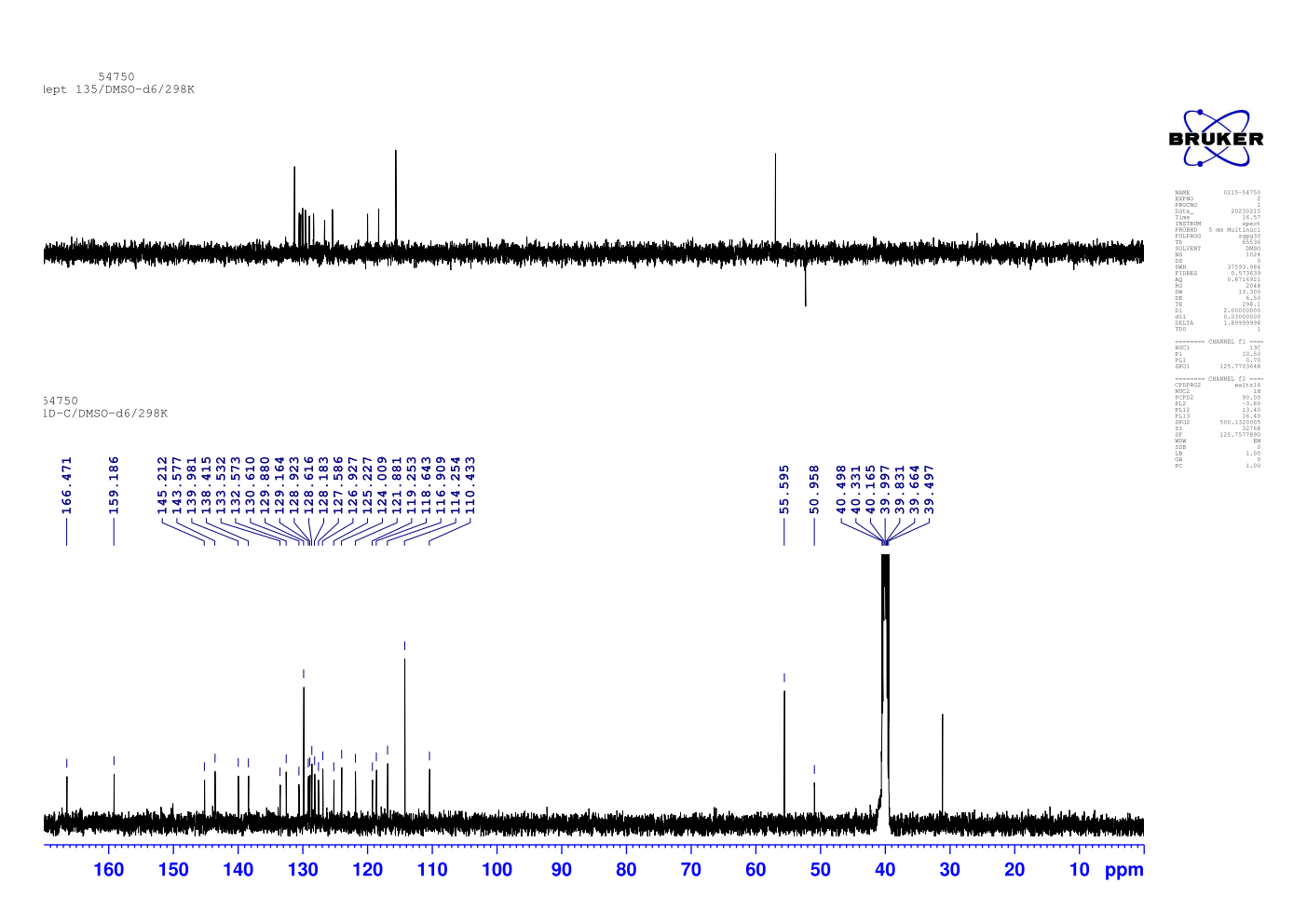


**Figure 27S.** ^13^C-NMR and DEPT 135 spectrum of compound **19b** (DMSO-*d_6_*, 125 MHz)


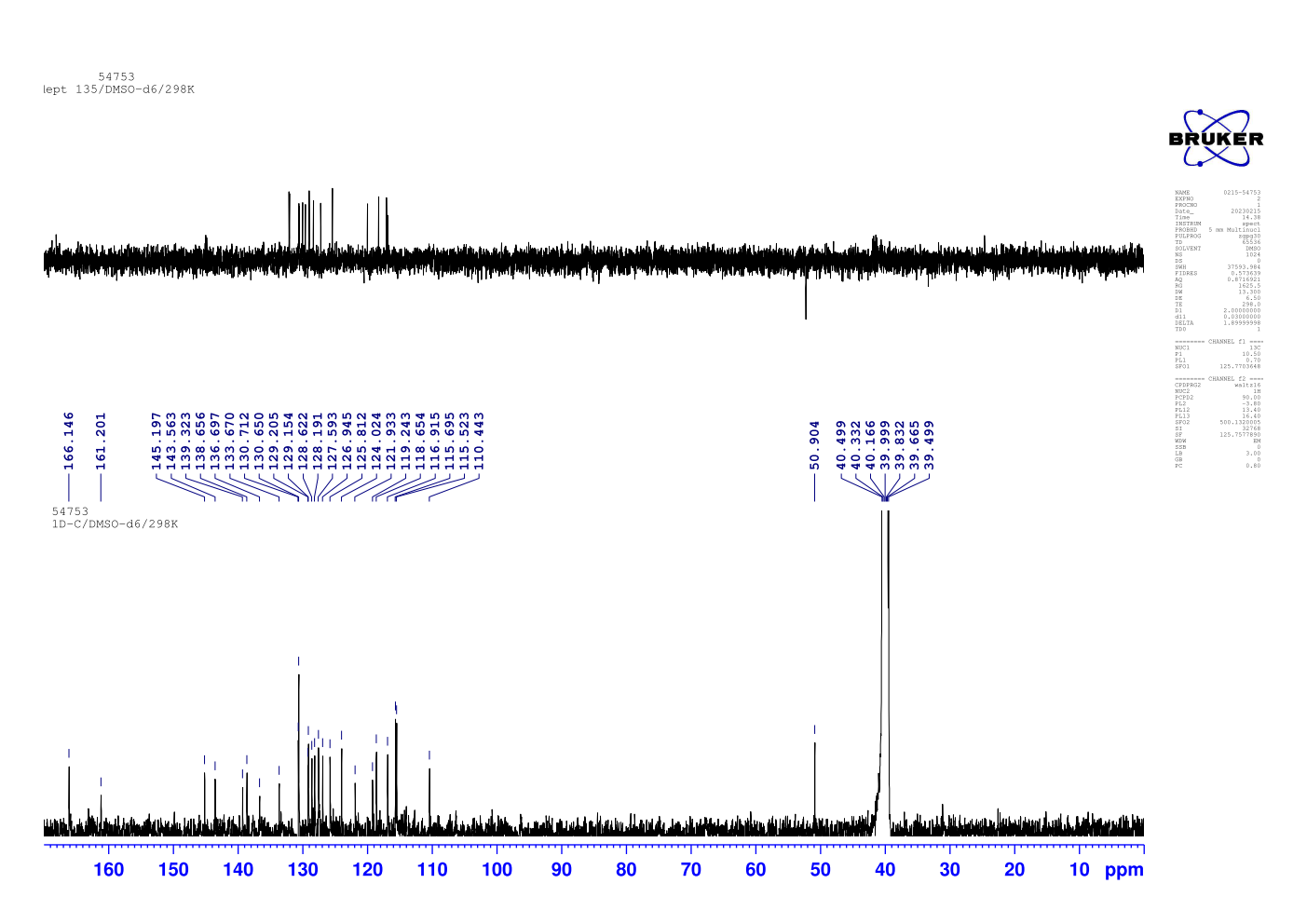


**Figure 28S.** ^13^C-NMR and DEPT 135 spectrum of compound **19c** (DMSO-*d_6_*, 125 MHz)


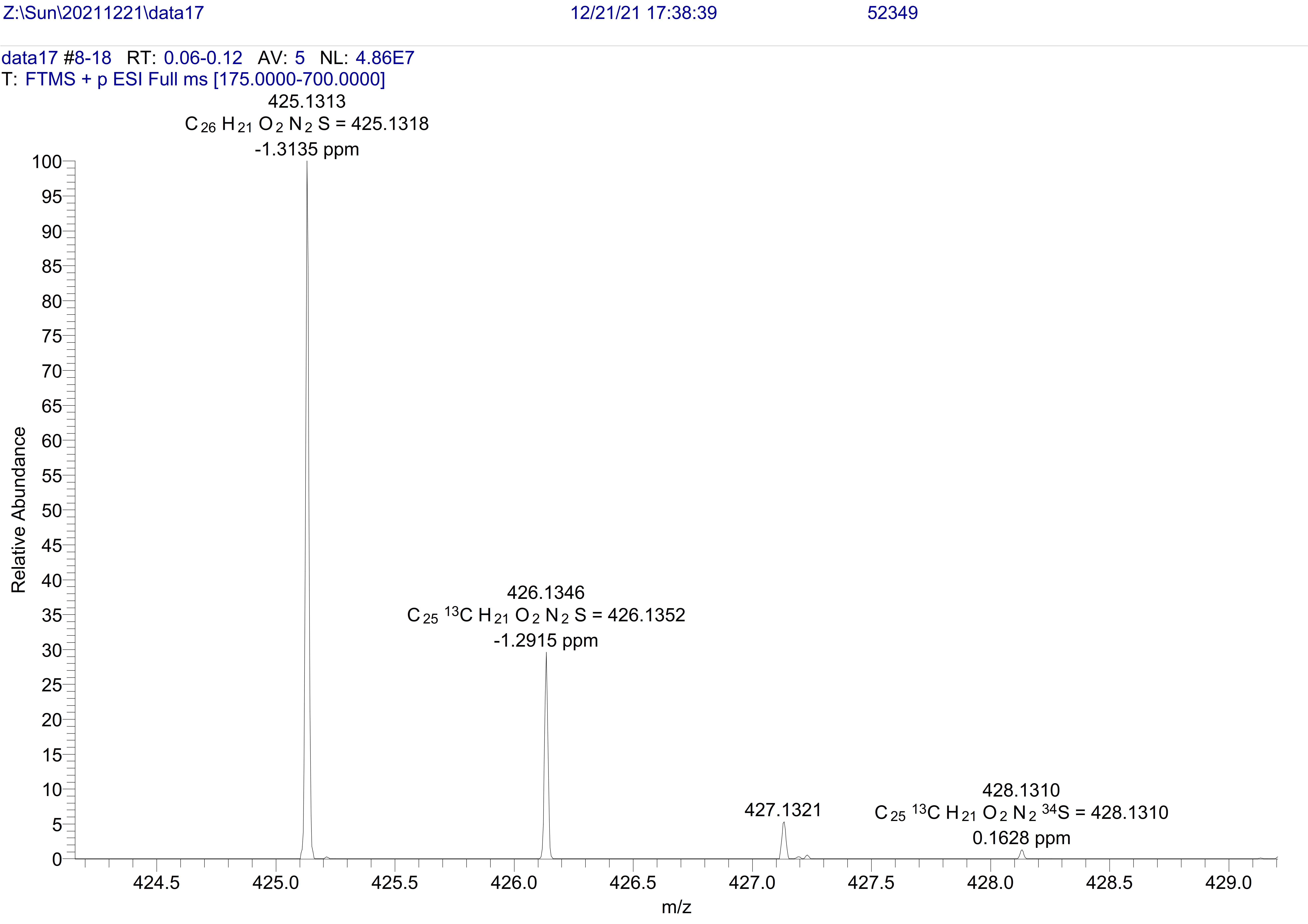


**Figure 29S.** High resolution ESI-MS spectrum of compound **7a**


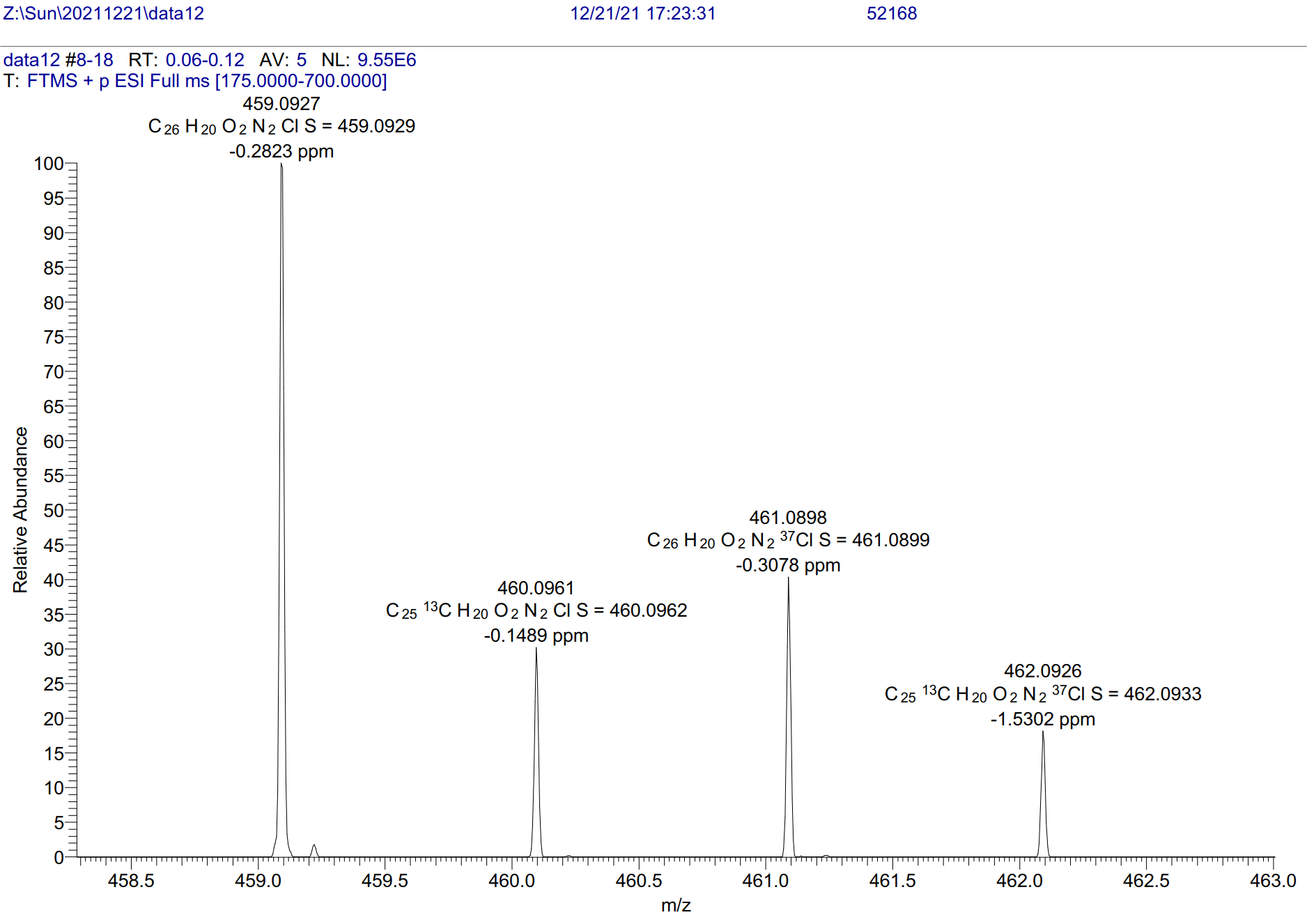


**Figure 30S.** High resolution ESI-MS spectrum of compound **7b**


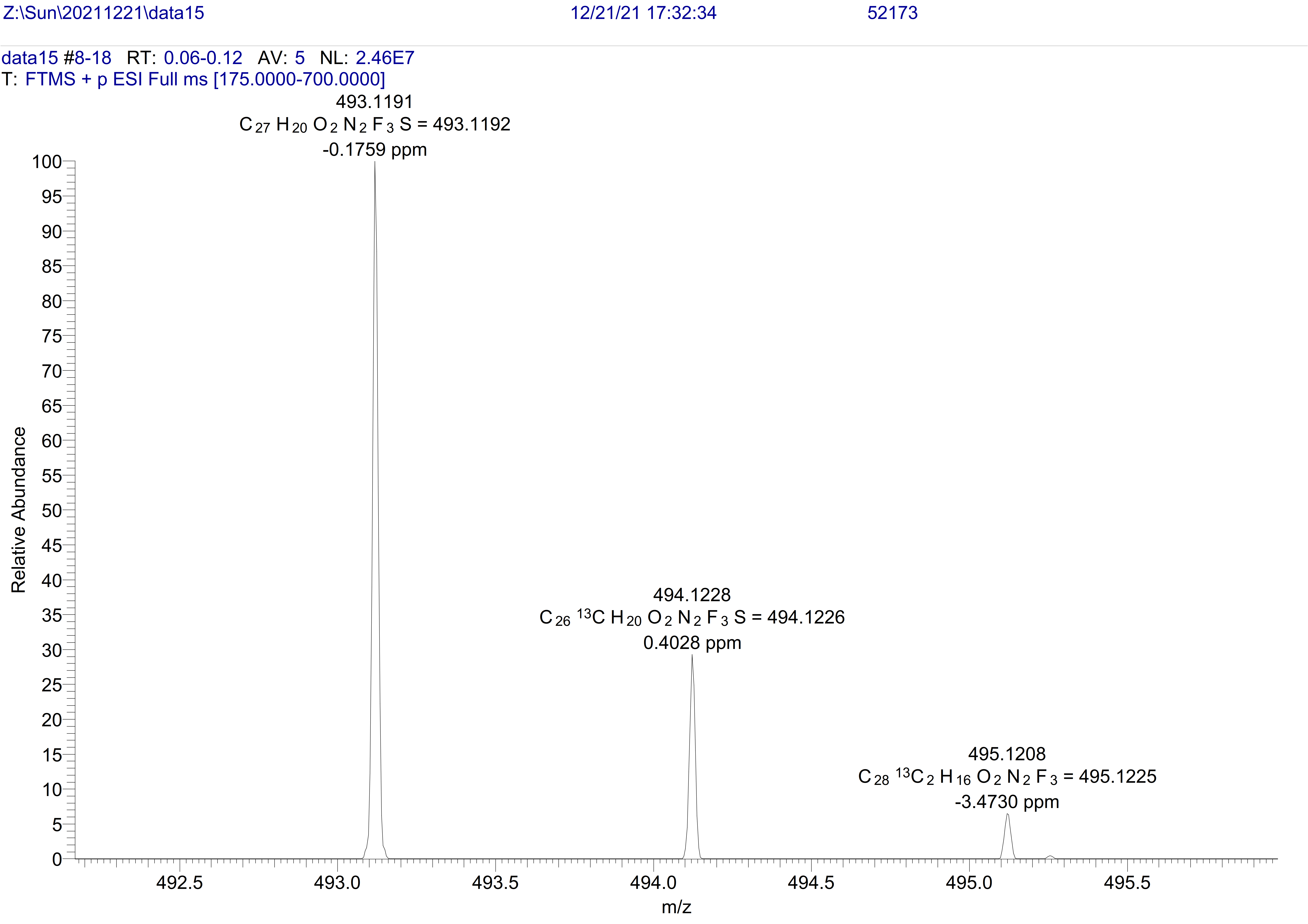


**Figure 31S.** High resolution ESI-MS spectrum of compound **7c**


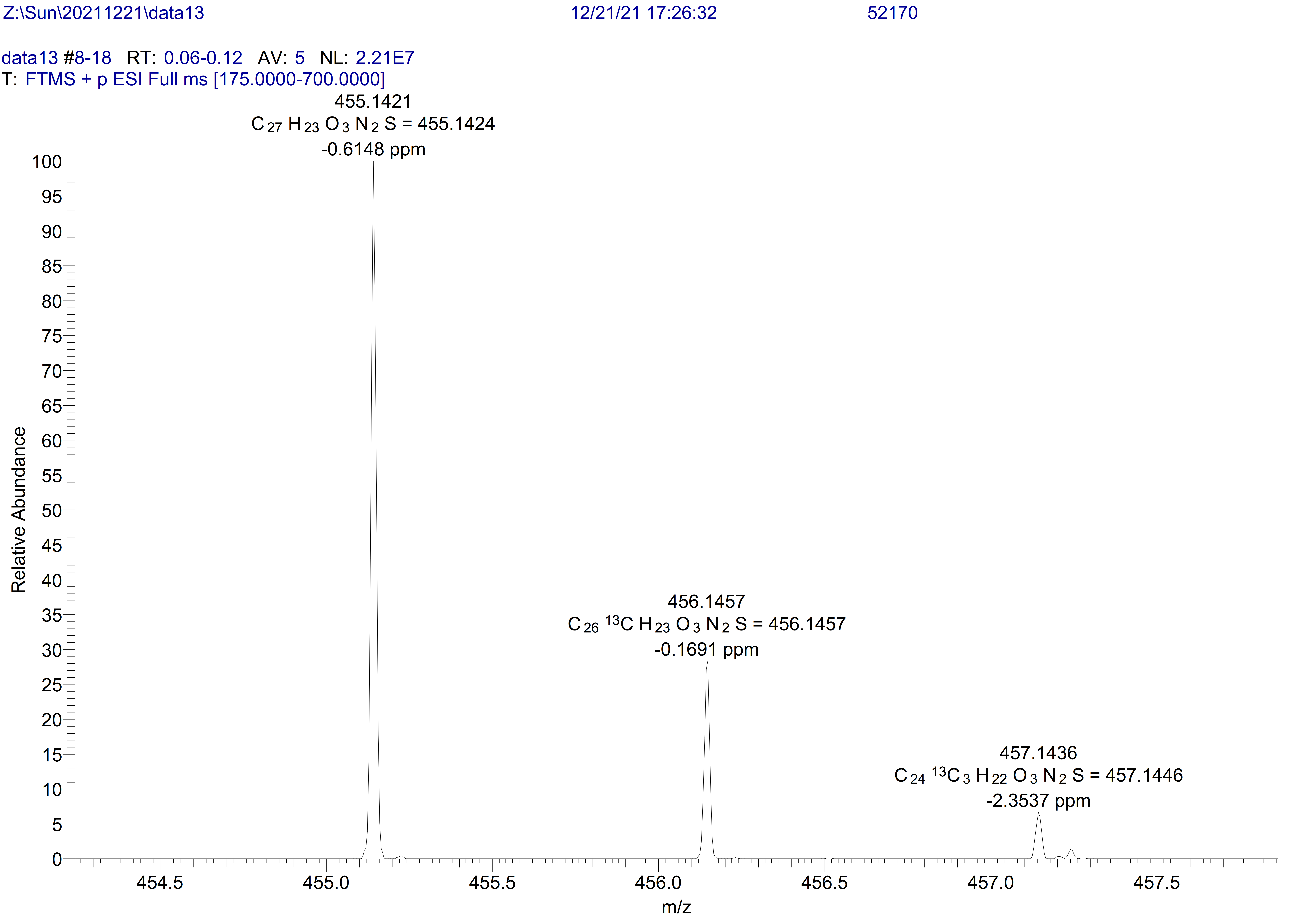


**Figure 32S.** High resolution ESI-MS spectrum of compound **7d**


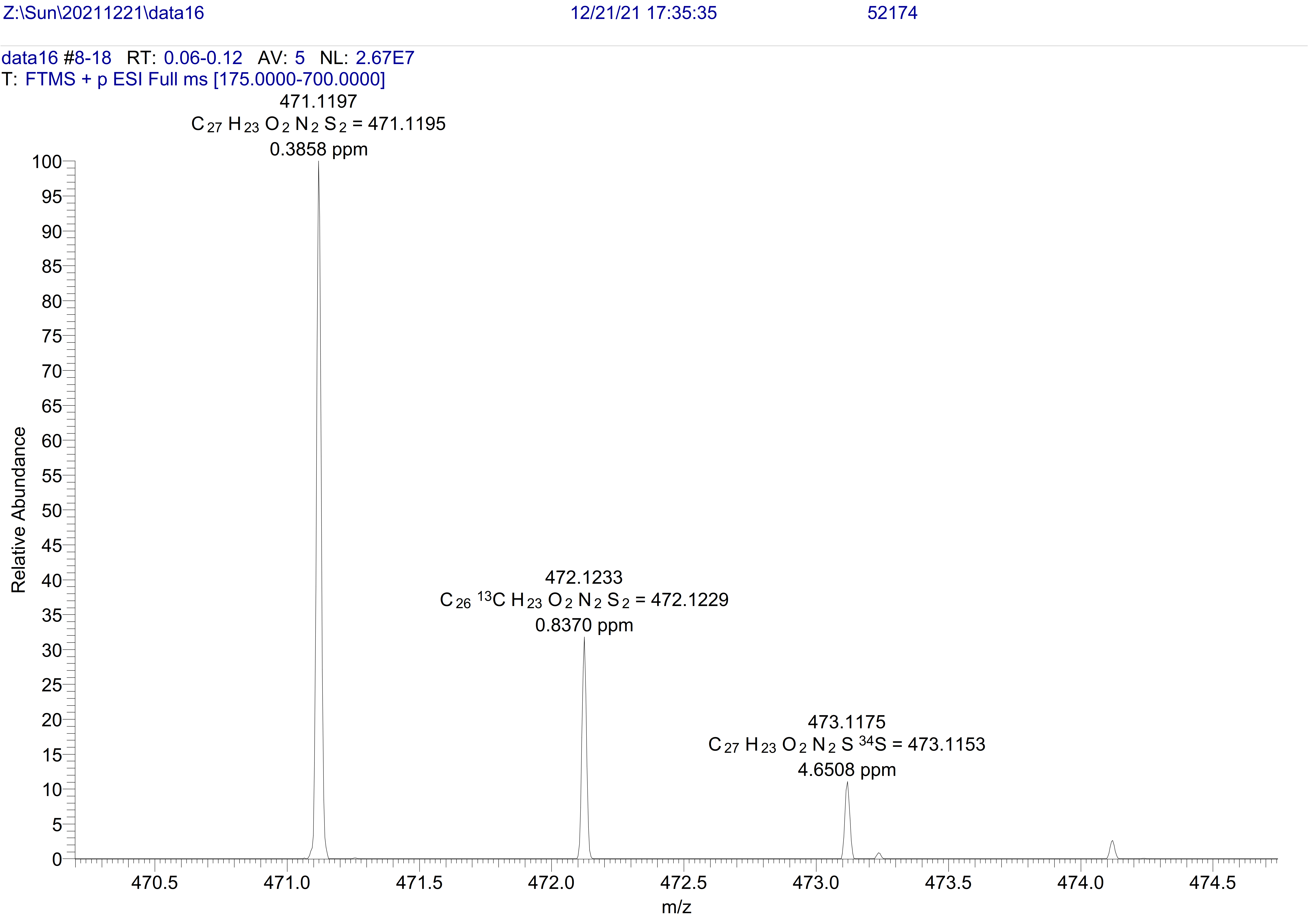


**Figure 33S.** High resolution ESI-MS spectrum of compound **7e**


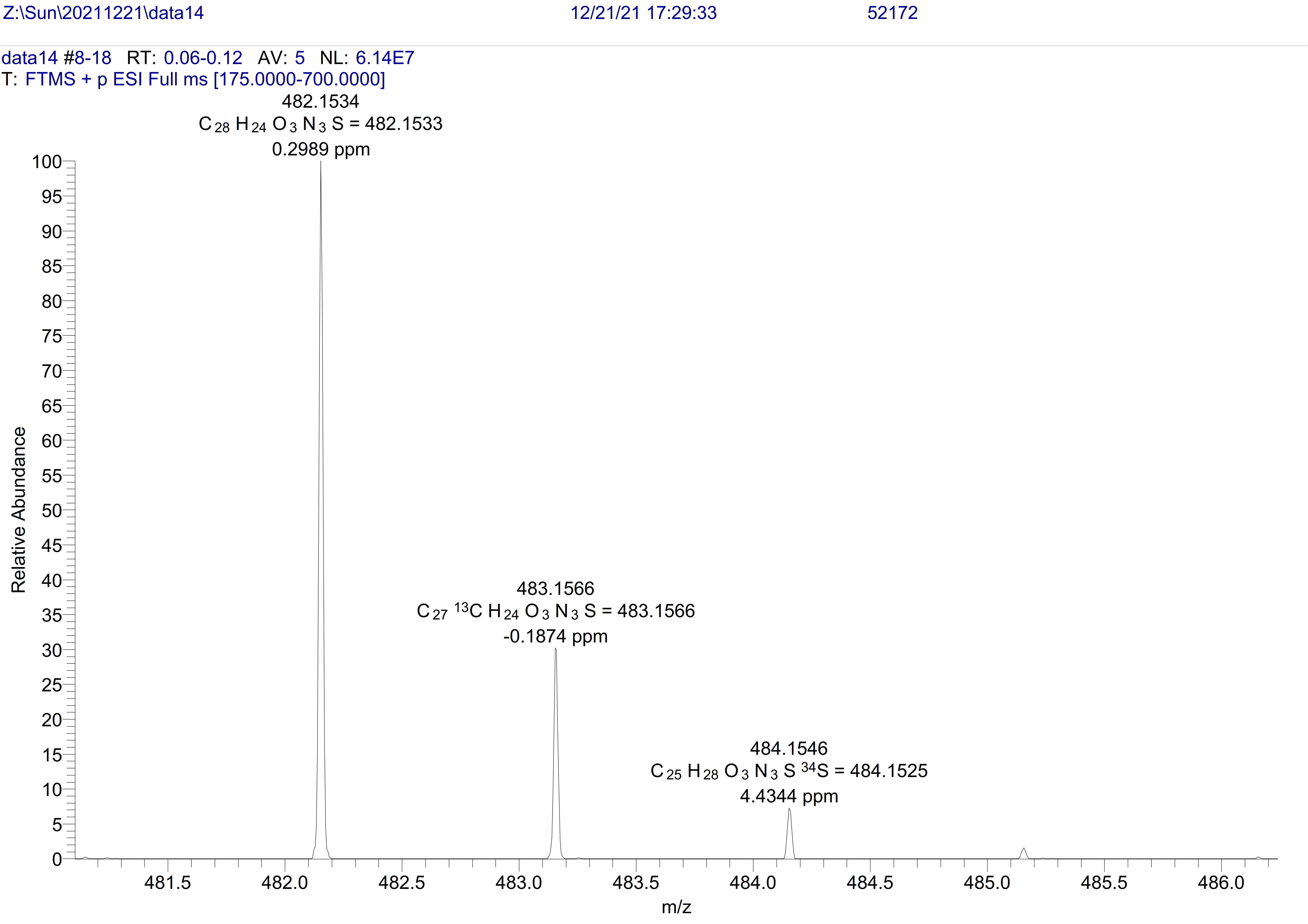


**Figure 34S.** High resolution ESI-MS spectrum of compound **7f**


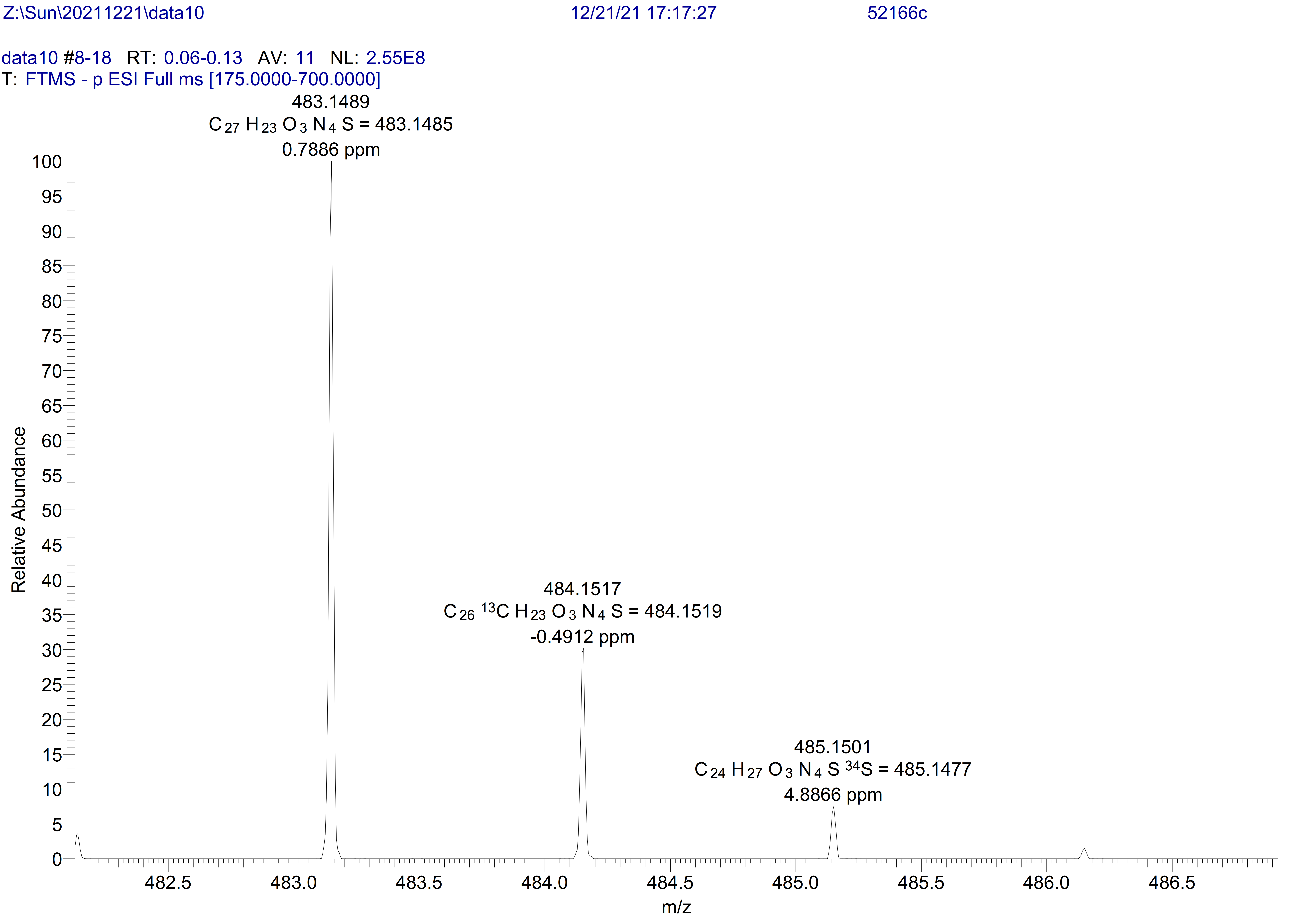


**Figure 35S.** High resolution ESI-MS spectrum of compound **7g**


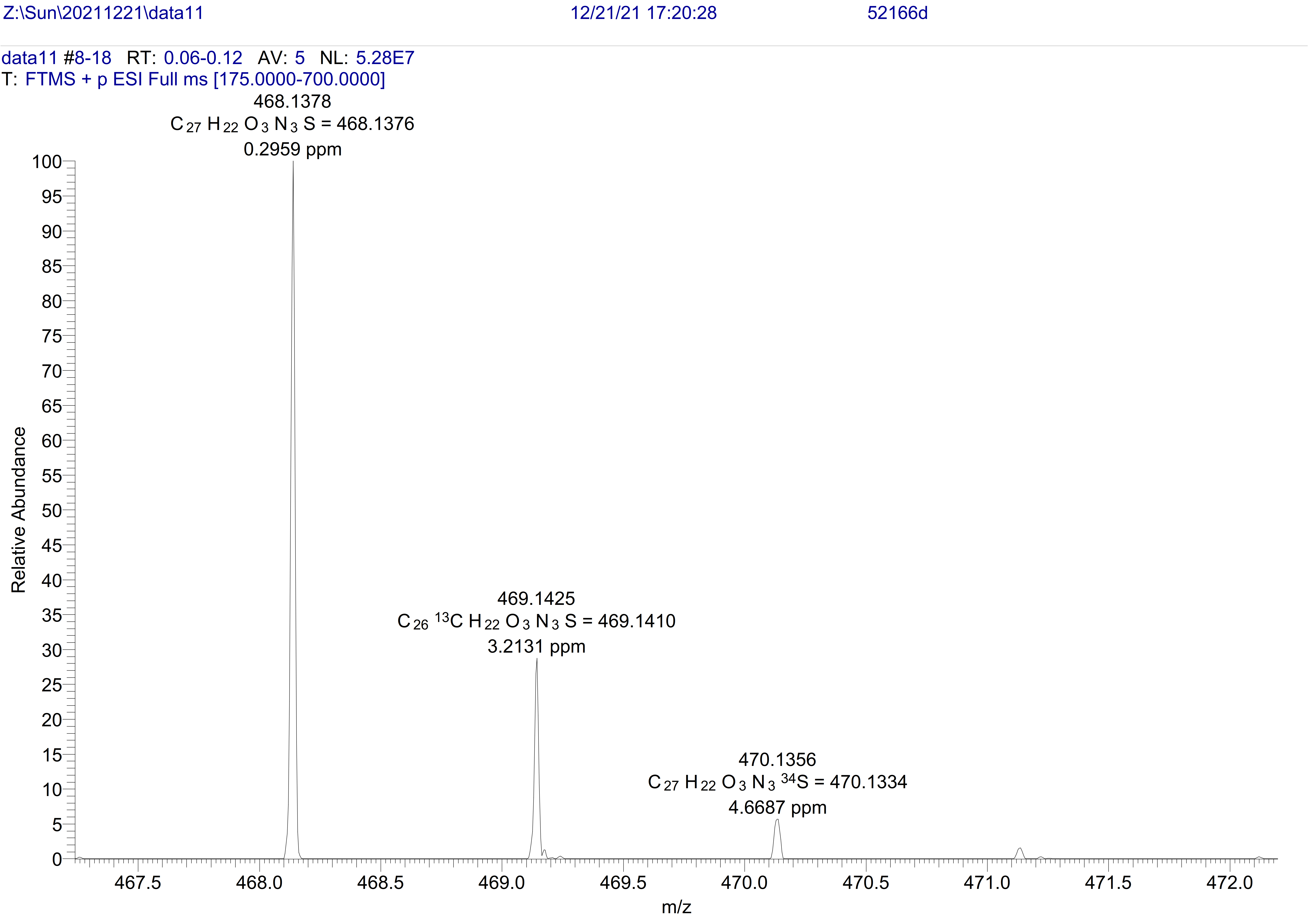


**Figure 36S.** High resolution ESI-MS spectrum of compound **7h**


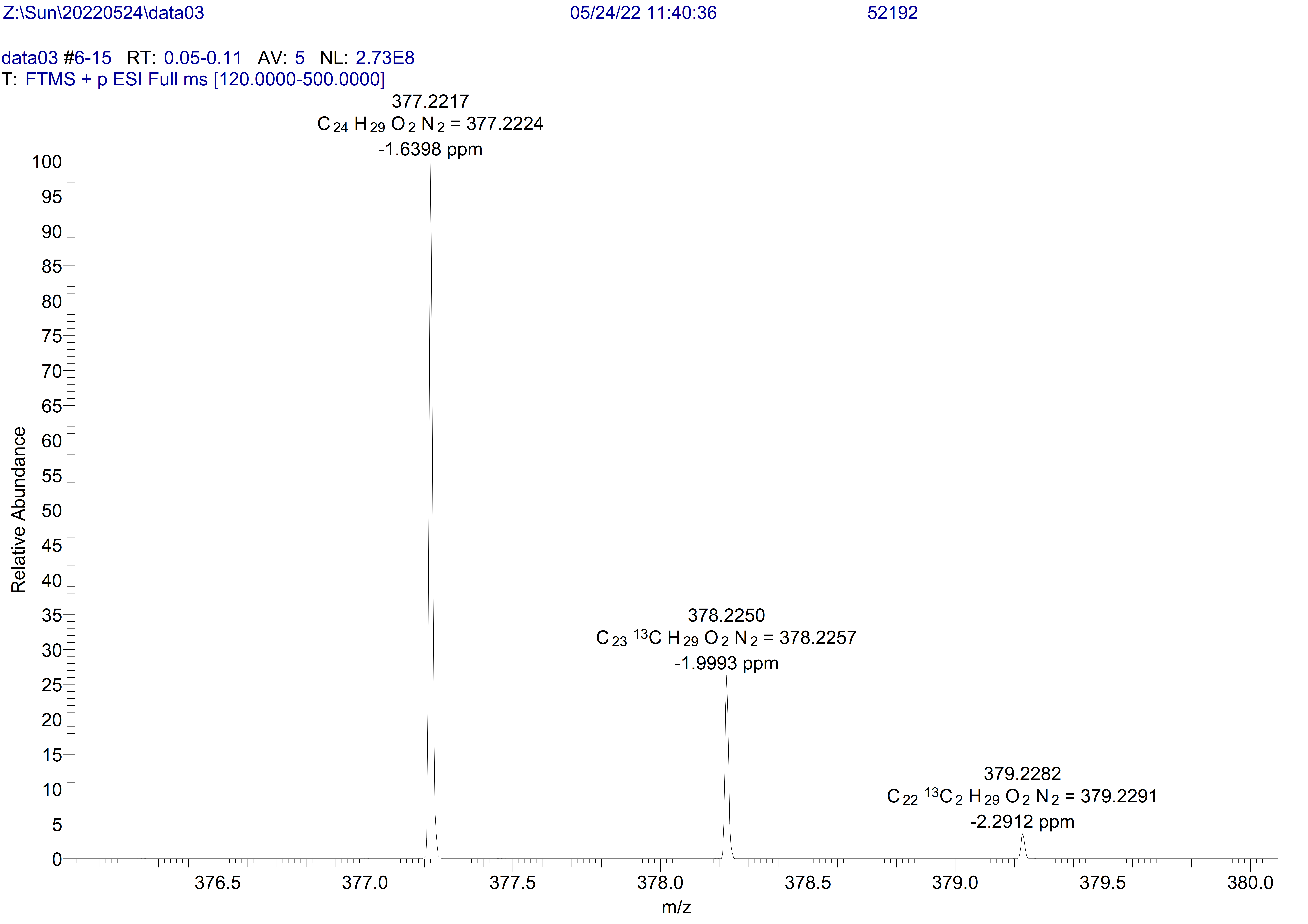


**Figure 37S.** High resolution ESI-MS spectrum of compound **7i**


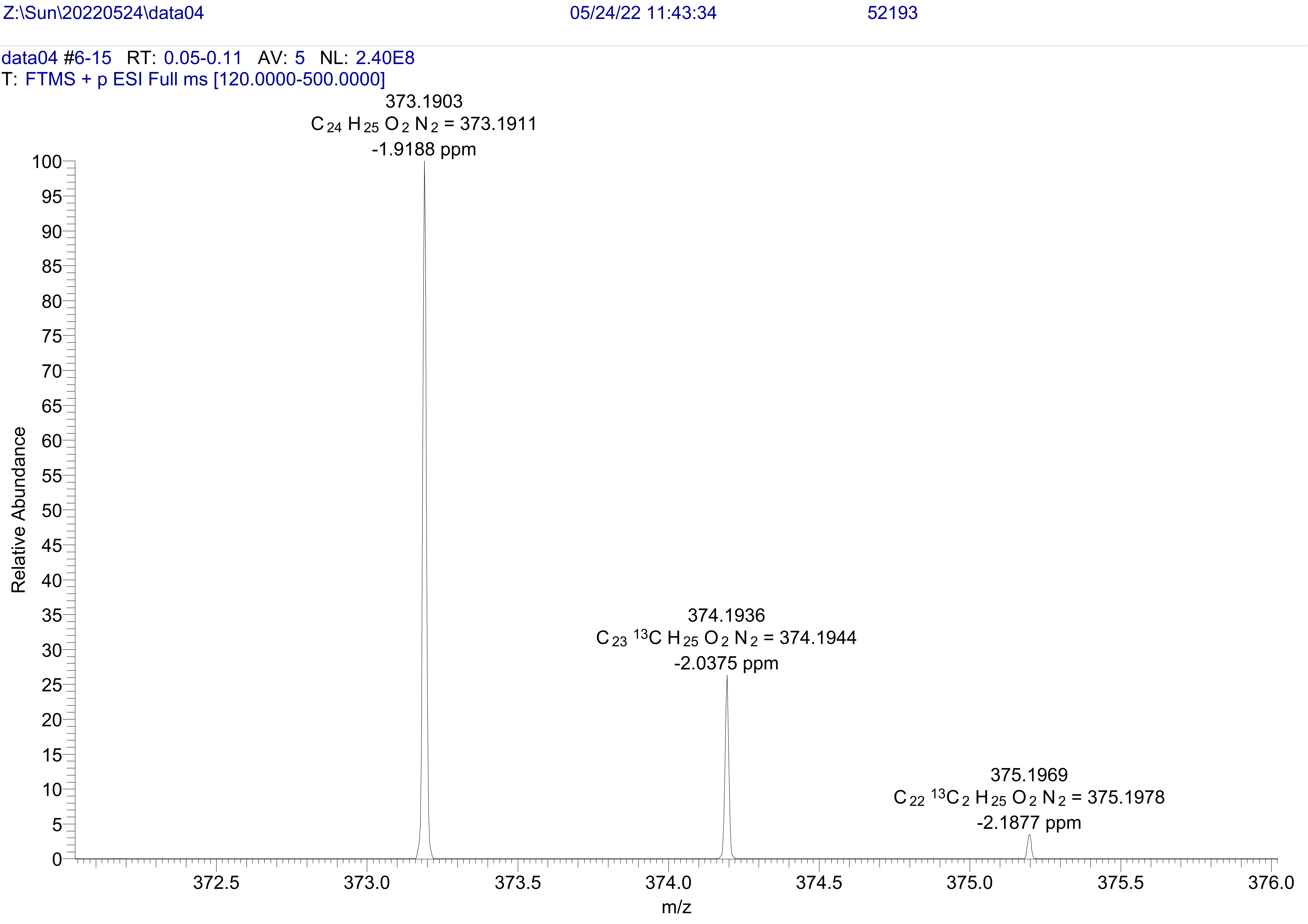


**Figure 38S.** High resolution ESI-MS spectrum of compound **7j**


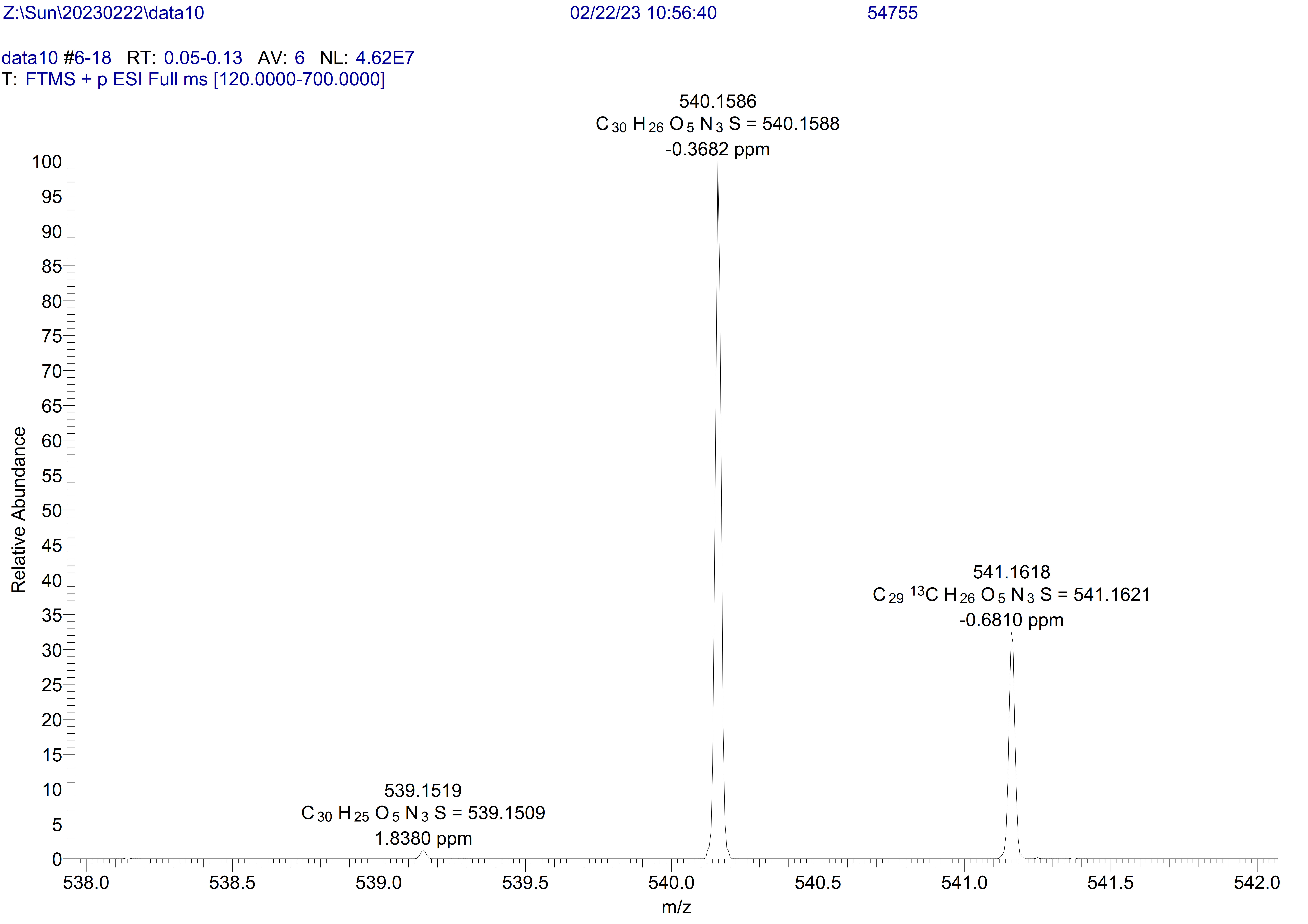


**Figure 39S.** High resolution ESI-MS spectrum of compound **19a**


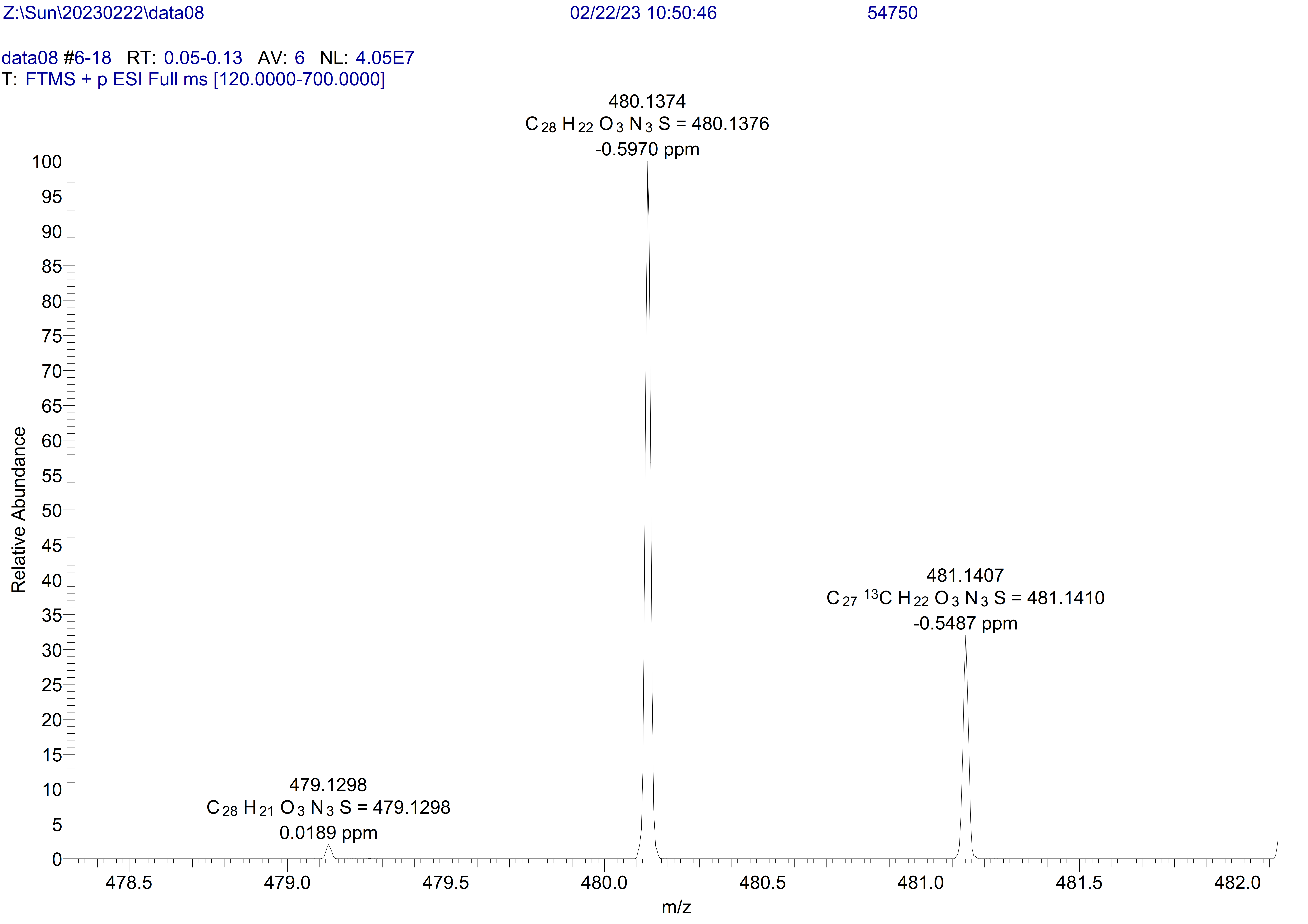


**Figure 40S.** High resolution ESI-MS spectrum of compound **19b**


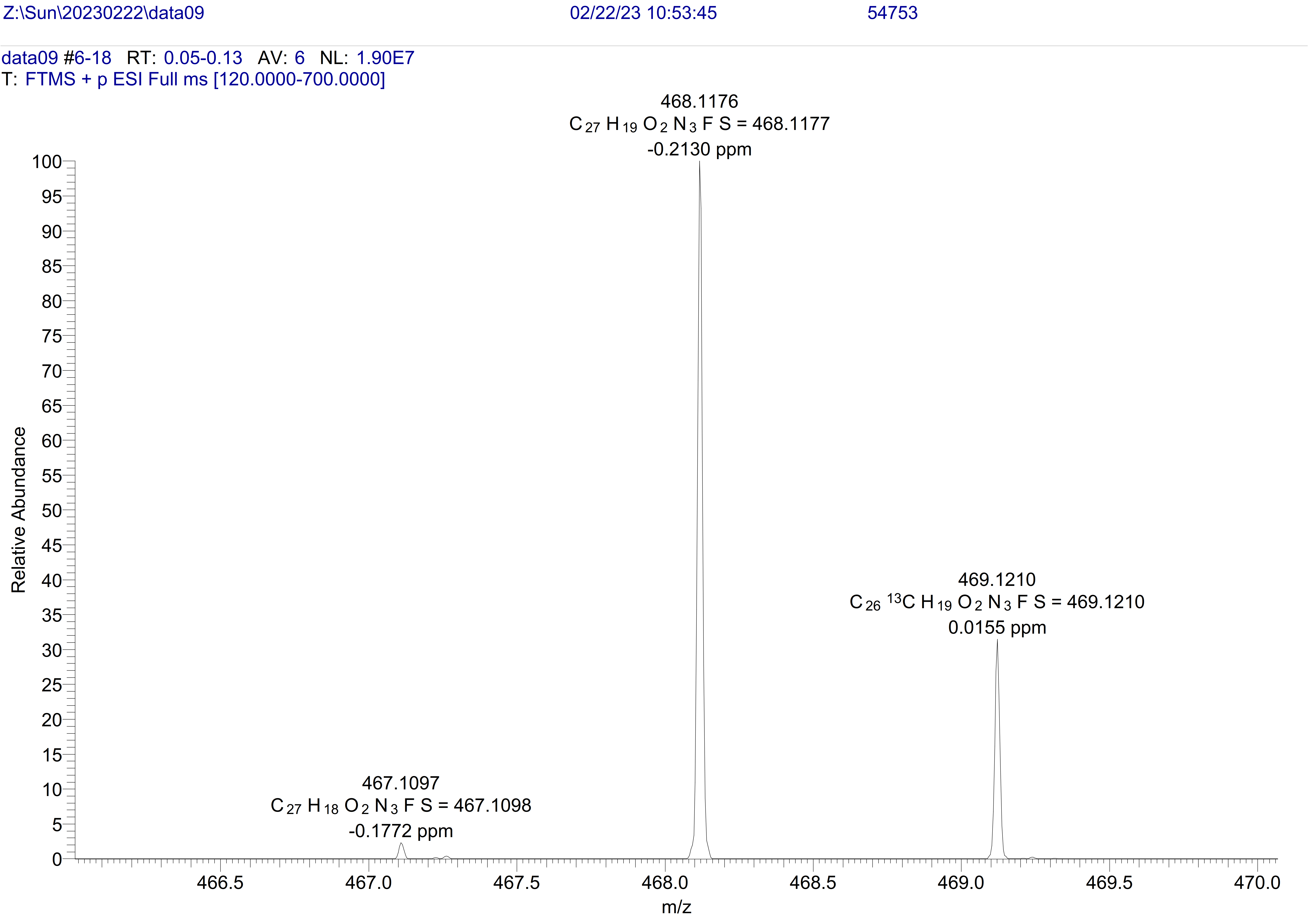


**Figure 41S.** High resolution ESI-MS spectrum of compound **19c**


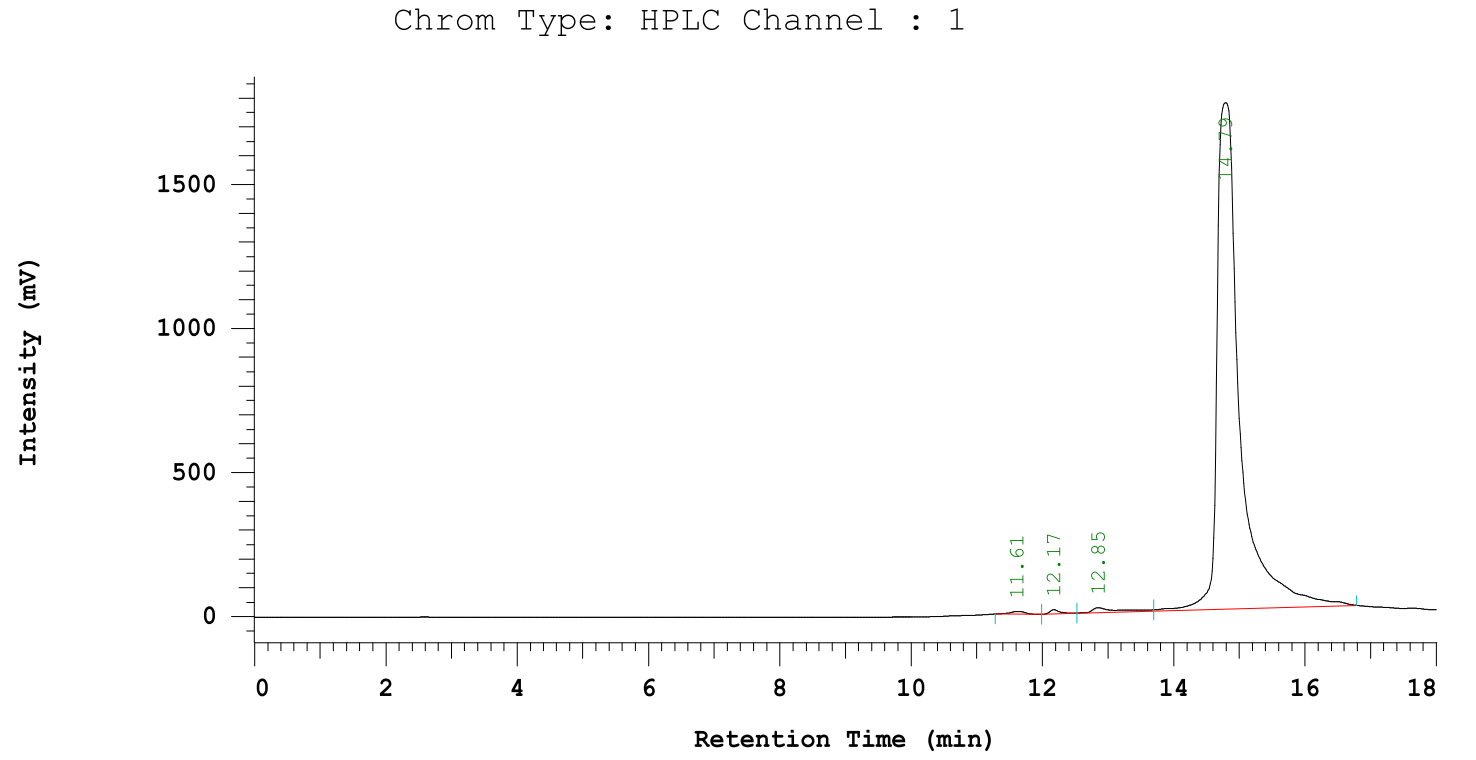


Retention time: 14.79 min

Flow rate: 1 mL/min

Detector wavelength: 250 nm

Mobile phase:

Purity: 98 %

**Figure 42S.** HPLC chromatogram of compound **7a**


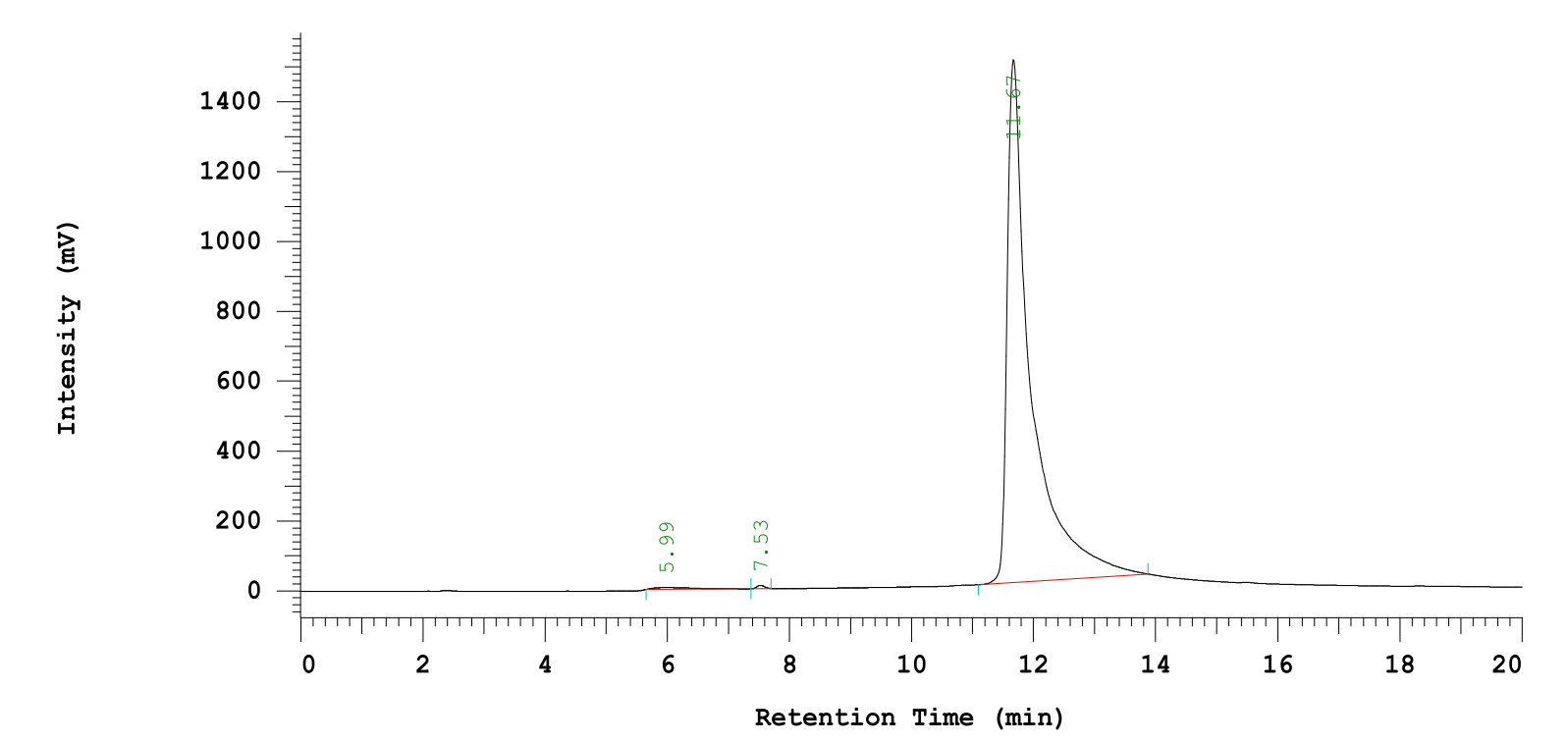


Retention time: 11.67 min

Flow rate: 1 mL/min

Detector wavelength: 250 nm

Mobile phase:

Purity: 99 %

**Figure 43S.** HPLC chromatogram of compound **7b**


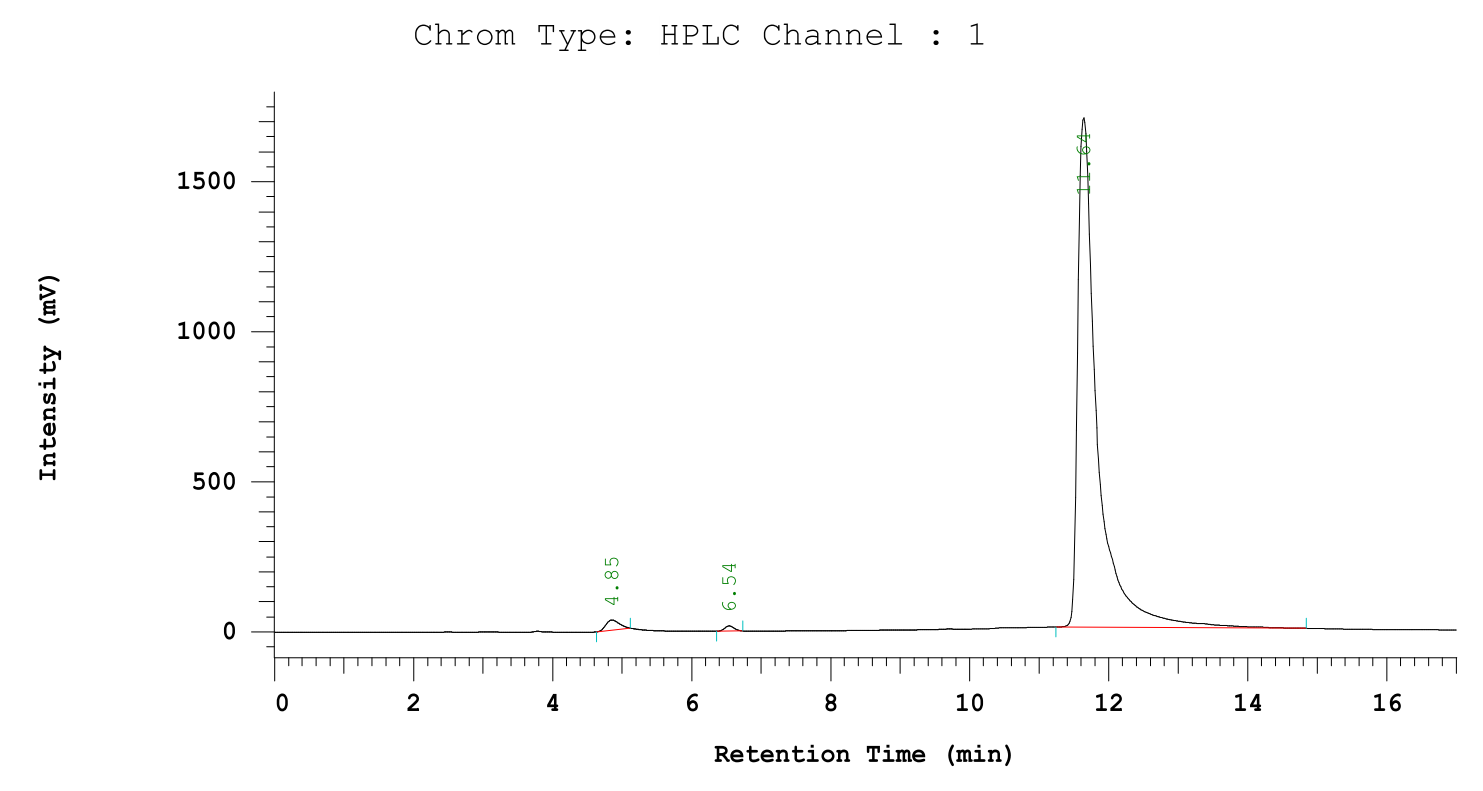


Retention time: 11.64 min

Flow rate: 1 mL/min

Detector wavelength: 250 nm

Mobile phase:

Purity: 98 %

**Figure 44S.** HPLC chromatogram of compound **7c**


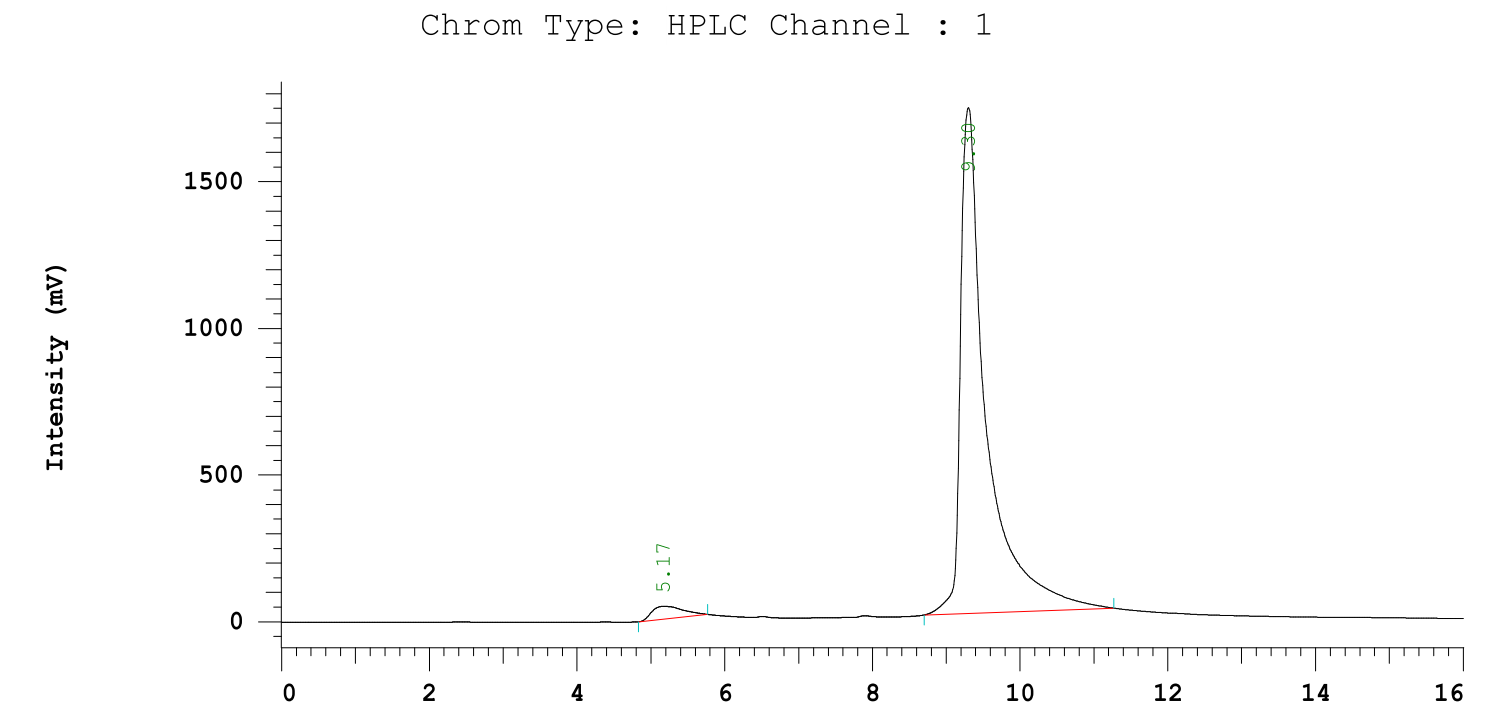


Retention time: 9.30 min

Flow rate: 1 mL/min

Detector wavelength: 250 nm

Mobile phase:

Purity: 97 %

**Figure 45S.** HPLC chromatogram of compound **7d**


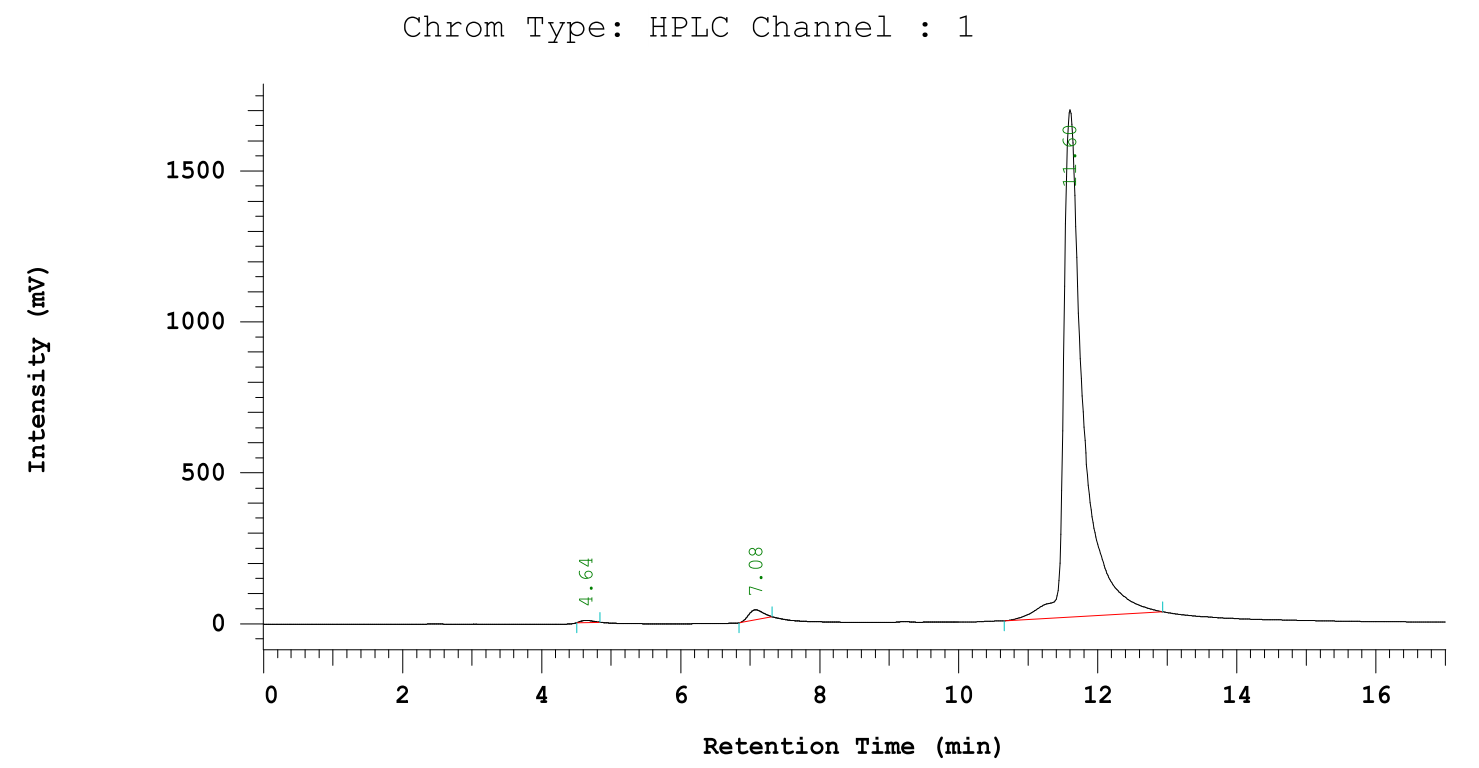


Retention time: 11.60 min

Flow rate: 1 mL/min

Detector wavelength: 250 nm

Mobile phase:

Purity: 98 %

**Figure 46S.** HPLC chromatogram of compound **7e**


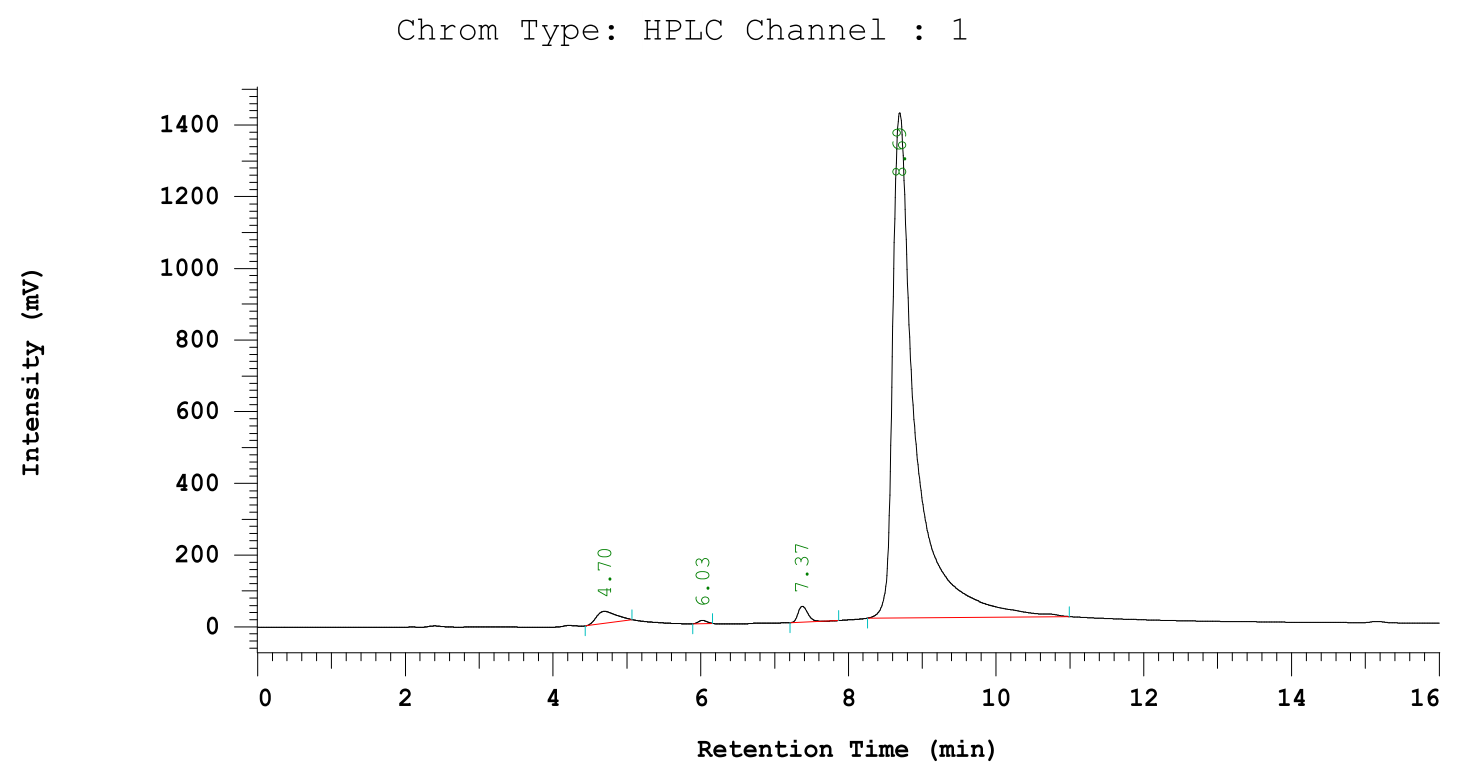


Retention time: 8.69 min

Flow rate: 1 mL/min

Detector wavelength: 250 nm

Mobile phase:

Purity: 97 %

**Figure 47S.** HPLC chromatogram of compound **7f**


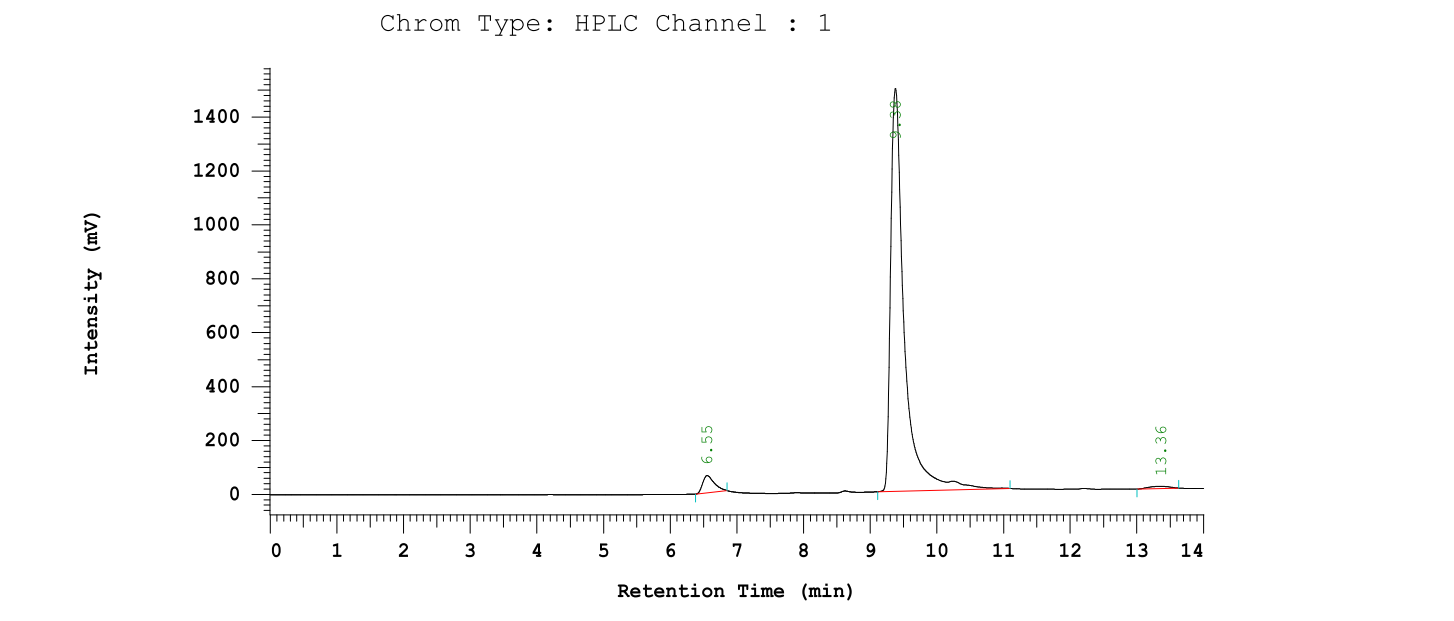


Retention time: 9.38 min

Flow rate: 1 mL/min

Detector wavelength: 250 nm

Mobile phase:

Purity: 96 %

**Figure 48S.** HPLC chromatogram of compound **7g**


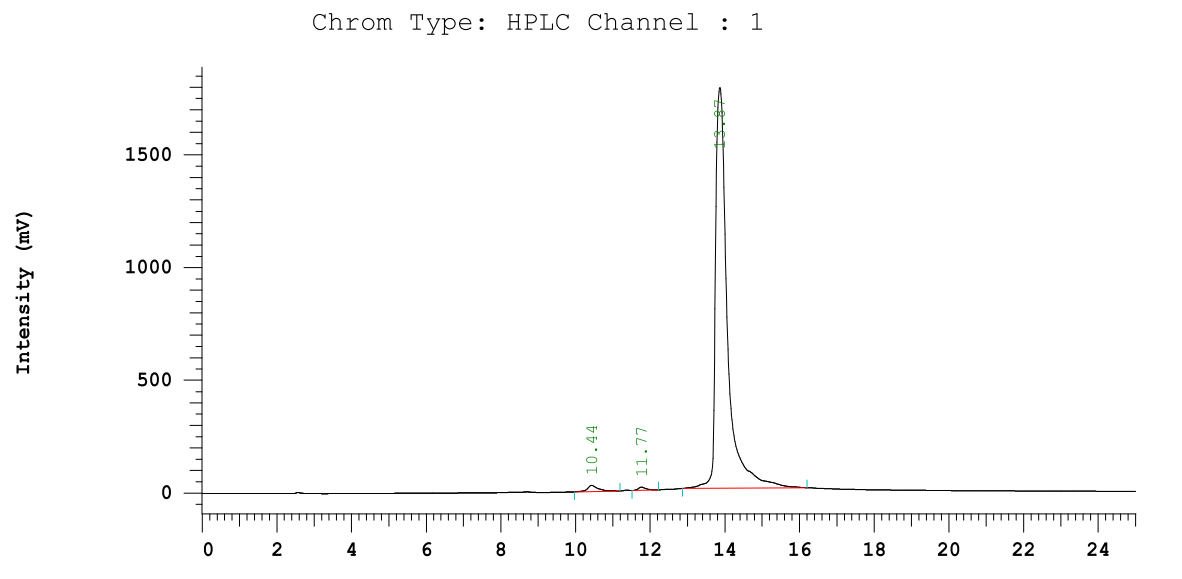


Retention time: 13.87 min

Flow rate: 1 mL/min

Detector wavelength: 250 nm

Mobile phase:

Purity: 98 %

**Figure 49S.** HPLC chromatogram of compound **7h**


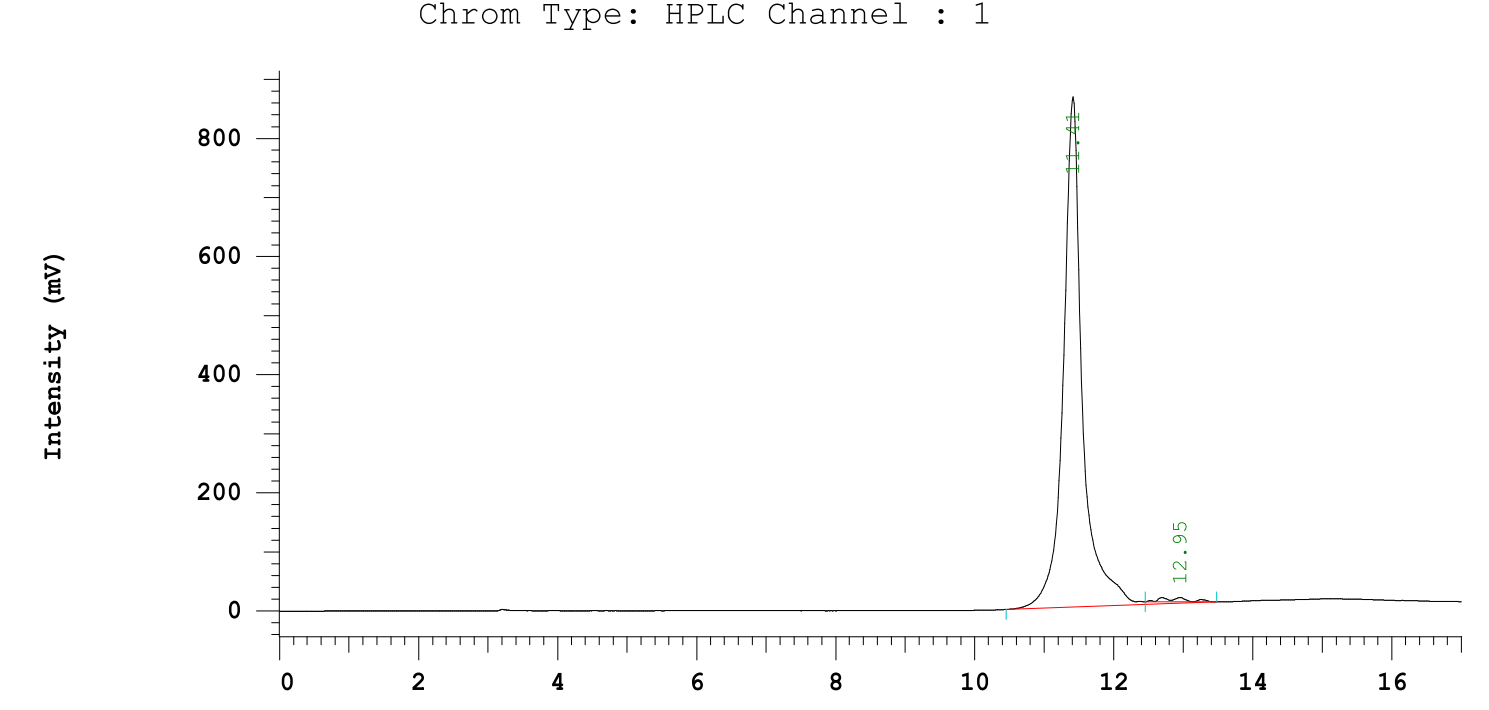


Retention time: 11.41 min

Flow rate: 0.8 mL/min

Detector wavelength: 250 nm

Mobile phase:

Purity: 99 %

**Figure 50S.** HPLC chromatogram of compound **7i**


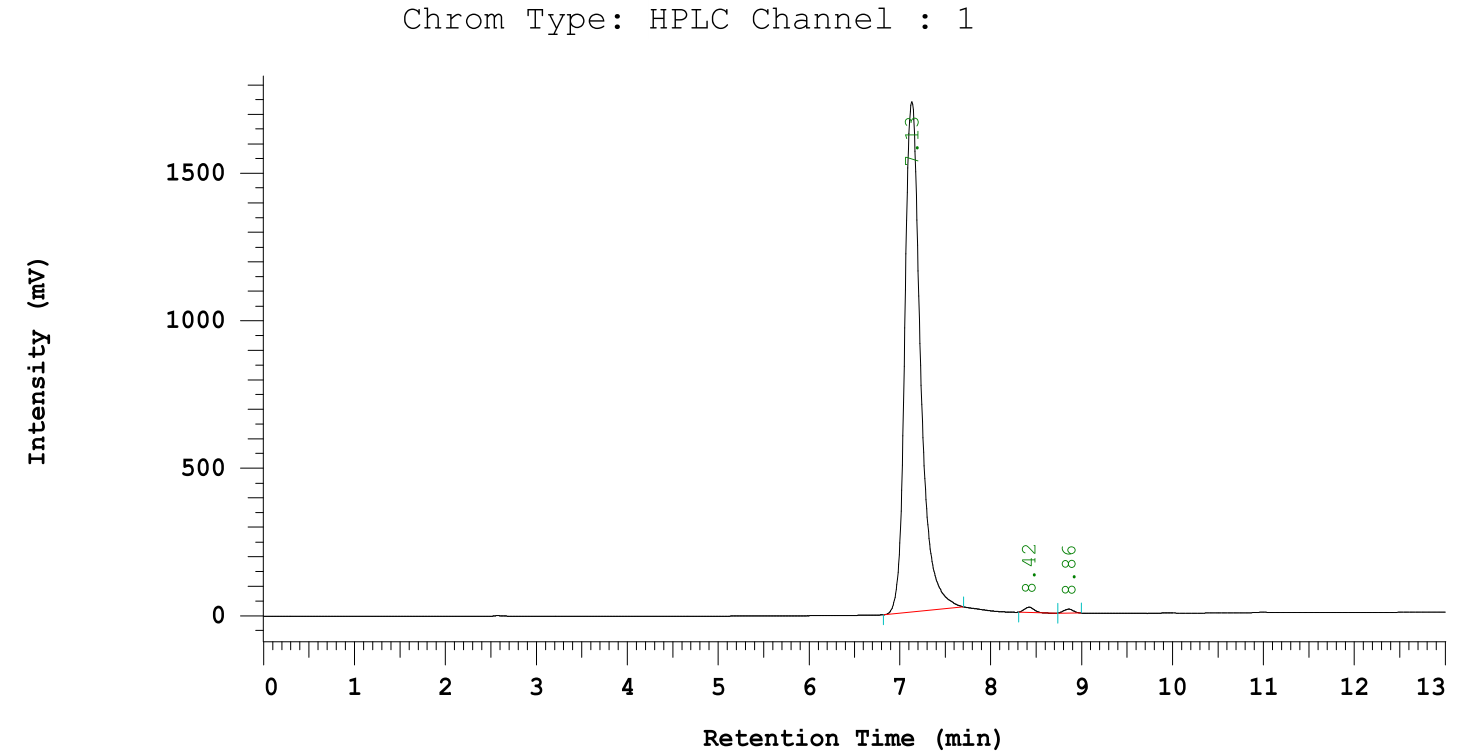


Retention time: 7.13 min

Flow rate: 1 mL/min

Detector wavelength: 250 nm

Mobile phase:

Purity: 99 %

**Figure 51S.** HPLC chromatogram of compound **7j**


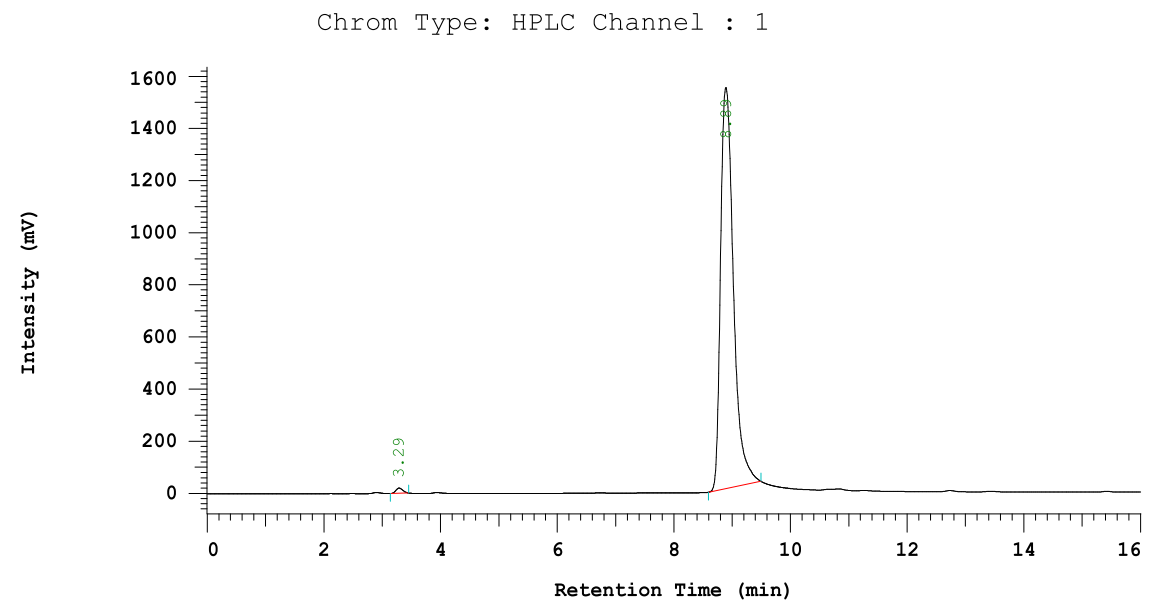


Retention time: 8.89 min

Flow rate: 1 mL/min

Detector wavelength: 250 nm

Mobile phase:

Purity: 99 %

**Figure 52S.** HPLC chromatogram of compound **19a**


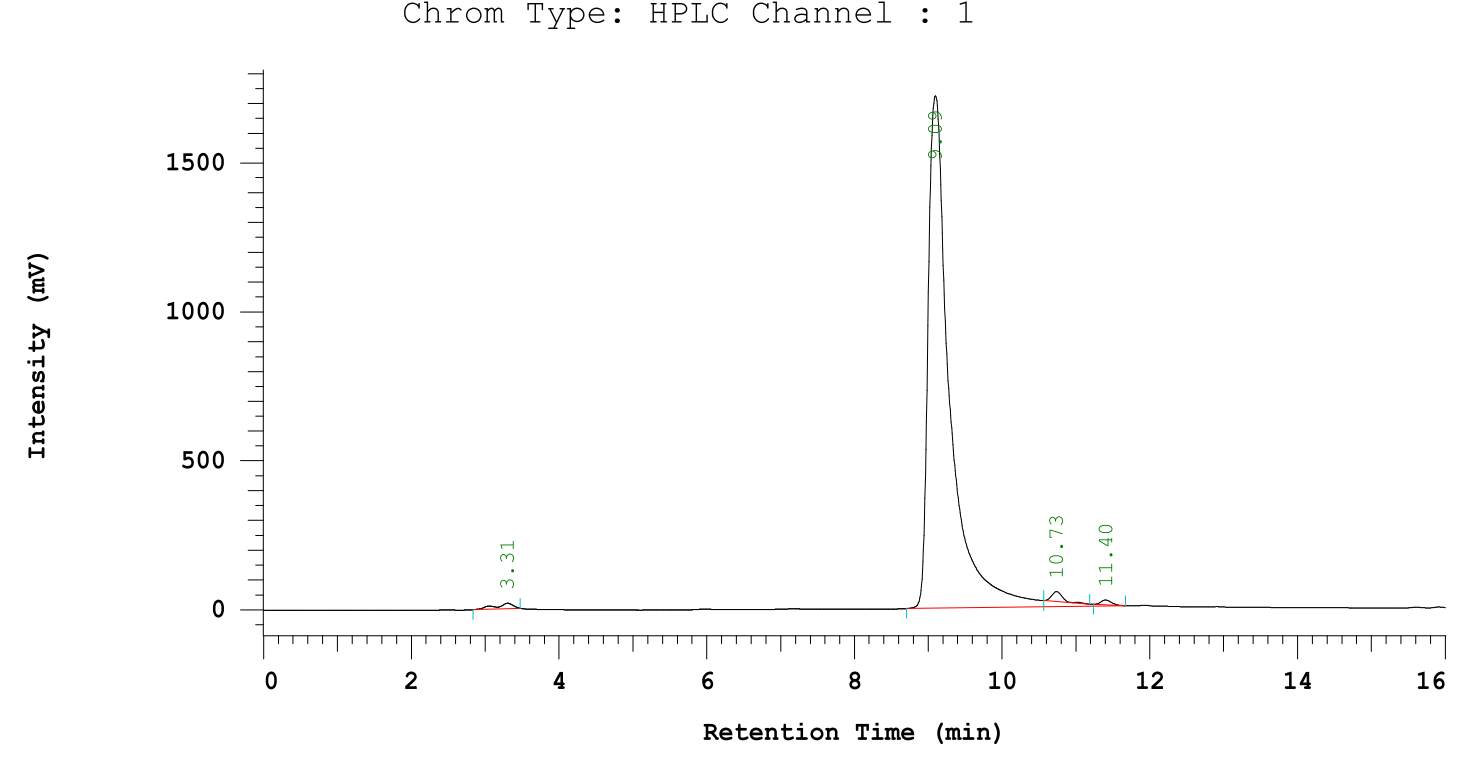


Retention time: 9.09 min

Flow rate: 1 mL/min

Detector wavelength: 250 nm

Mobile phase:

Purity: 98 %

**Figure 53S.** HPLC chromatogram of compound **19b**


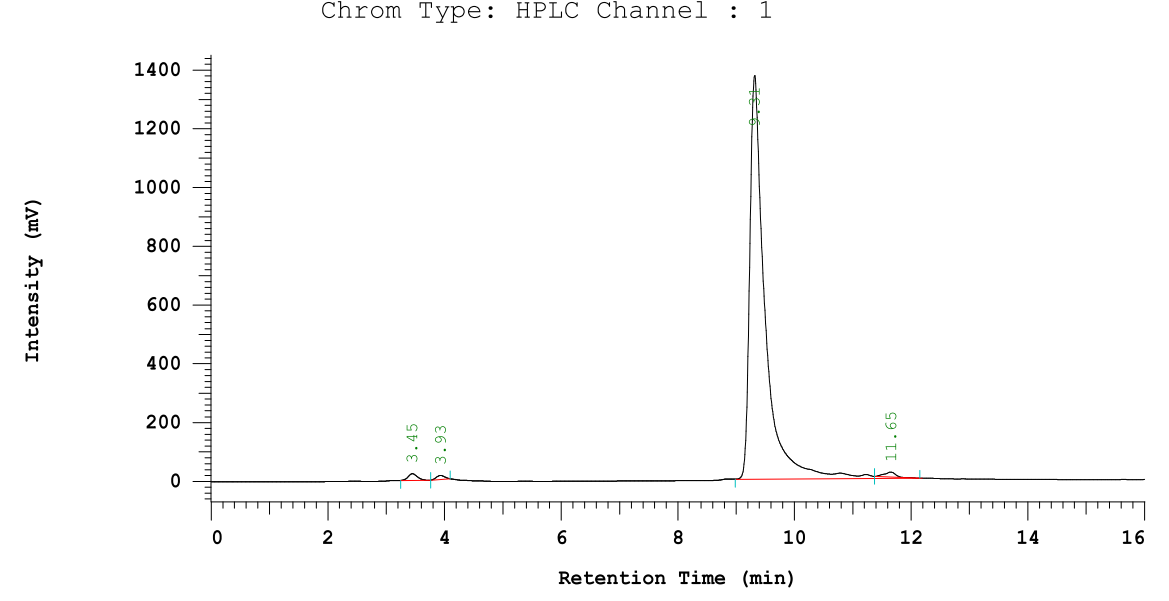


Retention time: 9.31 min

Flow rate: 1 mL/min

Detector wavelength: 250 nm

Mobile phase:

Purity: 98 %

**Figure 54S.** HPLC chromatogram of compound **19c**
